# Diarrhoeal disease and subsequent risk of death in infants and children residing in low-income and middle-income countries: analysis of the GEMS case-control study and 12-month GEMS-1A follow-on study

**DOI:** 10.1016/S2214-109X(19)30541-8

**Published:** 2019-12-18

**Authors:** Myron M Levine, Dilruba Nasrin, Sozinho Acácio, Quique Bassat, Helen Powell, Sharon M Tennant, Samba O Sow, Dipika Sur, Anita K M Zaidi, Abu S G Faruque, M Jahangir Hossain, Pedro L Alonso, Robert F Breiman, Ciara E O'Reilly, Eric D Mintz, Richard Omore, John B Ochieng, Joseph O Oundo, Boubou Tamboura, Doh Sanogo, Uma Onwuchekwa, Byomkesh Manna, Thandavarayan Ramamurthy, Suman Kanungo, Shahnawaz Ahmed, Shahida Qureshi, Farheen Quadri, Anowar Hossain, Sumon K Das, Martin Antonio, Debasish Saha, Inacio Mandomando, William C Blackwelder, Tamer Farag, Yukun Wu, Eric R Houpt, Jaco J Verweiij, Halvor Sommerfelt, James P Nataro, Roy M Robins-Browne, Karen L Kotloff

**Affiliations:** a Center for Vaccine Development and Global Health, University of Maryland School of Medicine, Baltimore, MD, USA; b Department of Medicine, University of Maryland School of Medicine, Baltimore, MD, USA; c Department of Pediatrics, University of Maryland School of Medicine, Baltimore, MD, USA; d Centro de Investigação em Saúde da Manhiça, Maputo, Mozambique; e Instituto Nacional de Saúde, Ministério de Saúde, Maputo, Mozambique; f ISGlobal, Hospital Clínic—Universitat de Barcelona, Barcelona, Spain; g Institució Catalana de Recerca i Estudis Avançats (ICREA), Barcelona, Spain; h Pediatric Infectious Diseases Unit, Pediatrics Department, Hospital Sant Joan de Déu (University of Barcelona), Barcelona, Spain; i Consorcio de Investigación Biomédíca en Red de Epidemiología y Salud Pública (CIBERESP), Madrid, Spain; j Centre pour le Développement des Vaccins, Bamako, Mali; k National Institute of Cholera and Enteric Diseases, Kolkata, India; l Department of Paediatrics and Child Health, Aga Khan University, Karachi, Pakistan; m Bill & Melinda Gates Foundation, Seattle, WA, USA; n International Centre for Diarrhoeal Disease Research, Mohakhali, Dhaka, Bangladesh; o Medical Research Council Unit The Gambia at the London School of Hygiene and Tropical Medicine, Banjul, The Gambia; p Global Malaria Programme, World Health Organization, Geneva, Switzerland; q Kenya Medical Research Institute/Centers for Disease Control and Prevention, Kisumu, Kenya; r Global Disease Detection Division, Kenya Office of the US Centers for Disease Control and Prevention, Nairobi, Kenya; s Global Health Institute, Emory University, Atlanta, GA, USA; t Division of Foodborne, Waterborne and Environmental Diseases, Centers for Disease Control and Prevention, Atlanta, GA, USA; u Centers for Disease Control and Prevention Country Office, Addis Ababa, Ethiopia; v London School of Hygiene and Tropical Medicine, Harar, Ethiopia; w Translational Health Science and Technology Institute, Faridabad, India; x PPD, San Diego, CA, USA; y Square Hospitals, Mohakhali, Dhaka, Bangladesh; z Menzies School of Health Research, Casuarina, NT, Australia; aa GlaxoSmithKline Vaccines, Wavre, Belgium; ab Department of Epidemiology and Public Health, University of Maryland School of Medicine, Baltimore, MD, USA; ac Institute of Health Metrics and Evaluation, University of Washington, Seattle, WA, USA; ad Sanofi Pasteur, Swiftwater, PA, USA; ae Division of Infectious Diseases and International Health, Department of Medicine, University of Virginia, Charlottesville, VA, USA; af Department of Parasitology, Leiden University Medical Center, Leiden, Netherlands; ag Elisabeth-TweeSteden Hospital, Tilburg, Netherlands; ah Centre for Intervention Science in Maternal and Child Health, Centre for International Health, Department of Global Public Health and Primary Care, University of Bergen, Bergen, Norway; ai Norwegian Institute of Public Health, Oslo, Norway; aj Department of Pediatrics, University of Virginia School of Medicine, Charlottesville, VA, USA; ak Department of Microbiology and Immunology, Peter Doherty Institute for Infection and Immunity, University of Melbourne, Melbourne, VIC, Australia

## Abstract

**Background:**

The Global Enteric Multicenter Study (GEMS) was a 3-year case-control study that measured the burden, aetiology, and consequences of moderate-to-severe diarrhoea (MSD) in children aged 0–59 months. GEMS-1A, a 12-month follow-on study, comprised two parallel case-control studies, one assessing MSD and the other less-severe diarrhoea (LSD). In this report, we analyse the risk of death with each diarrhoea type and the specific pathogens associated with fatal outcomes.

**Methods:**

GEMS was a prospective, age-stratified, matched case-control study done at seven sites in Africa and Asia. Children aged 0–59 months with MSD seeking care at sentinel health centres were recruited along with one to three randomly selected matched community control children without diarrhoea. In the 12-month GEMS-1A follow-on study, children with LSD and matched controls, in addition to children with MSD and matched controls, were recruited at six of the seven sites; only cases of MSD and controls were enrolled at the seventh site. We compared risk of death during the period between enrolment and one follow-up household visit done about 60 days later (range 50–90 days) in children with MSD and LSD and in their respective controls. Approximately 50 pathogens were detected using, as appropriate, classic bacteriology, immunoassays, gel-based PCR and reverse transcriptase PCR, and quantitative real-time PCR (qPCR). Specimens from a subset of GEMS cases and controls were also tested by a TaqMan Array Card that compartmentalised probe-based qPCR for 32 enteropathogens.

**Findings:**

223 (2·0%) of 11 108 children with MSD and 43 (0·3%) of 16 369 matched controls died between study enrolment and the follow-up visit at about 60 days (hazard ratio [HR] 8·16, 95% CI 5·69–11·68, p<0·0001). 12 (0·4%) of 2962 children with LSD and seven (0·2%) of 4074 matched controls died during the follow-up period (HR 2·78, 95% CI 0·95–8·11, p=0·061). Risk of death was lower in children with dysenteric MSD than in children with non-dysenteric MSD (HR 0·20, 95% CI 0·05–0·87, p=0·032), and lower in children with LSD than in those with non-dysenteric MSD (HR 0·29, 0·14–0·59, p=0·0006). In children younger than 24 months with MSD, infection with typical enteropathogenic *Escherichia coli*, enterotoxigenic *E coli* encoding heat-stable toxin, enteroaggregative *E coli, Shigella* spp (non-dysentery cases), *Aeromonas* spp, *Cryptosporidium* spp, and *Entamoeba histolytica* increased risk of death. Of 61 deaths in children aged 12–59 months with non-dysenteric MSD, 31 occurred among 942 children qPCR-positive for *Shigella* spp and 30 deaths occurred in 1384 qPCR-negative children (HR 2·2, 95% CI 1·2–3·9, p=0·0090), showing that *Shigella* was strongly associated with increased risk of death.

**Interpretation:**

Risk of death is increased following MSD and, to a lesser extent, LSD. Considering there are approximately three times more cases of LSD than MSD in the population, more deaths are expected among children with LSD than in those with MSD. Because the major attributable LSD-associated and MSD-associated pathogens are the same, implementing vaccines and rapid diagnosis and treatment interventions against these major pathogens are rational investments.

**Funding:**

Bill & Melinda Gates Foundation.

**Research in context**

**Evidence before this study**

Although diarrhoeal-associated deaths have steadily decreased during the past two decades, diarrhoeal diseases remain responsible for an estimated 9% of all mortality in children younger than 5 years, accounting for about 499 000 to 525 000 annual deaths. Accurate descriptions of the major causative pathogens, determinants, and risk factors for these deaths are necessary to design improved preventive and therapeutic strategies that will have maximal impact on diarrhoeal disease, and thus, on young child survival. We searched PubMed for articles published in English, Spanish, French, or Portuguese between Jan 1, 2000, and Sept 30, 2019, using the terms “low and middle-income countries” or “developing countries” AND “diarrheal deaths” OR “diarrhea AND mortality”. We included earlier reports, and articles identified in reference lists, as appropriate. Previous aetiological studies have put the focus on specific pathogens in particular high-risk populations, including, among others, rotavirus, diarrhoeagenic *Escherichia coli*, or *Shigella* spp. However, few studies have included comprehensive investigation of a representative sample of diarrhoea cases in children in different low-income regions of the world where diarrhoeal illness remains a major public health problem.

**Added value of this study**

Data from the GEMS study showed that during the approximately 60-day follow-up period, children with MSD had an 8·5 times increased risk of death compared with control children without diarrhoea. The GEMS-1A study corroborates the MSD risk of death (nearly 12 times increase in cases compared with controls) and further showed that children with LSD had a 2·8 times increased risk of death compared with controls, although this difference was not statistically significant. Somewhat surprisingly, children with non-dysenteric MSD had a substantially higher risk of dying than did children with dysenteric MSD; notably, most children with dysentery in the GEMS and GEMS-1A studies received anti-*Shigella* antibiotics in accordance with WHO standard of care recommendations. About half the deaths in children aged 12–59 months with non-dysenteric MSD were positive for *Shigella* spp when quantitative real-time PCR (qPCR) testing was performed. Notably, three-quarters of the qPCR-positive fatal non-dysenteric MSD cases had negative cultures for *Shigella* spp.

**Implications of all the available evidence**

Collectively, these observations provide a strong rationale for vigorously acting upon all severities of diarrhoeal illness. Additionally, the prominence of delayed deaths occurring 14 or more days after enrolment of cases calls for a need to ensure follow-up for several weeks beyond the initial management of the acute diarrhoeal episode. The main pathogens implicated in MSD-associated or LSD-associated deaths were found to be the same, thus supporting the need to pursue common preventive and therapeutic strategies for diarrhoea as a major threat to child survival.

## Introduction

The fourth Millennium Development Goal (MDG4), which aimed to reduce mortality in children younger than 5 years by two-thirds by 2015 compared with the 1990 baseline, triggered efforts to improve monitoring and to identify the predominant causes of paediatric deaths.^1^, ^2^ Although in 1990 south Asia's enormous annual birth cohort contributed the largest number of deaths in children younger than 5 years (4·78 million *vs* 3·77 million for sub-Saharan Africa), the highest under-5 mortality was reported in sub-Saharan Africa (177 per 1000 livebirths *vs* 126 per 1000 livebirths in south Asia).^1^, ^2^

To achieve MDG4, the major causes of under-5 mortality in these two geographical regions had to be reduced substantially. Early estimates identified diarrhoeal disease as the second most common cause of under-5 mortality after pneumonia, accounting for approximately 19% of deaths.^1^, ^3^ Subsequent analyses indicated that under-5 mortality was falling globally, including the proportion of post-neonatal child mortality (age 1–59 months) attributable to diarrhoeal disease.^4^, ^5^, ^6^

One post-2015 challenge is to accelerate the decrease in mortality from diarrhoeal disease so that by 2030 the overall under-5 mortality will not exceed 25 per 1000 livebirths in any country worldwide (part of Sustainable Development Goal [SDG] 3.2).^7^ Improved treatment of diarrhoeal disease, water and sanitation upgrades, and development and introduction of vaccines against major diarrhoeal disease agents associated with fatality could achieve this ambitious aim. Diarrhoeal mortality estimates from the 2016 Global Burden of Disease Study^8^ suggest that this goal is feasible, including in sub-Saharan Africa.^8^, ^9^

The Global Enteric Multicenter Study (GEMS) was done in the first decade of the millennium when diarrhoeal disease constituted the second most frequent cause of under-5 mortality and there was uncertainty regarding the relative mortality contribution of specific enteric pathogens.^10^, ^11^, ^12^ During the last quarter of the 20th century, many new diarrhoeal pathogens were identified and improved diagnostics in the first decades of the 21st century offered high-throughput options with increased sensitivity to detect these pathogens in individuals with diarrhoeal disease and in controls without diarrhoea.^10^

The 3-year GEMS case-control study identified aetiological agents associated with moderate-to-severe diarrhoea (MSD), assuming that cases of this severity might be fatal if the patient could not access health care.^11^ Children with MSD were enrolled at sentinel health centres that served demographically monitored populations so that pathogen-specific population-based incidence rates could be estimated.^12^, ^13^, ^14^ Matched controls without diarrhoea were selected among children in the community. The study reported adjusted attributable fractions for pathogens that took into account the presence of enteropathogens in matched controls without diarrhoeal disease and adjusted for the presence of multiple pathogens.^12^, ^13^, ^14^, ^15^ The GEMS study included a single household visit about 60 days after enrolment to ascertain the vital status of children with MSD and controls, and to assess linear growth since enrolment.^12^, ^13^ Thus, deaths that occurred among cases and controls during a prospective 60-day period were captured. The GEMS study identified four major pathogens associated with MSD (rotavirus, *Cryptosporidium* spp, enterotoxigenic *Escherichia coli* encoding heat-stable toxin [ST-ETEC] with or without co-expression of heat-labile enterotoxin, and *Shigella* spp) and showed that children with MSD had more linear growth faltering during the follow-up period than did controls.^12^ The GEMS study also showed that during the approximately 60-day follow-up period, MSD was associated with an 8·5 times increase in the odds of death compared with no diarrhoea.^12^ Many deaths in children with MSD occurred at home beyond the first week post-enrolment and would have been missed were it not for the 60-day visit to case and control households.

Children with MSD in the GEMS study accounted for only 24·1% of all children with diarrhoeal disease who presented to the sentinel health centres; no data were obtained from children seeking health care who had the more common less-severe diarrhoea (LSD) and who were therefore ineligible for enrolment into the GEMS study. Accordingly, a 1-year follow-on study, GEMS-1A, was done, consisting of two parallel case-control studies, one enrolling cases of MSD and matched controls and the other enrolling cases of LSD and matched controls.^16^ The GEMS-1A study assessed the overall and pathogen-specific, population-based, attributable incidence and the pathogen-specific attributable fraction, and the frequency of nutritional faltering in children with the two diarrhoeal syndromes. Outcomes were assessed by site and age stratum, and across all sites for incidence and nutritional outcomes. Because dysentery was an exclusion criterion for LSD, in the GEMS-1A study a non-dysentery MSD category was included to compare watery diarrhoea syndromes of differing severity.

Herein we report deaths in children with MSD (overall, dysenteric, and non-dysenteric) during 4 years of enrolment; deaths in children with MSD and LSD and in their matched controls during the two parallel GEMS-1A case-control studies; specific pathogens associated with fatal outcomes; deaths in cases and controls over time post-enrolment; and insights from verbal autopsies on potential causes of delayed diarrhoea-associated deaths.

## Methods

### Study design and participants

The 12-month GEMS-1A study of MSD and LSD used, with minor exceptions,^16^ the same clinical,^13^ epidemiological,^11^, ^13^ microbiological,^17^ data management, and analytical methods^14^ as the 3-year GEMS study of MSD.

Six of the original seven GEMS field sites, located in countries with moderate-to-high mortality of children younger than 5 years in Africa (Mali, Mozambique, The Gambia) and Asia (Bangladesh, India, Pakistan) participated in the GEMS-1A study; cases of MSD only were enrolled in Kenya during GEMS-1A. Each site performed a population census and maintained a demographic surveillance system (DSS) during the case-control study to estimate the median population in each age stratum.^12^, ^13^, ^16^ For case enrolment, sentinel health centres were selected where children included in the DSS sought care for diarrhoeal illnesses, as determined by Healthcare Utilization and Attitudes Surveys.^18^

Children aged 0–59 months belonging to the DSS population at each site who sought care at a sentinel health centre were screened for diarrhoea, defined as three or more loose stools during the previous 24 h. Each child with diarrhoea was assessed for eligibility. Episodes eligible for inclusion had to be new (onset after ≥7 diarrhoea-free days), acute (onset within the previous 7 days), and fulfil at least one of the following criteria for MSD: sunken eyes (confirmed by parent or caretaker as beyond normal); loss of skin turgor; intravenous hydration administered or prescribed; dysentery (visible blood in loose stools); or admission to hospital. The remaining non-MSD, new, acute diarrhoea episodes were eligible to be enrolled as LSD.^16^ To maintain a manageable work flow we enrolled the first eight or nine eligible children with MSD and LSD per age stratum per fortnight. Following a prespecified algorithm, we aimed to enrol one to three control children without diarrhoea for each enrolled MSD or LSD case, depending on how many cases were enrolled in a fortnight in a particular age-sex stratum at a study site;^12^ in six instances four controls were enrolled. Controls, matched to each individual case by age (±2 months for cases aged 0–11 months and ±4 months for cases aged 12–59 months), sex, and residence (same or nearby village or neighbourhood as the case) were randomly selected from the site's DSS database and enrolled within 14 days of the case. Potential controls who had diarrhoea in the preceding 7 days were ineligible.

Enrolment at GEMS sites began on Dec 1 (India), Dec 2 (Bangladesh), Dec 10 (The Gambia, Mozambique), and Dec 17 (Mali), 2007, Jan 31, 2008 (Kenya), and March 3, 2008 (Pakistan), and ended on March 3, 2011. GEMS-1A enrolment began on Oct 31, 2011, and ceased on Nov 14, 2012.

The clinical protocols for the GEMS and GEMS1-A studies were approved by ethics committees at the University of Maryland (Baltimore, MD, USA), and at every field site. Written informed consent was obtained from the parent or primary caretaker of each participant.

### Procedures

Study procedures for the GEMS and GEMS1-A studies have been described in detail elsewhere.^11^, ^12^, ^13^, ^16^, ^17^ Enteropathogens were identified in stool specimens collected at baseline from cases and controls.^16^, ^17^ Approximately 50 pathogens were detected using, as appropriate, classic bacteriology, immunoassays, gel-based PCR and reverse transcriptase PCR, and quantitative real-time PCR (qPCR).^16^, ^17^ The laboratory assays used are summarised in the clinical protocol (appendix pp 1–49). The GEMS-1A PCR methods for detecting *E coli* pathotypes are included in the appendix (pp 50–53). Specimens from a subset of GEMS cases and controls were also tested using a TaqMan Array Card that compartmentalised probe-based qPCR assays for 32 enteropathogens.^19^

Field workers made a single follow-up visit to households of cases and controls about 60 days after enrolment (window 50–90 days) to assess the child's vital status, capture interim medical events, and repeat anthropometric measurements.^13^, ^16^ Strictly speaking, deaths in cases during the 60-day follow-up period constitute an extended case fatality risk,^20^ while deaths in controls during the 60-day period constitute a mortality risk. For simplicity, we use the term risk of death for both cases and controls.^12^

When deaths in enrolled children were detected, a time was arranged (respecting mourning practices) to perform a verbal autopsy using a WHO questionnaire.^13^ For deaths occurring in health centres or hospitals, study-specific case report form data were collected to complement information gathered via verbal autopsy. Verbal autopsy data were reviewed as follows: after standardised training was done for all sites by the same person (QB), two local clinicians experienced in verbal autopsy and in international coding of causes of death independently reviewed the verbal autopsy forms and assigned up to two plausible causes of death, using the simplified coding system based on the International Classification of Diseases, 10th revision (ICD-10) recommended for verbal autopsy. If codes assigned by the two reviewers differed, a third clinician experienced in verbal autopsy served as arbiter to provide a definitive cause of death code.

### Statistical analysis

Sample size considerations and justifications have been described elsewhere.^12^ Cumulative risk of death was calculated by the Kaplan-Meier method. Between-group comparisons of risk of death used proportional hazards (Cox) regression; when unfeasible because of small numbers, simple risk ratios were estimated. When cases and their matched controls were compared, the analysis was stratified by case-control set.

Cumulative hazard curves from Kaplan-Meier risk of death analysis are shown for MSD cases during 4 years of enrolment. Pathogens associated with increased risk of death in MSD and LSD cases were identified using multiple Cox regression. Pathogens were included if there were at least five deaths that tested positive for the pathogen and if that pathogen was significantly (p<0·10) associated with death in crude analyses that controlled only for site. Pathogens were then eliminated from a model that adjusted for other pathogens until only those pathogens significantly (p≤0·05) associated with death remained. This model selection method was used in GEMS analyses.^12^, ^14^ Hazard ratios (HRs) and 95% CIs are presented for all pathogens considered in the crude and the final model. Baseline height-for-age or length-for-age (HAZ) scores at the Pakistan site versus other Asian sites were compared by two-sample *t* test. Pair-wise comparisons were two-sided; results with p values of 0·05 or lower were considered significant. We made no imputation of missing values or adjustment for multiple comparisons. SAS version 9.4 was used for all analyses apart from Kaplan-Meier cumulative hazard curves, which were estimated using R version 3.5.3.

### Role of the funding source

The funders of the study had no role in study design, data collection, data analysis, data interpretation, or writing of the report. The corresponding author had full access to all the data in the study and had final responsibility for the decision to submit for publication.

## Results

**Figure 1:**
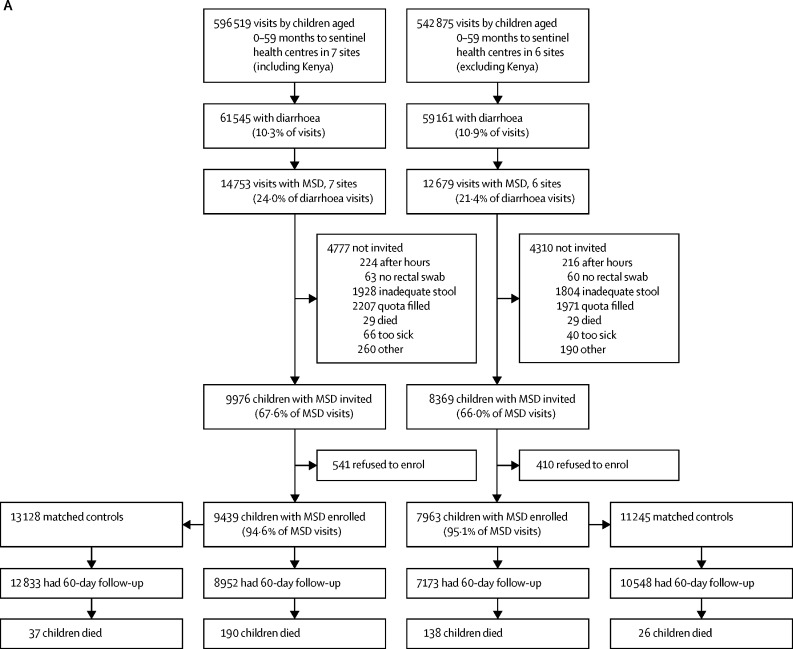

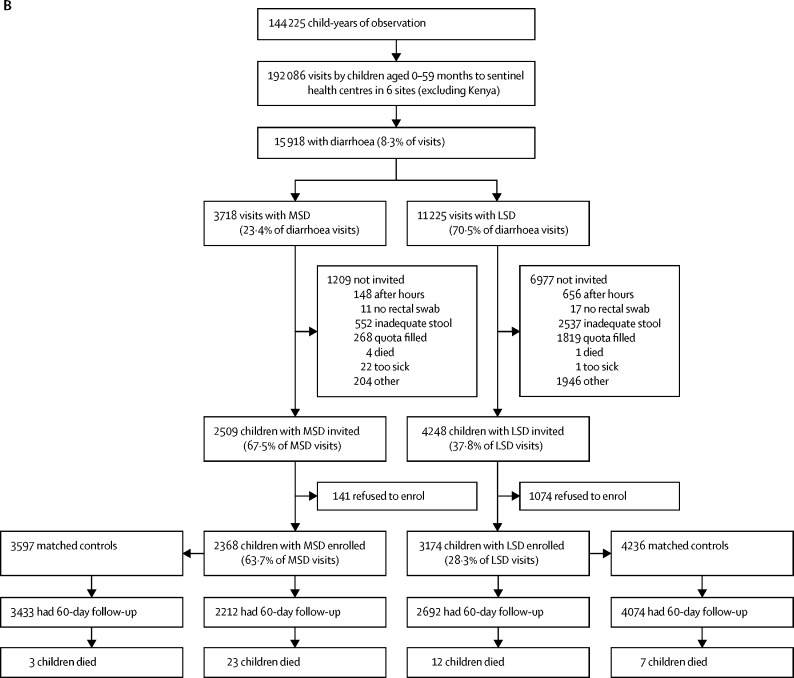

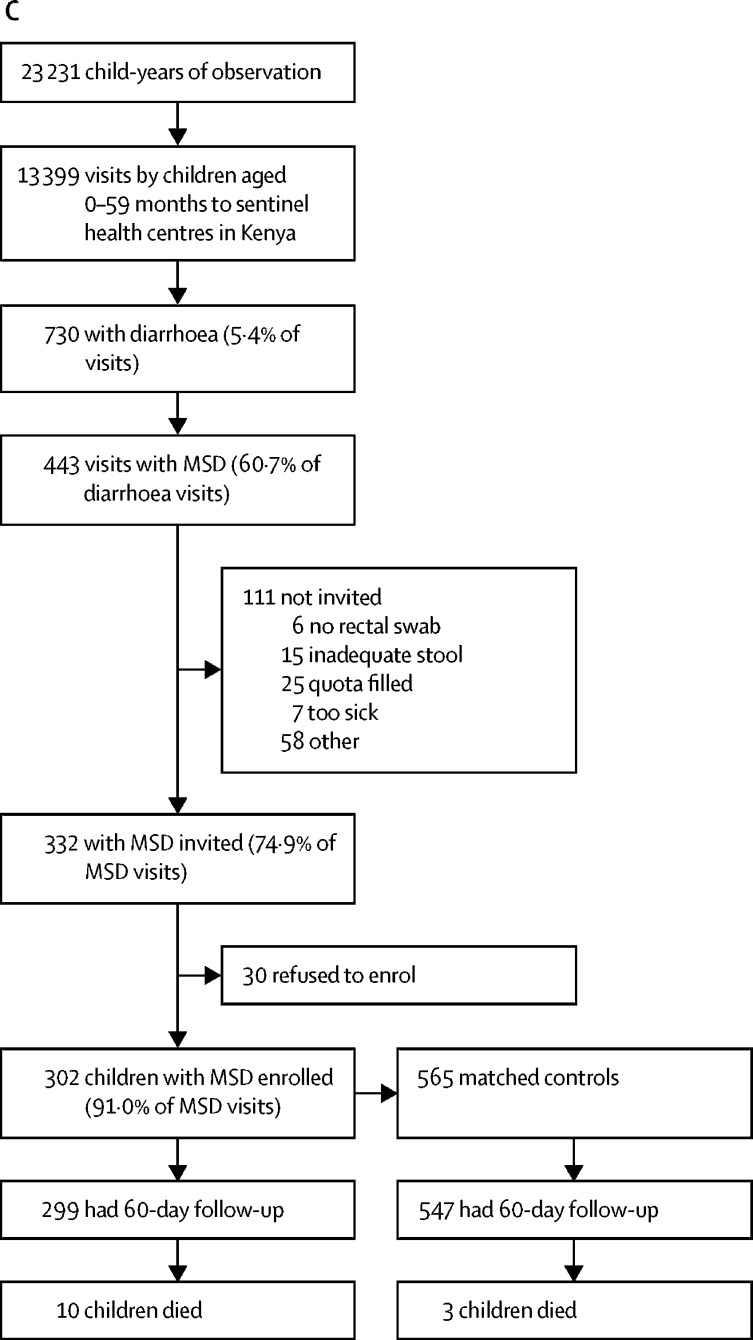
Study profiles for the GEMS and GEMS-1A studies (A) GEMS study enrolment of children aged 0–59 months with MSD and matched controls during 3 years at sentinel health centres at all seven GEMS sites (including Kenya) and at six sites (excluding Kenya). (B) GEMS-1A study enrolment of children aged 0–59 months during 1 year when cases of MSD, cases of LSD, and matched controls were enrolled at sentinel health centres at six sites (excluding Kenya). (C) GEMS-1A study enrolment of children aged 0–59 months with MSD and matched controls during 1 year at sentinel health centres at the Kenya site (LSD cases were not enrolled). MSD=moderate-to-severe diarrhoea. LSD=less-severe diarrhoea.

Figure 1 shows the numbers of enrolled cases and controls, the number of households that had a follow-up visit at about 60 days after enrolment, and deaths in all age strata combined for the GEMS study, GEMS-1A study, and Kenya during the GEMS-1A study where only MSD cases were enrolled. This information is displayed by age stratum in the appendix (pp 54–56).

**Figure 2:**
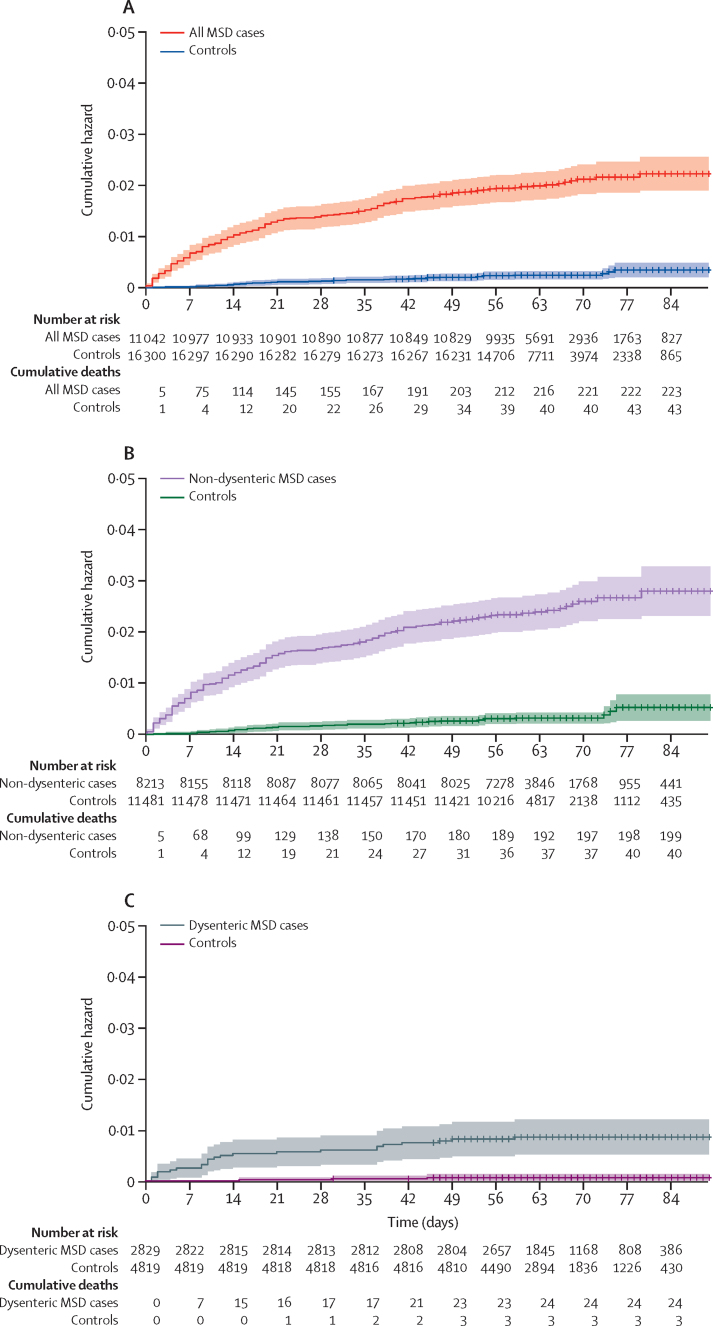
Cumulative hazard of death curves Shaded areas represent 95% CIs. Cumulative hazard of death curves, number at risk, and cumulative deaths comparing all MSD cases compared with controls (A), non-dysenteric MSD cases compared with controls (B), and dysenteric MSD cases compared with controls (C) during the approximately 60-day follow-up period (range 50–90 days) for the seven GEMS and GEMS-1A sites, during 4 years of enrolment. Data are for children who had a follow-up visit within the 50–90-day window for the day-60 follow-up visit and for whom there was information about whether or not they had died. MSD=moderate-to-severe diarrhoea.

Table 1 and figure 2 show the cumulative hazard of death for all MSD cases, non-dysenteric MSD cases, and dysenteric MSD cases, and their controls, during the 60-day post-enrolment period for all seven sites during 4 years of enrolment. All MSD cases and the MSD case subsets (dysentery or non-dysentery) show a significantly higher risk of death than their controls. Children with non-dysenteric MSD had a substantially higher risk of death (2·41 per 100 cases) than did children with dysenteric MSD (0·84 per 100 cases; p<0·0001). Table 1 also shows MSD deaths and risk of death by age stratum, with the heaviest burden in children younger than 24 months.

**Table 1:** Deaths among all MSD cases and non-dysenteric and dysenteric subgroups, and their matched controls, by site in both GEMS and GEMS-1A during 4 years of enrolment

	**MSD cases and their matched controls**				**Non-dysenteric MSD cases and their matched controls**				**Dysenteric MSD cases and their matched controls**			
	Cases	Controls	HR (95% CI)	p value	Cases	Controls	HR (95% CI)	p value	Cases	Controls	HR (95% CI)	p value
The Gambia	47/1238 (3·8%)	8/2036 (0·4%)	8·62 (3·82–19·42)	<0·0001	36/897 (4·0%)	8/1449 (0·6%)	6·20 (2·68–14·31)	<0·0001	11/341 (3·2%)	0/587	∞	..
Mali	25/2430 (1·0%)	5/2546 (0·2%)	7·67 (2·30–25·53)	<0·0009	22/2116 (1·0%)	5/2222 (0·2%)	6·67 (1·98–22·44)	<0·0022	3/314 (1·0%)	0/324	∞	..
Mozambique	59/725 (8·1%)	13/1550 (0·8%)	10·15 (5·27–19·55)	<0·0001	56/588 (9·5%)	13/1260 (1·0%)	9·60 (4·97–18·56)	<0·0001	3/137 (2·2)	0/290	∞	..
Kenya	62/1718 (3·6%)	14/2388 (0·6%)	6·07 (3·25–11·33)	<0·0001	59/1517 (3·9%)	12/2087 (0·6%)	7·05 (3·59–13·85)	<0·0001	3/201 (1·5%)	2/301 (0·7%)	1·19 (0·16–8·61)	0·087
India	2/2023 (0·1%)	1/2522 (<0·1%)	2·49* (0·33–19·02)	1·000	2/1764 (0·1%)	1/2167 (<0·1%)	2·46* (0·32–18·74)	1·000	0/259	0/355	∞	..
Bangladesh	7/1691 (0·4%)	1/3153 (<0·1%)	11·25 (1·37–92·40)	0·024	6/400 (1·5%)	0/760	∞	..	1/1291 (0·1%)	1/2393 (<0·1%)	1·85* (0·20–17·74)	..
Pakistan	21/1283 (1·6%)	1/2174 (<0·1%)	16·00 (2·12–120·65)	0·0071	18/982 (1·8%)	1/1595 (0·1%)	13·0 (1·70–99·35)	0·0134	3/301 (1·0%)	0/579	∞	..
All sites, age 0–59 months	223†/11 108 (2·0%)	43/16 369 (0·3%)	8·16 (5·69–11·68)	<0·0001	199/8264 (2·4%)	40/11 540 (0·3%)	7·95 (5·46–11·59)	<0·0001	24/2844 (0·8%)	3/4829 (0·1%)	10·38 (3·08–34·96)	0·0002
All sites, age 0–11 months	125/4621 (2·7%)	27/5978 (0·5%)	6·42 (4·10–10·06)	<0·0001	..	..	..	..	..	..	..	..
All sites, age 12–23 months	70/3768 (1·9%)	13/5484 (0·2%)	9·54 (4·87–18·70)	<0·0001	..	..	..	..	..	..	..	..
All sites, age 24–59 months	28/2719 (1·0%)	3/4907 (0·1%)	22·47 (5·29–95·41)	<0·0001	..	..	..	..	..	..	..	..

Data are number of deaths/total (%), unless otherwise stated. Also shown are the distributions of deaths in cases of MSD and deaths in controls by age stratum. MSD=moderate-to-severe diarrhoea. HR=hazard ratio.

* Because of the small number of deaths, for these analyses a simple risk ratio was estimated by a likelihood score method rather than HR by Cox regression.

† The 223 total deaths in children with MSD includes 97 (43·5%) girls and 126 (56·5%) boys, paralleling the enrolment distribution by sex of MSD cases (5280 [43·6%] girls, 6829 [56·4%] boys).

Of the 223 deaths in children with MSD, 110 (49·3%) occurred at least 14 days after enrolment and 79 (35·4%) occurred at least 21 days after enrolment. 193 (86·5%) of 223 deaths in children with MSD and 40 (93·0%) of 43 deaths in control children occurred in the four African sites. Risk of death estimates in controls at Asian sites were smaller than those observed at African sites, with only one death observed per site in controls during the 4 years. However, in children with MSD, the Pakistan site showed a unique pattern, with 21 deaths, resulting in a high HR of 16·00 (95% CI 2·12–120·65; table 1); no other Asian site had more than seven deaths in children with MSD. Because access to health care and treatment regimens were similar at the Asian sites, we hypothesised that children with MSD in Pakistan might have been more malnourished and thus at increased risk for a fatal outcome from MSD. Accordingly, we compared baseline HAZ scores in the 1594 children in Pakistan versus the 3821 children in India plus Bangladesh. Baseline HAZ in children in Pakistan (HAZ −2·03, 95% CI −2·10 to −1·95) was significantly lower than that in the other south Asian children (HAZ −1·31, −1·35 to −1·27; difference between means −0·72, 95% CI −0·79 to −0·64, p<0·0001).

In an analysis of data from six sites of the study (excluding Kenya), children with MSD were more likely to die than their matched controls during the GEMS study (HR 8·89, 95% CI 5·55–14·22) and GEMS-1A study (HR 11·91, 3·50–40·53; both p<0·0001; table 2). Children with dysenteric MSD at these six sites during the GEMS-1A study were less likely to die during the follow-up period than were children with non-dysenteric MSD (HR 0·20, 95% CI 0·05–0·87, p=0·032), as also observed during the GEMS study at those six sites (HR 0·41, 0·25–0·67, p=0·0003).

**Table 2:** Risk of death during 60 days of follow-up at six sites (The Gambia, Mali, Mozambique, India, Bangladesh, and Pakistan)

	Number of deaths/total*(%)	**HR (95% CI)**	**p value**
**GEMS MSD study**			
Cases	138†/7178 (1·9%)	8·89 (5·55–14·22)	<0·0001
Controls	26†/10 548 (0·3%)	1 (ref)	..
**GEMS MSD study**			
Dysenteric cases	19†/1952 (1·0%)	0·41 (0·25–0·67)	0·0003
Non-dysenteric cases	119†/5226 (2·3%)	1 (ref)	..
**GEMS-1A MSD study**			
Cases	23/2212 (1·0%)	11·91 (3·50–40·53)	<0·0001
Controls	3/3433 (0·1%)	1 (ref)	..
**GEMS-1A LSD study**			
Cases	12‡/2962 (0·4%)	2·78 (0·95–8·11)	0·061
Controls	7/4074 (0·2%)	1 (ref)	..
**GEMS-1A MSD study**			
Dysenteric cases	2/691 (0·3%)	0·20 (0·05–0·87)	0·032
Non-dysenteric cases	21/1521 (1·4%)	1 (ref)	..
**Intra-GEMS-1A comparison of MSD versus LSD**			
GEMS-1A MSD study cases	23/2212 (1·0%)	2·57 (1·28–5·17)	0·0080
GEMS-1A LSD study cases	12‡/2962 (0·4%)	1 (ref)	..
**Intra-GEMS-1A comparison of LSD versus non-dysenteric MSD**			
GEMS-1A LSD study cases	12‡/2962 (0·4%)	0·29 (0·14–0·59)	0·0006
GEMS-1A MSD study non-dysenteric cases	21/1521 (1·4%)	1 (ref)	..

HR=hazard ratio. MSD=moderate-to-severe diarrhoea. LSD=less-severe diarrhoea.

* The number of participants with follow-up information and therefore for whom health status (deceased or not) was known.

† These numbers exclude GEMS MSD data from Kenya.

‡ The 12 deaths in children with LSD include five (41·6%) girls and seven (58·3%) boys, paralleling the sex distribution of enrolled LSD cases (1492 [47·0%] girls, 1682 [53·0%] boys).

Risk of death per 100 cases in the GEMS-1A study was significantly higher in children with MSD than in those with LSD (HR 2·57, 95% CI 1·28–5·17, p=0·0080; table 2). Nonetheless, risk of death was greater in children with LSD than in their matched controls, although not significantly (HR 2·78, 95% CI 0·95–8·11, p=0·061). Because LSD excludes clinical dysentery, we compared the risk of death in children with LSD versus that in children with non-dysenteric MSD, and found the risk of death to be significantly lower in children with LSD (HR 0·29, 0·14–0·59, p=0·0006; table 2).

In Cox regression analysis, in all infants aged 0–11 months with MSD in the GEMS and GEMS-1A studies combined, typical enteropathogenic *E coli* (tEPEC), ST-ETEC, and *Aeromonas* spp infections were associated with increased risk of death (table 3), as were *Cryptosporidium*, tEPEC, enteroaggregative *E coli*, and *Entamoeba histolytica* infection in toddlers aged 12–23 months; the HR changed only minimally when controlled for breastfeeding (exclusive [defined as ingesting only breastmilk] *vs* non-exclusive; see appendix p 57 for HRs when controlled for breastfeeding). Deaths in infants with tEPEC were clustered within the first 3 months of life. In infants younger than 3 months who were not exclusively breastfed, the tEPEC isolation rate was twice as high (27 [17·2%] of 157) as it was in those who were exclusively breastfed (16 [7·1%] of 227; odds ratio 2·74, 95% CI 1·42–5·28; χ^2^ p=0·0026).

**Table 3:** Specific pathogens associated with a higher risk of fatal outcomes in infants and toddlers with MSD during GEMS and GEMS-1A, by age group

	**Number of deaths with pathogen present/total (%)**	**Number of deaths with pathogen absent/total (%)**	**HR (95% CI)**	**p value**
**0–11 months**				
Typical EPEC	29/431 (6·7%)	96/4190 (2·3%)	2·58 (1·69–3·93)	<0·0001
ST/LT ETEC and ST-only ETEC	15/290 (5·2%)	110/4331 (2·5%)	1·92 (1·12–3·32)	0·019
*Aeromonas* spp	6/251 (2·4%)	119/4371 (2·7%)	3·28 (1·27–8·51)	0·015
**12–23 months**				
*Cryptosporidium* spp	20/418 (4·8%)	50/3344 (1·5%)	2·14 (1·26–3·65)	0·0050
Typical EPEC	9/266 (3·4%)	61/3502 (1·7%)	2·35 (1·15–4·81)	0·019
Enteroaggregative *Escherichia coli*	19/645 (2·9%)	51/3123 (1·6%)	2·14 (1·24–3·70)	0·0063
*Entamoeba histolytica*	5/114 (4·4%)	65/3648 (1·8%)	3·46 (1·24–9·64)	0·018

Data include all 4 years of enrolment at seven sites during GEMS and GEMS-1A. MSD=moderate-to-severe diarrhoea. EPEC=enteropathogenic *Escherichia coli*. ST/LT ETEC=enterotoxigenic *E coli* encoding heat-stable toxin with co-expression of heat-labile enterotoxin. ST-only ETEC=enterotoxigenic *E coli* encoding heat-stable toxin without co-expression of heat-labile enterotoxin.

Evidence of higher risk of death in children with non-dysenteric MSD than in those with dysenteric MSD (Table 1, Table 2), recognition that culture alone may miss *Shigella* spp infections detectable by qPCR, and qPCR data from a large random subset of cases from the GEMS study^19^ led us to analyse those data, including qPCR results for 153 (91·1%) of the 168 children with non-dysenteric MSD who died in the GEMS study.

Among 61 deaths in children aged 12–59 months who had non-dysenteric MSD, 31 occurred in 942 children who were *Shigella*-positive by qPCR and 30 deaths occurred in 1384 children qPCR-negative for *Shigella* spp (HR 2·2, 95% CI 1·2–3·9, p=0·0090 by Cox regression analysis). Thus, *Shigella* in children with non-dysenteric MSD was strongly associated with an increased risk of death. Notably, only eight (25·8%) of these 31 fatal infections positive for *Shigella* spp by qPCR were also positive by culture, while 23 (74·2%) were culture negative.

Altogether, 285 deaths occurred among cases or controls in the GEMS study (190 cases, 37 controls) and GEMS-1A study (45 cases, 13 controls), and verbal autopsy data were available for 266 (93·3%; table 4). Because verbal autopsy interpretation allowed death to have up to two co-primary diagnoses, total percentages exceeded 100%. Of 230 deaths with verbal autopsy results, 114 (49·6%) were attributed to diarrhoeal disease, 26 (11·3%) to sepsis, 19 (8·3%) to pneumonia or respiratory infections, and 21 (9·1%) to severe malnutrition. Most deaths could be categorised either as directly attributable to diarrhoeal disease or related to sequelae of diarrhoea, including malnutrition and increased risk of secondary bacterial infections.

**Table 4:** Results of the verbal autopsy re-examination exercise

	**The Gambia**	**Mali**	**Mozambique**	**Kenya**	**India**	**Bangladesh**	**Pakistan**	**Total**
**Number of deaths**								
GEMS deaths cases	39	23	51	52	2	7	16	190
GEMS deaths controls	7	5	11	11	1	1	1	37
GEMS-1A deaths cases	11	3	11	10	0	2	8	45
GEMS-1A deaths controls	3	1	3	3	1	1	1	13
Total deaths in study	60	32	76	76	4	11	26	285
Total deaths with verbal autopsy re-reviewed results	58	31	76	66	4	11	20	266
Discrepancies between physicians	8/58 (13·8%)	5/31 (16·1%)	20/76 (26·3%)	10/66 (15·2%)	0/4	0/11	7/20 (35·0%)	50/266 (18·8%)
Causes of death*								
Diarrhoeal diseases (A09)	29/50 (58·0%)	15/26 (57·7%)	28/63 (44·4%)	22/55 (40·0%)	2/2 (100%)	8/9 (88·9%)	10/25 (40·0%)	114/230 (49·6%)
Acute respiratory infection, including pneumonia (J18)	4/50 (8·0%)	6/26 (23·1%)	4/63 (6·3%)	1/55 (1·8%)	1/2 (50·0%)	1/9 (11·1%)	3/25 (12·0%)	19/230 (8·3%)
HIV/AIDS-related death (B24)	0/50	1/26 (3·8%)	20/63 (31·7%)	21/55 38·2%)	0/2	0/9	0/25	42/230 (18·3%)
Severe malnutrition (E46)	14/50 (28·0%)	2/26 (7·7%)	2/63 (3·2%)	2/55 (3·6%)	1/2 (50·0%)	0/9	0/25	21/230 (9·1%)
Sepsis (including neonatal) (A41 and P36)	8/50 (16·0%)	4/26 (15·4%)	6/63 (9·5%)	0/55	0/2	5/9 (55·6%)	3/25 (12·0%)	26/230 (11·3%)
Malaria (B54)	3/50 (6·0%)	4/26 (15·4%)	2/63 (3·2%)	12/55 (21·8%)	0/2	0/9	1/25 (4·0%)	22/230 (9·6%)

* Causes of death (according to verbal autopsy) as primary or co-primary diagnosis (ICD-10 codes) in GEMS and GEMS-1A moderate-to-severe or less-severe diarrhoea cases only. Each patient may have up to two different diagnoses; therefore, numbers may exceed 100%.

## Discussion

A hallmark observation from the GEMS study was that children with MSD had an 8·5 times higher likelihood of a fatal outcome during the approximately 60-day follow-up period compared with control children without diarrhoea.^12^ This finding has now been corroborated by data from the GEMS-1A study (12 times increased risk of death in children with MSD *vs* matched controls).

In the combined MSD dataset from the GEMS and GEMS-1A studies, approximately half of MSD-associated deaths occurred following discharge from the enrolment sentinel health centre and more than 2 weeks after enrolment, and would not have been detected had there not been the systematic single follow-up visit to the household at about 60 days after enrolment. These observations imply that within the reference population, the burden of MSD-related deaths far exceeds deaths detected within the first days of onset of MSD episodes as observed in sentinel health centres.

Children with MSD in the GEMS-1A study had a significantly higher risk of death during the 60-day follow-up period than did children with LSD, an observation that answers unequivocally the query about whether the GEMS definition of MSD truly encompasses illnesses with a greater likelihood of fatal outcome than LSD for the individual child.^21^ Nonetheless, findings from the GEMS-1A study also show that the risk of death in children with LSD is approximately 2·8 times higher than in controls without diarrhoea. A larger study with greater statistical power might have shown LSD to be significantly associated with risk of death. Consequently, for a similar number of deaths to accrue among LSD cases as among MSD cases, it would take approximately 2·5 times the number of LSD cases. Because the number of children with LSD attending sentinel health centres in the GEMS-1A study was around three times the number of children with MSD, this implies that in the reference population more deaths overall occur in children with LSD.

Higher numbers of deaths occurred among controls in African sites than in Asian sites, reflecting the higher risk of death in children younger than 5 years in sub-Saharan Africa. Yet the highest risk of death in children with MSD was in Pakistan. In view of similar access to care and treatment regimens for MSD at all three Asian sites, we explored whether host nutritional differences (baseline nutritional state) might have contributed to the high risk of death in children with MSD in Pakistan. Indeed, children with MSD in Pakistan had significantly more linear growth faltering at baseline than did children with MSD in India and Bangladesh.

Several pathogens associated with increasing the risk of death from MSD in the GEMS study (ST-ETEC, tEPEC, and *Cryptosporidium* spp) were similarly identified in the GEMS-1A study, making these robust observations over multiple years of surveillance. ST-ETEC can rapidly dehydrate young infants leading to death, unless effective rehydration is promptly instituted. tEPEC and *Cryptosporidium* spp induce similar attaching and effacing histopathological lesions in small intestinal mucosa characterised by intimate adherence to enterocytes, effaced microvilli, and actin polymerisation;^22^, ^23^, ^24^ gut dysfunction associated with these intestinal lesions might increase risk of death.^20^ Infants with tEPEC who died showed classic risk factors associated with severe and fatal disease (ie, early infancy and lack of exclusive breastfeeding).^25^ Other pathogens identified as increasing risk of death were *Aeromonas* spp (infants) and *E histolytica* and enteroaggregative *E coli* (toddlers).

At first glance, a higher risk of death in children with non-dysenteric MSD than in those with dysenteric MSD might seem incongruous given the mucosal destruction that occurs with dysentery. However, dysentery (gross blood in diarrhoeal stools) is a clinical indication for treatment with anti-*Shigella* antibiotics, and most patients with dysenteric MSD in the GEMS and GEMS-1A studies received antibiotics. Thus, we hypothesise that antibiotic treatment lowered the risk of death from dysenteric MSD compared with non-dysenteric MSD. We also explored whether *Shigella* spp might be incriminated more strongly if we used molecular diagnostics rather than just culture, assuming the burden of *Shigella* spp in children with non-dysenteric MSD is likely to be underestimated. In the GEMS study, we had qPCR data from a large random subset of cases and controls that allowed us to identify many *Shigella*-associated cases that had negative stool cultures.^19^ In children aged 12–59 months with non-dysenteric MSD in the GEMS study, qPCR positivity for *Shigella* spp was strongly associated with increased risk of a fatal outcome. These data reinforce the need for *Shigella* vaccines and for an affordable, sensitive, and specific point-of-care test to diagnose *Shigella* spp in patients with non-dysenteric MSD to allow targeted, rational antibiotic treatment.

Because the occurrence of delayed (≥14 days post-enrolment) MSD-associated deaths observed in the GEMS study has been substantiated by findings in the GEMS-1A study and an increased risk of death (albeit lower) has been observed in children with LSD, we must ponder what is responsible for the deaths. Causal pathways that could plausibly culminate in delayed fatality include (1) dehydration worsening or recurring after discharge from the sentinel health centre, inadequate oral rehydration at home, and failure to return the child promptly to health care; (2) bacteraemia secondary to mucosal injury triggering over-stimulation of the systemic innate immune system leading to systemic inflammatory response syndrome (also known as sepsis syndrome);^26^ (3) gut microbiome alterations consequent to MSD and LSD allowing translocation and bloodstream invasion by opportunistic bacterial pathogens even if the intestinal mucosa remains intact, whereupon clinical sepsis can ensue; (4) severe gut dysfunction resulting in malabsorption and anorexia, leading to malnutrition, micronutrient deficiency and, ultimately, death; (5) rarely, progression to persistent diarrhoea, a harbinger for fatal outcomes;^27^ and (6) an increased risk of pneumonia or other severe infections following diarrhoeal disease.^28^

Analysis of verbal autopsy data from children who died in the African and Asian sites of the GEMS study shows that the most attributed causes of death coincide with the above possibilities, even if information for the Asian sites is limited because of the scarcer number of deaths occurring there. Nevertheless, verbal autopsy, while valuable, is an imperfect tool for interpreting the cause of death at the individual level. More definitive investigative tools are needed.^29^ Underlying HIV-1 infection, malaria, or severe malnutrition might render children more susceptible to fatality following MSD or LSD, whatever the aetiological agent responsible for their diarrhoea. It is hoped that the evidence of delayed deaths following MSD and LSD will stimulate innovative clinical and epidemiological investigations to elucidate the major precipitating causes of death, so that interventions can be designed and evaluated.

One limitation of the GEMS and GEMS-1A studies is that although rotavirus was documented as the predominant pathogen responsible for MSD and LSD morbidity, because enrolment was done at sentinel health centres in which effective rehydration therapy was available to replace water and electrolyte deficits, deaths from rotavirus dehydration were uncommon. However, without access to health care, many infants or toddlers with rotavirus diarrhoeal dehydration would surely have died. Rotavirus aside, for groups estimating global diarrhoeal disease mortality by aetiology,^8^ the data from the GEMS and GEMS-1A studies reported here allow for improved disaggregation by severity, and direct estimation of population-based (*vs* hospital-based) risk of death due to MSD and LSD.^8^

The risk of death analyses in this study can help public health decision makers choose what investments to make among competing priorities. Our data emphasise the value of preventing or treating all diarrhoeal syndromes that bring children to health care, including LSD and MSD. Because the major attributable enteric pathogens associated with LSD and MSD are the same,^12^, ^16^ a public health argument can be made to avert the cascade of adverse events that follow MSD and LSD by primary prevention of illnesses caused by certain major attributed pathogens. One action would be to facilitate future implementation of vaccines against *Shigella* spp and ETEC that are currently in clinical development. Because vaccines against *Cryptosporidium* spp remain a distant goal,^30^ alternative strategies must diminish the ravages of that pathogen. One strategy would be to develop affordable point-of-care diagnostics to detect children infected with *Cryptosporidium* spp and safe, effective therapeutics to treat their infections. Collectively, such interventions can help to assure that by 2030 diarrhoeal disease will no longer be a major cause of death in children younger than 5 years in any resource-scarce, impoverished setting.^7^
