# Supplementary material for: Diarrhoeal disease and subsequent risk of death in infants and children residing in low-income and middle-income countries: analysis of the GEMS case-control study and 12-month GEMS-1A follow-on study
Source: Lancet Glob Health. 2019 Dec 18;8(2):e204–14. doi: 10.1016/S2214-109X(19)30541-8 (PMC7025325; doi:10.1016/S2214-109X(19)30541-8)
Supplement: Supplementary appendix [file mmc1.pdf]

THE LANCET
Global Health

Supplementary appendix

This appendix formed part of the original submission and has been peer reviewed. We post it as supplied by the authors.

Supplement to: Levine MM, Nasrin D, Acácio S, et al. Diarrhoeal disease and subsequent risk of death in infants and children residing in low-income and middle-income countries: analysis of the GEMS case-control study and 12-month GEMS-1A follow-on study. *Lancet Glob Health* 2019; published online Dec 18. http://dx.doi.org/10.1016/S2214-109X(19)30541-8.

Diarrheal Disease in Infants and Young Children in Developing Countries
Clinical Protocol

Sites:

Overall Study Coordinating Center:
Center for Vaccine Development (CVD)
University of Maryland School of Medicine
Baltimore, Maryland 21201, USA

Participating Field Sites (7 sites total):
1. Centro de Investigaçao em Saude da Manhiça (CISM), Manhiça, MOZAMBIQUE
2. Medical Research Council (MRC), Basse, THE GAMBIA
3. CDC/Kenya Medical Research Institute (KEMRI) Research Station, Kisumu, KENYA
4. Centre pour le Développement des Vaccins du Mali (CVD-Mali), Bamako, MALI
5. National Institute of Cholera and Enteric Diseases (NICED), Kolkata, West Bengal, INDIA
6. International Center for Diarrheal Disease Research (ICDDR,B), Mirzapur, BANGLADESH
7. Aga Khan University, Karachi, PAKISTAN

Investigators:

Overall Principal Investigators:

Karen L. Kotloff, M.D.	CVD, Baltimore, MD, USA – Principal Investigator (PI)
James P. Nataro, M.D., Ph.D.	U.of VA Sch of Medicine, Charlottesville, VA, USA – Co-PI

Coordinating Center Epidemiologists:

Dilruba Nasrin, MBBS, Ph.D.	CVD, Baltimore, USA
Tamer Farag, Ph.D.	CVD, Baltimore, USA

Site Principal Investigators:

Debasish Saha, MBBS, MPH	MRC, Basse, THE GAMBIA
Pedro Alonso, M.D., Ph.D.	CISM, Manhiça, MOZAMBIQUE
Robert F. Breiman, M.D.	CDC/KEMRI, Kisumu, KENYA
Samba Sow, M.D., M.Sc.	CVD-Mali, Bamako, MALI
Dipika Sur, M.D.	NICED, Kolkata, INDIA
ASG Faruque, M.D.	ICDDR,B, Mirzapur, BANGLADESH
Anita K.M. Zaidi, M.D.,MBBS,MS,FAAP	AKU, Karachi, PAKISTAN

Site Co-Investigators:

Martin Antonio, Ph.D.	MRC, Basse, THE GAMBIA
Mitchell Adeyemi, M.D.	MRC, Basse, THE GAMBIA
Inacio Mandomando, B.S.	CISM, Manhiça, MOZAMBIQUE
Tacilta Nhampossa, M.D.	CISM, Manhiça, MOZAMBIQUE
Ciara O'Reilly, Ph.D.	CDC/KEMRI, Kisumu, KENYA
Richard Omore	CDC/KEMRI, Kisumu, KENYA
Boubou Tamboura, M.D.	CVD-Mali, Bamako, MALI
Milagritos Tapia, M.D.	CVD-Mali, Bamako, MALI
Byomkesh Manna, Ph.D.	NICED, Kolkata, INDIA
T. Ramamurthy, M.D.	NICED, Kolkata, INDIA
Anowar Hossain, M.D.	ICDDR,B, Mirzapur, BANGLADESH
Shams El Arifeen, M.D.	ICDDR,B, Mirzapur, BANGLADESH
Rumina Hasan, MBBS, PhD	AKU, Karachi, PAKISTAN
Zulfiqar A Bhutta, MBBS, PhD	AKU, Karachi, PAKISTAN

Coordinating Investigator:
Myron M. Levine, M.D., D.T.P.H. CVD, Baltimore, MD, USA

Study design:

1. Demographic characterization of study populations
2. One random sample Health Care Services Utilization and Attitudes Survey (HUAS) of households containing a child 0-59 months of age in the census area of each site followed by an abbreviated HUAS (HUAS-lite) survey 2-3 times per year
3. Case control study of the incidence and etiology of moderate-to-severe diarrhea (MSD)
4. Case control study of the incidence and etiology of less severe diarrhea (LSD)

Subjects: Total up to 147,380 subjects

1. **Health Care Services Utilization and Attitudes Survey (n=85,790)**:
   Randomly selected households containing children 0-59 months of age (1,140 children per site in an initial survey followed by ~1,140 children per site every 4-6 month for 2-4 years)

2. **Case/Control Study of Moderate to Severe Diarrhea (MSD) in 2007-2011 (n=30,520)**:
   Children ages 0-59 months distributed as follows: 660 cases of moderate-to-severe diarrhea and 660 community controls, enrolled in each of three age strata (0-11 months, 12-23 months, and 24-59 months) at each of seven sites over a 36-month period to achieve ~600 analyzable cases and ~600 analyzable controls per strata per site, plus ~ 200 cases and 200 controls at each site (equally divided among the 3 age strata) during a 4-month pilot phase.

3. **Follow-on Case/Control Study of Moderate to Severe Diarrhea (MSD) and Less Severe Diarrhea (LSD) in 2011-2012 (n=17,160)**:
   Children 0-59 months of age visiting the Sentinel Health Centers with diarrhea will be categorized as having either MSD or LSD and enrolled along with 1-3 matched community controls for each case, distributed as follows:
   - MSD: All seven sites will continue to enroll ~220 MSD cases and ~220 controls (1-3 controls per case) into each age stratum over a 12-month period, with the aim of enrolling 1,320 children at each site, or 9,240 total children.
   - LSD: Six sites (Kenya is not participating) will enroll ~220 cases of less severe diarrhea (LSD) and ~220 community controls into each of three age strata --to achieve ~200 analyzable cases and ~200 analyzable controls per stratum per site. This will take place simultaneously with the follow-on MSD study. The target enrollment is 1,320 children at each site, or 7,920 total children.

Duration: This 6-year project will consist of:

Health Care Services Utilization and Attitudes Survey (HUAS):

Subjects:	*HUAS:* each interview will last up to 2 hrs; *HUAS-lite* interview will last 5-15 min.
Total project:	Serial surveys will be done every 4-6 months for up to 4 years at each site

Case/Control Study (MSD):

Subjects:	Each child will participate for ~60 days (range 50 – 90 days)
Total project:	5 sites: ~39 months (enrollment for 3 years, follow-up 50-90 days). Kenya and Mozambique, ~ 51 months (enrollment will last 4 years, follow-up ~50-90 days)

Follow-on Case/Control Study (MSD and LSD):

Subjects:	Each child will participate for 60 days (range 50 – 90 days)
Total project:	~15 months (enrollment will last for 1 year, follow-up for up to 90 days)

Primary Aim:

To estimate the population-based burden, microbiologic etiology and adverse clinical consequences of moderate-to-severe diarrhea (MSD) and less severe diarrhea (LSD) among children 0-59 months of age in study sites in sub-Sahara Africa and South Asia to guide the development and implementation of vaccines and other interventions.

Major Activities Designed to Achieve the Primary Aim:

1. Perform study initiation activities.
2. Perform demographic surveillance and assess health care utilization patterns at each field site.
3. Perform a case-control study to identify the etiology and quantify sequelae consequent to MSD and LSD among children 0-59 months of age at each field site.
4. Perform in-process quality assurance (QA), monitoring, training, and cross-site communication to ensure high quality data and protocol compliance.
5. Develop a "Vaccine Introduction Case" for vaccines against major indicated pathogens in Africa and Asia.
6. Create a Central Repository of well-characterized clinical specimens and isolated etiologic agents that can be accessed for further evaluation by approved investigators
7. Disseminate the results to the scientific community and health care authorities in each country involved.

Table of Contents

1.0 Background and Rationale 7
1.1.1. Common methodological deficiencies of current studies 7
1.1.2. Limitations in ability to use current data to estimate global disease burden 7
1.1.3. Future approaches 8
2.0 Goals, Objectives, and Major Activities 8
2.1.1. Goals 8
2.1.2. Objectives 9
2.1.3. Major activities 9
3.0 Study Sites 10
4.0 Census and demographic surveillance of catchment population at each site 11
4.1.1. Census and demographic surveillance system (DSS) 11
4.1.2. Detecting fatal cases of diarrheal illness not seen by health care facilities at the study site 12
5.0 Health Care Services Utilization and Attitudes Survey (HUAS) 12
5.1.1. Overview 12
5.1.2. Sampling frame 13
5.1.3. Contacting a selected child (initial HUAS) 13
5.1.4. HUAS questionnaire (initial HUAS) 14
5.1.5. Abbreviated HUAS questionnaire («HUAS-lite») 14
5.1.6. HUAS-lite sampling in Kenya (approved by UMB IRB under protocol modification # 2) 15
5.1.7. Data outcomes and analysis 15
5.1.8. Sample size considerations 16
6.0 Case-Control Burden and Etiology Study: Clinical Methods 17
6.1.1. Case Registration Form 17
6.1.2. Case Eligibility Form 18
6.1.3. Census ID Log 18
6.1.4. Case Enrollment 18
6.1.4.1. MSD Case Eligibility Criteria 19
6.1.4.2. LSD Case Eligibility Criteria 20
6.1.5. Control Enrollment 21
6.1.5.1. Control Eligibility Criteria 21
6.1.6. Informed consent 25
6.1.7. Clinical and epidemiologic data collection at enrollment 25
6.1.8. Memory aid to track duration of diarrhea 26
6.1.9. Clinical and epidemiologic data collection at follow-up 26
6.1.10. Water, Sanitation, and Hygiene Sub-studies (Mali, Pakistan and Gambia only, already approved by UMB IRB under protocol modification #7 and by relevant local IRBs) 26
6.1.11. HIV Substudies 26
6.1.11.1. Mozambique Counseling and Testing Substudy (already approved by UMB IRB under protocol modification #6 and by relevant local IRBs) 26
6.1.11.2. Kenya HIV Data Abstraction from the Demographic Surveillance System (approved by UMB under protocol modification #8 and by relevant local IRBs) 27
6.1.12. Verbal autopsy in the event that a child dies within 60 days of enrollment 27
6.1.13. Measuring height and weight 27
6.1.14. Specimen collection 27
6.1.15. Sample size considerations 29
7.0 Case-Control Burden and Etiology Study: Laboratory Methods 31
7.1.1. Specimen accession 31
7.1.2. Specimen processing for bacterial pathogen detection 31
7.1.3. Specimen processing for *E. coli* 32
7.1.4. Detection of protozoal agents 32
7.1.5. Detection of viral agents 32

7.1.6. Environmental Microbiology Isolation and Quantification of pathogens (Mali, Pakistan and Gambia only, already approved by UMB IRB under protocol modification #7) 33
7.1.7. Central Repository for stool specimens and bacterial isolates, and specimens containing viral and protozoal pathogens 33
7.1.8. Ethical considerations in testing stored specimens at collaborating centers 33
8.0 Data Management 34
8.1.1. Overview and description of the coordinating center 34
8.1.2. Data entry and transmission 34
8.1.3. Case report forms (CRFs) 35
8.1.4. Quality control 35
8.1.5. Site-specific databases 36
8.1.6. DCC Security 36
8.1.7. DCC Backup 36
9.0 Analytic Methods 37
9.1.1. Definitions 37
9.1.2. Endpoints 38
9.1.3. Calculation of primary endpoints 40
9.1.4. Calculation of secondary endpoints 44
9.4.1 Risk factors for MSD 44
9.4.2 Risk factors for less severe diarrhea 44
9.4.3 Economic analyses and the introduction case for selected vaccines in study countries 44
9.1.5. Expanded Risk Factor Studies and Environmental Microbiologic Studies 45
9.1.6. Statistical comparisons 45
10.0 In-process Quality Assurance (QA) 45
11.0 Monitoring Plan 46
11.1.1. Clinical and epidemiological activities 46
11.1.2. Microbiological activities 47
12.0 Risks and Benefits 47
13.0 References 47

List of Abbreviations

CDC	Centers for Disease Control and Prevention (Atlanta, Georgia, USA)
CFA	Colonization Factor Antigen of Enterotoxigenic *Escherichia coli*
CIN	Cefsulodin Irgasan Novobiocin agar
CSPCC	Cooperative Studies Program Coordinating Center
CISM	Centro de InvestigaΗao em Saude da Manhiça (Manhiça, Mozambique)
CRF	Case Report Form
CVD	Center for Vaccine Development (Baltimore, Maryland, USA)
CVD-Chile	Centro para Vacunas en Desarrollo (Santiago, Chile)
CVD-Mali	Centre pour le Développement des Vaccins du Mali (Bamako, Mali)
DAEC	Diffusely adherent *Escherichia coli*
DCC	Data Coordinating Center
DNA	Deoxyribonucleic Acid
DSS	Demographic Surveillance System
EAggEC	Enteroaggregative *E. coli*
EHEC	Enterohemorrhagic *E. coli*
EIA	Enzyme Immunoassay
EIEC	Enteroinvasive *E. coli*
EPEC	Enteropathogenic *E. coli*
ETEC	Enterotoxigenic *E. coli*
GCP	Good Clinical Practice
HUAS	Health Care Utilization and Attitudes Survey
ICDDR,B	International Center for Diarrheal Disease Research (Mirzapur, Bangladesh)
IRB	Institutional Review Board
KEMRI	CDC/Kenya Medical Research Institute (Kisumu, Kenya)
LSD	Less severe diarrhea
MDM	Molecular Diagnostics and Microbiology Section of the CVD
MRC	Medical Research Council (Basse, The Gambia)
MOP	Manual of Procedures
MSD	Moderate-to-severe diarrhea
N	Number (typically refers to subjects)
NCCLS	National Committee for Clinical Laboratory Standards
NICED	National Institute of Cholera and Enteric Diseases, Kolkata, India
PCR	Polymerase Chain Reaction
RNA	Ribonucleic Acid
RT-PCR	Reverse Transcriptase-Polymerase Chain Reaction
PI	Principal Investigator
PT	Proficiency Test
SD	Standard Deviation
SOP	Standard Operating Procedure
S-S	*Salmonella-Shigella* Agar
ST	Heat stable toxin of ETEC
TCBS	Thiosulfate citrate bile salts sucrose agar
TSI	Triple sugar iron agar
UMB	University of Maryland, Baltimore
QA	Quality Assurance
QC	Quality Control
WHO	World Health Organization
XLD	Xylose-Lysine-Desoxycholate Agar

6

1.0 Background and Rationale

1.1.1. Common methodological deficiencies of current studies

The World Health Organization (WHO) ranks diarrheal disease as the second most common cause of mortality among children under five years (60 months) of age in developing countries, accounting for 18% of the 10.6 million children in this age group who die each year.(1;2) Many studies have been conducted in various geographic sites to identify the etiology of these diarrheal illnesses and to formulate a composite picture for estimating their global burden. With few exceptions, however, available data suffer from notable deficiencies. For one, appropriate epidemiologic methods are seldom applied to allow population inference. To calculate incidence, various assumptions then must be made about the number of residents in the catchment area and the proportion of cases detected by surveillance. The resultant estimates can be spurious, particularly in regions such as sub-Saharan Africa that lack vital registration and ready access to hospital care.(3;4) Second, most study designs are cross-sectional to maximize economies of time and cost. However, cross-sectional studies do not capture the sequelae (adverse clinical consequences) of diarrhea that are so important to health outcome, namely persistent diarrhea, nutritional faltering, cognitive deficiencies, and death. Third, many studies do not distinguish the relative contribution of the major diarrheal disease clinical syndromes (acute gastroenteritis, profuse watery diarrhea, dysentery, and persistent diarrhea) which require discrete management strategies and lead to divergent outcomes. Fourth, the contribution of various bacterial, viral, and parasitic agents that can cause diarrhea has not been fully elucidated because many studies seek to identify a narrow scope of etiologic agents (often those of particular interest to the principal investigators), do not utilize standardized methods, and/or lack serological and/or antigenic typing of the organisms identified. Fifth, failure to look for etiologic agents in non-diarrheal matched controls in most studies means that the relative pathogenicity of the agent in that population cannot be determined. This omission is particularly relevant in highly endemic areas where many children shed enteropathogens asymptomatically. As a result, the risk of illness attributed to that pathogen may be misrepresented. Finally, few studies measure the financial cost of a child's diarrheal illness to the family and to the health care system to derive a comprehensive picture of the burden of disease that includes the economic burden.

1.1.2. Limitations in ability to use current data to estimate global disease burden

When existing data are analyzed to formulate a composite picture of the global burden of diarrheal disease, one remarkable deficiency is the paucity of systematic studies in the countries that bear the highest childhood mortality rates, most of which reside in sub-Saharan Africa.(5) As a consequence, global disease burden estimates must be mathematically modeled(6) or based on aggregated studies(7) and are substituted for direct measurement as a basis for priority-setting and resource allocation. These estimates are subject to considerable uncertainty when they include regions from which data are sparse, such as sub-Saharan Africa. In fact, some researchers have cautioned against using such global disease burden estimates until the assumptions can be validated with high quality data.(8)

One must be circumspect about using techniques that generalize diarrheal disease burden estimates from one region to another because the risk of diarrheal disease and death from diarrhea is influenced by many factors that can differ geographically. Among these are conditions intrinsic to the host (e.g., age, nutritional and micronutrient status, blood group, breastfeeding, exposure intensity, recent measles illness, and immunocompetence), the environment (e.g., availability of health care, safe water, sanitation, and hygiene), and the agent (e.g., its pathogenicity, transmissibility, and amenability to prevention and treatment). Local and

regional trends in vaccine coverage, economic development, political stability, environmental conditions, and emerging pathogens that cause severe diarrhea (such as *Shigella dysenteriae* type 1) or alter the host's susceptibility to severe diarrhea (such as HIV and measles) all contribute to this dynamic process, sometimes in an unpredictable and uneven fashion.

The patterns of all-cause under-5 mortality rates published by the WHO illustrate the importance of local trends.(1) Under-5 mortality rates fell worldwide throughout the latter part of the 20th century from 146 per 1000 in 1970 to 79 per 1000 in 2003. Since 1990, this rate has dropped by about 15%, equaling more than two million lives spared in 2003 alone. While these data are encouraging, a focus only on global patterns hides the following important regional differences. Whereas South-East Asia and the Americas have experienced rate reductions of 50% or more, the African Region, which began at the highest levels, has shown little improvement.(1) The factors impeding progress in child health in Africa are not clearly defined, particularly when it comes to diarrheal disease. Trends in age-specific diarrheal mortality provide an example of the vagaries specific to diarrheal disease epidemiology. Infants younger than 1 year, while continuing to experience the highest rates of diarrheal mortality, have enjoyed more marked reductions during the past 5 decades (~67%) than have children 1-4 years of age (~20%).(9) One could postulate that this pattern reflects recent changes in the relative burden of different enteropathogens attributable to the widespread use of oral rehydration therapy. Oral rehydration therapy impacts the outcome of agents causing acute dehydrating diarrhea in infants, such as rotavirus and enterotoxigenic *Escherichia coli* (ETEC), far more than the outcome of invasive agents such as *Shigella*, which peak in incidence among 1-4 year old children.(7) However, additional studies are required to better define the etiology of these age-specific trends so that appropriate interventions can be designed and implemented.

1.1.3. Future approaches

Recent advances in biotechnology have enabled promising new approaches to the construction of enteric vaccines. To set priorities for enteric vaccine development, design vaccines for broad coverage, guide public health policy, and target appropriate interventions, there is a compelling need to determine the etiology, burden, and sequelae of diarrheal diseases from multiple, heterogeneous geographic and epidemiologic settings. These data must be produced using methods that address deficiencies in previous studies, and must satisfy current needs for strategies that can diminish morbidity and mortality from diarrheal diseases, with a clear emphasis on children living in regions where mortality is high, such as in sub-Saharan Africa.

2.0 Goals, Objectives, and Major Activities

2.1.1. Goals

Under this protocol, we have conducted a 3-year, multi-center, case-control study to help quantify the burden, microbiologic etiology, and sequelae of MSD which we have termed Global Enterics Multicenter Study (GEMS) 1. Beginning in 2011, we propose to continue the multi-center, case-control study for an additional 12 months, to help quantify the burden, microbiologic etiology, and sequelae of diarrheal diseases that result in hospitalizations or visits to ambulatory urgent care facilities among children 0-59 months of age living in developing countries throughout Africa and Asia. We have termed this add-on study "GEMS 1a". Thus in addition to continuing the study of MSD for 12 more months, we will include a parallel case control study of less severe diarrhea (LSD), comprising the children seeking care at the health facilities who do not meet criteria for MSD but nonetheless have an illness that is sufficiently severe to prompt their parents or caretakers to bring them for medical care.

The sites selected for this study have moderate to high childhood mortality but are heterogeneous with respect to other health indicators to create a broad view of enteric disease epidemiology. The hospital and urgent care facility venues have been chosen to capture those illnesses that are most severe and that collectively constitute a significant cost in health care services, and thus would be targeted for prevention by vaccines. These data will be generated using a common research protocol of rigorous epidemiologic and microbiologic design to address limitations of previous studies, and to satisfy contemporary needs for information. By performing a case-control study to characterize the clinical, epidemiological, and microbiological features of diarrheal episodes in young children who receive care in communities whose demography and health care utilization practices are well described, we will be able to make reliable estimates of the burden of diarrheal diseases (incidence, sequelae, and economic costs by age and by pathogen) in that community. The **ultimate goal** is to provide information needed to guide the development and implementation of enteric vaccines and other public health interventions that can diminish morbidity and mortality from diarrheal diseases.

2.1.2. Objectives

Primary Objective

To estimate the population-based burden, microbiologic etiology and adverse clinical consequences of two types of diarrheal disease among children 0-59 months of age prompting a visit to a health care center at a study site in sub-Sahara Africa or South Asia: 1) moderate-to-severe diarrhea (MSD); and 2) less severe diarrhea (LSD). These data will be used to guide the development and implementation of vaccines and other interventions.

Secondary Objective

To assess, in a preliminary fashion, the relationship between HIV and morbidity and mortality related to moderate and severe diarrhea among children 0-59 months of age living in the setting of high HIV prevalence.

2.1.3. Major activities

The following major activities will be conducted to achieve the goals and aims of this clinical protocol:

To achieve the above OBJECTIVE, the following ACTIVITIES will have to be performed:

1. Perform study initiation activities.
2. Perform demographic surveillance and assess health care utilization patterns for diarrhea at each field site.
3. Initiate epidemiologic surveillance to estimate the incidence of MSD and LSD disease and perform a case-control study to identify the etiology and quantify sequelae consequent to MSD and LSD diarrhea among children 0-59 months of age at each field site.
4. Assess the relationship between HIV infection and the etiology and severity of diarrhea on two high HIV prevalence sites, Kenya and Mozambique.
5. Perform in-process quality assurance (QA), monitoring, training, and cross-site communication to ensure high quality data and protocol compliance.
6. Estimate public and private financial costs of diarrheal disease due to major indicated pathogens in Africa and Asia.
7. Create a Central Repository of well-characterized clinical specimens and isolated etiologic agents that can be accessed for further evaluation by approved investigators.
8. Disseminate the results.

3.0 Study Sites

Study sites were selected in developing countries in Asia and Africa with the principal goal of elucidating the etiology and epidemiology of diarrhea in regions with high childhood mortality rates, particularly in sub-Saharan Africa (**Figure 1**). Sites exemplify a spectrum of child health indicators, including high ($\geq$100) and medium (12-99) under-5 mortality per 1,000 live births, as well as high and low prevalence of malaria, rate of under-5 malaria deaths, and adult human immunodeficiency virus (HIV) prevalence (**Table 1**). While capacity-building is an intended benefit, sites must have pre-existing infrastructure including laboratory capabilities to perform coprocultures, antigen-detection and nucleic-acid based assays, and expertise in enteric microbiology and epidemiology. The population served by each site must be sufficiently large to provide the required number of MSD and LSD diarrhea cases in each age stratum.

Figure 1. Geographic distributions of sites

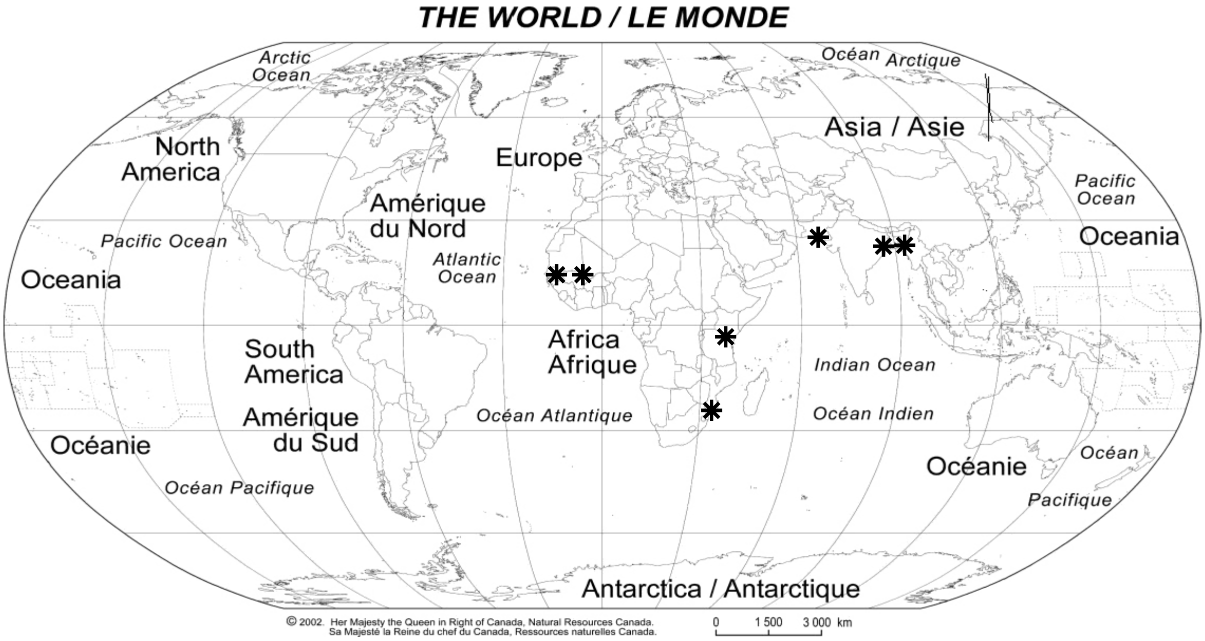

Table 1. Selected child health indicators in countries where sites were chosen

Country	Location	Partner	Venue	Nat'l U5MR*		Nat'l % 15-49 yr old HIV+ ¶	Nat'l Malaria Incidence (Mortality rank)§
				Value	Rank		
**Sub-Saharan Africa**							
Mali	Bamako	Centre pour le Developpement des Vaccins du Mali (CVD-Mali)	Urban	220	7	1.9	65.9 (1)
The Gambia	Basse	Medical Research Council (MRC)	Rural	123	37	1.2	ND (38)
Mozambique	Manhiça	Centro de InvestigaHao em Saude da Manhiça (CISM)	Rural	158	24	12.2	262.2 (7)
Kenya	Kisumu	CDC/Kenya Medical Research Institute (KEMRI) Research Station	Rural	123	37	6.7	3.7 (37)
**Asia**							
India	Kolkata, W. Bengal	National Institute of Cholera and Enteric Diseases (NICED)	Urban	87	54	0.9	1.6 (53)
Bangladesh	Mirzapur	International Center for Diarrheal Disease Research, Bangladesh (ICDDR,B)	Rural	69	62	<0.2	0.4 (58)
Pakistan	Karachi	Aga Khan University	Peri-urban	103	47	<0.2	0.8 (50)

ND = no data; MR = mortality rate

* UMR=under 5 mortality; value is calculated per 1,000 live births and ranked out of 192 countries(10)
¶ Prevalence of HIV (percentage) among 15 to 49 year olds, as of end 2003. [Source: http://www.who.int/GlobalAtlas/predefinedReports/EFS2004/index.asp, accessed 8/19/05]
§ Standardized reported malaria rate per 1,000 population, 2003 [Source: WHO World Malaria Report 2005 http://rbm.who.int/wmr2005/html/a4.htm, accessed 8/19/05]; (2000 global ranking of under-5 malaria-related mortality rate per 100,000 olds (1-59) per United Nations Common Database shown in parenthesis [source: http://atlas.globalhealth.org/indicator.cfm, accessed 8/26/05]).

4.0 Census and demographic surveillance of catchment population at each site

4.1.1. Census and demographic surveillance system (DSS)

The population at each field site will be required to have a baseline (or very recent) census and will have to commit to maintain (throughout the duration of the study) demographic surveillance consisting of a visit to every household once every four to six months to record births, deaths and migrations in and out of the community. Depending on the site, surveillance to detect and enroll age-eligible cases of MSD and LSD diarrheal illness into the study will be maintained at a variable number of health care facilities, among all the facilities that serve the population under demographic surveillance. **The census and DSS will be conducted either as part of a governmental activity not requiring IRB approval or under a separate protocol that will be reviewed by both the local IRB and the University of Maryland IRB.**

Table 2. Salient features of the 7 study sites

Site	Location	Total DSS pop'n (No. 0-59 mo.)	Sample of Health Centers (HC) serving catchment pop'n	Sample of HCs where case-control study enrollment will ensue	Zinc *
Manhica, Mozambique	Manhica District, Maputo Province. Covers 500 km^2	84,206 (16,657)	Manhiça HC; Health post & maternity at: Maragra; Ilha Josina; Taninga; Palmeira, & Maluana	Same excluding Maluana	No
Kisumu, Kenya	217 villages along N shores of Lake Victoria in Nyanza Province, ~ 40km from Kisumu City.	141,628 (23,294)	Two district hospitals (Bondo and Siaya) and ~13 Clinics	Selected health facilities from the list below; 5 outpt HCs): Ongielo, Abidha, Akala, Njera, Rera; 1 HC (inpts & outpts): Lwak in Asembo and Gem, and Siaya District Hospital (inpts & outpts), 4 HCs (Ting Wangi, Ngiya Mission, Nyathengo, Bar Agulu, and Bar Olengo), and 3 dispensaries (Kogelo and Mulaha) in Karemo.	No
Basse, The Gambia	Upper River Division	157,726 (28,898)	Basse, Gambisara, Fatoto, Garawol, Demba Kunda, Koina	Same	No
Bamako, Mali	Djikoroni (western Bamako) & Banconi (northern Bamako) quartiers.	210,425 (32,526)	Hôpital Gabriel Touré (HGT, sole pediatric tertiary HC). Djikoroni-para: 2 community HCs (CSCOMs) serve >90% of pop'n; 3 private HC.Severe cases referred to Commune IV Reference (CSRef IV) HC. Banconi: 1 CSCOM, refers to CSRef I HC	HGT, CSRef I and IV, CSCOMs in Banconi, Djikoroni Para, Djene-kabougou, and Bodjanbougou, Camp Para Infirmary, Sadja & Cherifla Private HC	No
Kolkata, India	Municipal wards 14, 31, 34, 58, 59.	194,712 (12,885)	Infectious Dis. Hospital (IDH); Municipal BC Roy Children's Hospital; private practitioners	IDH; BC Roy Children's Hospital, heath outposts in Ward 58 & 59	Partial
Mirzapur,	Tangail subdistrict,	254,751	Kumudini Hospital, Jamurki Sadar	Kumudini Hospital	Yes

Bangladesh	1.5h. NW of Dhaka	(24,077)	Hospital		(since 2007)
Karachi, Pakistan	4 peri-urban coastal communities	252,346 (24,792)	Primary care centers, Sindh Gov't hospital, other pvt HC	Ibrahim Hyderi, Rehri Goth, Ali Akbar Shah, Nat'l Instit. of Children Hosp., Ma Ayesha HC, Bhain's Colony HC, Sindh Gov't Hosp.	Partial

*Zinc supplementation currently given as part of routine pediatric primary care. DSS=Demographic Surveillance Survey

4.1.2. Detecting fatal cases of diarrheal illness not seen by health care facilities at the study site

The 60-day follow-up visit described below will permit quantification of diarrheal deaths among children in the catchment population who visit the surveillance health centers and are enrolled in the case-control study. Ongoing demographic surveillance (11-14) at each field site and the use of verbal autopsies will allow an estimate of the proportion of deaths among children 0-59 months of age who died outside the site's health care facilities that were likely related to diarrheal disease at each field site and prudent use of verbal autopsies will allow an estimate of the proportion of deaths among children 0-59 months of age who died outside the site's surveillance health care facilities that were likely diarrheal disease deaths.(15-18). Whereas the proportion of pediatric deaths that occur outside of hospitals and health centers may not be high in urban sites, it may be substantial in rural sites. The investigators recognize the uncertainties and limitations in the use of verbal autopsies to determine the probable cause of death in children from developing countries where a reliable system of registration is not available. The methods will be optimized by using standardized methods and systematic coding across sites and ensuring that field staffs are highly trained.

5.0 Health Care Services Utilization and Attitudes Survey (HUAS)

5.1.1. Overview

Each site will conduct one survey of health care services utilization and attitudes at the beginning of the study.(19-21) Thereafter, an abbreviated survey will be conducted with each round of DSS (designated "HUAS-lite"). These surveys will provide data on where parents seek care when their children have diarrheal disease (whether MSD or not), and their attitudes and practices concerning diarrhea, its prevention and treatment. This information will be used to optimize the surveillance for determining disease burden, to perform data adjustments to account for the proportion of children with diarrhea who do not seek care at the health care facilities participating in surveillance, to calculate population-based incidence rates, to compare data between the different sites, and to assess public perception of the need for interventions to prevent childhood diarrhea. Ideally 100% of patients with diarrhea will receive care at the hospital(s) or urgent care setting(s) participating in the study at the field site where cases of diarrhea are counted and analyzed, designated henceforth as the "**sentinel health centers**". Because the catchment population at most of the sites is quite large and there are typically multiple sources of health care, it may be logistically impractical to maintain both surveillance for diarrhea and the ability to enroll patients into the case-control study at all the health care facilities that serve the population. Moreover, at each site some fraction of children will not reach a health care facility when they have diarrhea. If the HUAS indicates that the point estimate of patients with MSD treated at the sentinel health centers falls below 75%, measures will be taken to try and increase this percentage by including additional health care facilities in the surveillance.

5.1.2. Sampling frame

Many of the HUAS methods are adapted from the *Generic protocol for a community-based survey on utilization of health care services for gastroenteritis in children under 5 years of age*.(19) One important difference is that since the catchment population at each site will have a recent census and ongoing demographic surveillance, it will be possible to randomly select children 0-59 months of age to participate in the HUAS. From the census at each study site, updated by the ongoing surveillance, computerized lists of children will be constructed for each of the age groups of interest (0-11, 12-23, and 24-59 months). The census database thus should be as current as possible, in order to include recent births and to put children who have crossed an age group boundary in the appropriate group. The list will define the population from which the HUAS sample is drawn. Even with an updated list, there will be births between the time of preparation of the list and conduct of the survey, so that infants at the earliest ages will probably be somewhat underrepresented in the HUAS.

For each survey (the initial as well as each follow-up survey), each of the sites (except for Kenya in the HUAS-lite survey, as described in section 5.6 below) will select a random selection of approximately 400 eligible from the 0-11 month age group (over-sampled because of the potential difficulties locating children in this age group, e.g., because of aging beyond the strata between DSS rounds, as yet undetected births which occurred between DSS rounds, and higher mortality rates), and 370 eligible children from each of the two older age strata (12-23 months and 24-59 months) using the updated census list. It is desired to include at least 333 children in each age group in the survey. The purpose of starting out with a larger sample is to allow for children who are on the list but are not actually eligible for the HUAS. Possible reasons for ineligibility include aging out of the age group for which the list is prepared, some of which will occur in spite of updating the list; a child no longer living or miscoded as living in the HUAS area; death of a child; and errors in the census and/or surveillance.

When the HUAS interviewers identify an ineligible child in the HUAS sample, that child will be deleted from the sample and the census/surveillance list for that age group, in order to make the list as accurate as possible. Thus, the final sample for each age group may be somewhat less than 400 for the youngest strata and 370 for the older strata, but unless non-response rates are higher than 20% and 11%, respectively, at least 333. **If the interviewer finds that a child falls outside of his/her assigned age strata but is nonetheless <60 months of age, the interview will be performed. If the child is ≥60 months old, s/he will be considered ineligible and the interview will not be conducted.**

If information for a child in the HUAS sample cannot be obtained after three attempts by the interviewer, but the child is considered eligible according to age and location of residence, that child will be kept in the sample and considered a non-responder. If the child is eligible but the primary caretaker refuses to participate, the child will be kept in the sample and considered a refusal. The analysis will adjust for non-response and refusal in the weights that are assigned to each child for whom information is obtained using the DSS sample as a whole. To perform this weighted analysis, the site will save the DSS dataset each time a survey is performed, They will record the number of children in the DSS population from which the HUAS sample was chosen who belong to each age strata, by gender. These data will be sent to the DCC for use during analysis to weight the sample according to the DSS population.

5.1.3. Contacting a selected child (initial HUAS)

The interviewer will go to the child’s home and describe the study to the child’s parent or primary caretaker. If the parent/primary caretaker wishes to participate, the consent form will be

13

read aloud by the interviewer in the local language and the parent/primary caretaker will be given an opportunity to ask questions. Thereafter, s/he will either provide a signature or (if s/he does not know how to write) place a mark on the consent form (this could be an "x" or a fingerprint) in the presence of a witness (who will also sign the form) indicating his/her willingness to participate. The parent/primary caretaker will receive a copy of the signed consent form to keep and the original will be stored in the regulatory files at the study site. The respondent will be the child's primary caretaker. If a primary caretaker is not available, this will be recorded and the interviewer will try to leave a message indicating when the interviewer is likely to return. A total of 3 attempts will be made to contact a primary caretaker after which time the child will be considered a nonresponder.

5.1.4. HUAS questionnaire (initial HUAS)

A new questionnaire will be used for each child contacted. The data collected from the ~60 questions asked include information about the household and family composition, occurrence of recent diarrheal illnesses among children younger than 5 years, and health care utilization practices. The HUAS questionnaire also provides an opportunity to query a representative sample of the population on their attitudes concerning diarrhea, its prevention and treatment. The survey may be modified as appropriate for each site, while adhering to the main elements. In some countries, birth date may not be known and it may be necessary to use an events calendar to estimate ages. The first page of the questionnaire will contain identifying information; this page will be kept locally at each site; the remaining pages of the form containing the study number and information obtained will be transmitted to the central data center.

The effect of distance from the health center as well as topologic barriers on health care utilization will be analyzed. Therefore, the location of the household within the census tract will be of interest. To collect this information, a log will be maintained linking the child's HUAS study identification number with data from the census such as the child's census ID number, village name, census cluster number, compound number, household number, and global positioning system (GPS) coordinates. Although no names will be recorded, this information could potentially be linked to a child's identity. Therefore, the following precautions will be taken to maintain confidentiality: the log will be transmitted to the data coordinating center via email separate from the clinical and epidemiologic information that is collected and will be stored at the central data coordinating center in a secure location, separate from the case report forms.

5.1.5. Abbreviated HUAS questionnaire («HUAS-lite»)

The sites will perform the DSS according to their standard practice (not part of this protocol). The DSS contains site-specific demographic questions. Mozambique and Kenya already obtain morbidity information during the DSS and will not request additional consent for these questions. The remaining sites will obtain verbal consent for the HUAS-lite questionnaire for these reasons:

- The added questions are asked in the context of the DSS for which demographic questions are already being asked
- Results from the first HUAS suggest that 7-25% of 0-59 month old children (varying by site) experienced diarrhea in the previous 2 weeks; therefore, 75-93% of respondents will be asked only two questions ("are you the primary caretaker of the child" and "has [child] had an illness with diarrhea in the last 14 days"). If the child had diarrhea, then 13 additional questions will be asked.
- This is minimal risk
- The form is labeled only with the child's census ID number, although this could be linked to the child's identity, it would require considerable effort.

The suggested verbal consent reads as follows (but may be adapted by each site as appropriate):

"*We are conducting a research study to learn more about diarrheal illnesses that affect infants and children during the first 5 years of life. We would like to ask you some questions about the diarrheal illnesses that [Child's Name] may have had recently. The questions will take about 10 minutes or less. The information collected about your child will be shared with people in the U.S. who are helping with this project but it will not contain your child's name. We will keep this form in a locked file which only our staff have permission to access. You do not have to answer these questions, and you can stop participating at any time. Should you refuse to take part in the study, or decide to stop participating, you will continue to receive your usual medical care."*

Parent or caretaker gives verbal consent*:* ☐***No*** ☐***Yes***

5.1.6. HUAS-lite sampling in Kenya (approved by UMB IRB under protocol modification # 2)

In Kenya, the HUAS-lite questionnaire will be administered to all children in the DSS rather than to a random sample. This is because the HUAS-lite questionnaire must be appended to an existing DSS data collection software program on a handheld Personal Digital Assistants (PDAs). The existing PDA software does not allow for random sampling of participants and the management logistics necessary to select and record HUAS-lite data from a random sample would have been cost-prohibitive. As a result, in Kenya we will administer the HUAS-lite interview to the caretaker of all children in the DSS < 60 months of age (~20,965 children). The larger sample size will allow for greater measurement precision and population representativeness so the additional data will add value to the survey. IRB approval to perform the HUAS-lite for all children < 60 months of age in the Kenya DSS was previously approved both locally (under the local site DSS protocol) and at the UMB IRB.

5.1.7. Data outcomes and analysis

The questionnaire is designed to permit determination of the total number of children under 5 years of age, the number who met our criteria for MSD diarrhea and for LSD within the past 14 days, and the number who ultimately received care, within the first 7 days of illness, at one of the sentinel health centers. This information allows determination of the ability of the sentinel health centers at each study site to capture cases of MSD diarrhea within the 7-day eligibility period. The survey also makes it possible to estimate the true incidence of MSD diarrhea and LSD throughout the study period. A reported episode will be considered as "MSD diarrhea" if <u>any</u> of the following three criteria are present, while a reported episode of LSD will be considered if none of the following three are present:

1. At least one of the following indicating moderate-to-severe dehydration: sunken eyes, more than normal, loss of skin turgor, or intravenous rehydration administered
2. Dysentery (diarrhea with visible blood in stool)
3. Hospitalization with diarrhea or dysentery

If the child did not experience a diarrheal episode (MSD or LSD) during the past 14 days, then parents will be asked hypothetical questions about their anticipated health care utilization should their child develop such an illness. This will permit a determination of the proportion of mothers who *would* use the sentinel health centers if their children had an episode of diarrhea in the future. Information on the respondents' perceptions about the danger of diarrheal disease in children, their attitudes about the importance of developing vaccines and other interventions will also be collected.

The analysis will be performed as follows. A weight will be assigned to each responder – i.e., to each child in the sample for whom information is actually obtained. If the survey information is only partially complete for a child, that child will be considered a responder for the information obtained and a non-responder for the missing information. The weight assigned each

child will represent the number of children in the population represented by the child. Weights will be calculated by age group and possibly by categories defined by one or more other variables within the age group. Within each category, the sum of the weights for responders in the survey sample will be the total number in the population in that category. Thus, the weight for each responder will be the population total in that category divided by the number of HUAS responders in the category. In this way, weights will be adjusted within each category for non-response. For each age group, variables used in assigning weights will be categorical, either by definition or by forming appropriate categories for a continuous variable, so that they can be used in defining the age (and possibly other) categories. Such variables will be those that, within the age group, are found to be related both to the probability of responding to the HUAS and the probability that a survey child with MSD (or LSD) will be taken for care to a health facility associated with the study site. Such variables might be different at different sites. Possible variables of interest are gender, number of other children cared for by the caretaker, distance from a study health care facility, or presence of some factor that might be an obstacle to seeking care at a study health care facility. Association with the probability of seeking care at a study health care facility can be evaluated for any variable present in the HUAS data; assessment of the probability of responding to the HUAS requires that the variable also be present in the census/surveillance data. To be used in assigning weights, a variable must be present in both datasets. Associations of HUAS variables with the probability of seeking care at a study health care facility and with the probability of responding to the HUAS will be assessed using logistic regression models. In these models, seeking care or responding to the HUAS will be the dependent variable and the other variable(s) of interest (gender, etc.) will be independent variables.

Within each age group or smaller category defined by other variable(s) as described above, the population total with a characteristic X of interest is by definition the sum of the weights for HUAS responders in the age group or in the category within the age group. Let X and Y be estimated population totals for the number of children in the population with MSD (or LSD) and the number of those who receive care at a study health care facility within 7 days of onset of the diarrheal illness. Let R be the estimated proportion of children with MSD (or LSD) who receive care at a study health care facility; then R = Y / X.

In order to estimate a confidence interval (CI) around R, e.g., a 95% CI, a jackknife procedure will be used for estimating variance. In such a procedure, an appropriate number of unique subsamples (e.g., 200) will be drawn for each age group or smaller category. One way to form a subsample is by deleting one or more randomly chosen observations from the original sample, including non-responders in the original sample, and taking what is left as the subsample. For each subsample, weights will be calculated and R obtained as though the subsample were the entire original sample. The variance of R in the entire original sample will be estimated from the R's for the subsamples. The 95% CI for the population value of R will be R $\pm$ 1.96 s, where s is the square root of the variance estimated by jackknife as described above. This assumes that the ratio R is approximately normally distributed.

5.1.8. Sample size considerations

The primary endpoint of the survey at a participating site is the proportion of children 0-59 months of age with MSD (or LSD) during the previous two weeks who received care at a sentinel health center associated with the site within 7 days of the onset of the illness. The sample size should be sufficient for a 95% confidence interval (CI) for this proportion to be reasonably narrow. Let P denote the proportion of children 0-59 months of age with MSD (or LSD) who receive care at a site's sentinel health center(s). Of a total of N_T children 0-59 months of age (or in a particular age subgroup), let N represent the number in the survey sample. We will observe proportions p_0 of children who do not have a diarrhea episode, p_1 who have an episode of MSD (or LSD) and

receive care at the health care facility, and p_2 who have MSD (or LSD) but do not receive care at the health facility. Then $P = p_1 / (p_1 + p_2)$.

From the multinomial distribution and considerations of conditional probability, a large sample approximation for the variance of P can be obtained by a Taylor series expansion as $V = (1 - N/N_T)\ P\ (1 - P)\ W$, where $W = 1 / (Nt) + (1 - t) / (Nt)^2 + (1 - t)(2 - t) / (Nt)^3 + (1 - t)[1 - 6t(1 - t)] / (Nt)^4$ and $t = p_1 + p_2$.

For a subset as small as 100 in size and $p_1 + p_2 = 0.05$, the error in W should be less than approximately 0.001 in absolute value. The estimated standard error of P, s, is the square root of V. Assuming that for large samples P is approximately normally distributed, an approximate 95% CI for Π, the true proportion of children with MSD (or LSD) who receive care at a health center, is $P \pm 1.96$ s. Table 3 gives 95% CIs for Π, for N = 333 and 999; $p_1 + p_2 = 0.03\text{-}0.3$; and observed probabilities p_1 and p_2 so that P is 0.5 and 0.8. The finite population correction, $1 - N/N_T$, is not included in the calculations, since for large populations it will have only a trivial effect on the CI.

Table 3. Sample size considerations for HUAS

**1. P = 0.5**					**2. P = 0.8**				
**N**	**p_1+p_2**	**p_1**	**p_2**	**95% CI**	**N**	**p_1+p_2**	**p_1**	**p_2**	**95% CI**
333	0.03	0.015	0.015	(0.17, 0.83)	333	0.03	0.024	0.06	(0.54, 1)
	0.1	0.05	0.05	(0.33, 0.67)		0.1	0.08	0.02	(0.66, 0.94)
	0.2	0.10	0.10	(0.38, 0.62)		0.2	0.16	0.04	(0.70, 0.90)
	0.3	0.15	0.15	(0.40, 0.60)		0.3	0.24	0.06	(0.72, 0.88)
999	0.03	0.015	0.015	(0.32, 0.68)	999	0.03	0.024	0.06	(0.65, 0.95)
	0.1	0.05	0.05	(0.40, 0.60)		0.10	0.08	0.02	(0.72, 0.88)
	0.2	0.10	0.10	(0.43, 0.57)		0.20	0.16	0.04	(0.74, 0.86)
	0.3	0.15	0.15	(0.44, 0.56)		0.4	0.24	0.06	(0.75, 0.85)

For small numbers of cases, the CI will be estimated by exact methods. Appropriate weights will be used in estimates for the overall age group to account for different sampling fractions in the age subgroups and any non-response that might occur.

According to Table 3, a total sample size of 999 will give a confidence interval for P with half-width at most about 0.10-0.18 around the point estimate for observed P=0.5 or 0.8 and the total proportion of children with MSD (or LSD) during the preceding 2 weeks between 0.03 and 0.4. The sample will be increased by 20% in the youngest age group and 11% in the two older age strata to allow for children who are on the randomization list but are not actually eligible for the HUAS because of migration, etc. as discussed above, achieving a sample size of 1140.

6.0 Case-Control Burden and Etiology Study: Clinical Methods

6.1.1. Case Registration Form

At each field site, in every sentinel health care facility, a Registration Log will be maintained to record the total number of children younger than 60 months of age who seek medical care and the number who meet the case definition of diarrhea. The information recorded will include the date, time, sequentially assigned register number, child's age (in months with first month of life designated as zero), child's gender, child's residence (recorded in such a way as to indicate whether the child belongs to the study catchment area) and whether s/he belongs to the Demographic Surveillance System (DSS) population, hospitalization (yes/no), and diarrhea (whether the child passed three or more abnormally loose stools in the previous 24 hours).

6.1.2. Case Eligibility Form

For children who meet the definition of diarrhea (3 or more abnormally loose stools in the previous 24 hours), the following additional information will be recorded on a Case Eligibility Log: simple demographic data, duration of diarrhea (in calendar days), whether this is a new diarrhea episode (i.e., the child was diarrhea-free for at least 7 days before this episode), dysentery (yes/no), sunken eyes more than normal (yes/no), loss of skin turgor (yes/no), intravenous hydration administered (yes/no), and enrolled in the diarrheal disease case control study (yes/no). It will be recorded whether the child was enrolled, and the reason for non-enrollment.

6.1.3. Census ID Log

In the analysis, it is important to determine whether the same child is enrolled in the study repeatedly as either a case or a control. The census ID number is a unique number for each child in the census population that can be used for this purpose. Therefore, the site will maintain a log containing a link between data from the census and the patient ID number in the current study which they will periodically transmit to the Data Coordinating Center (DCC). The data from the census will include information such as the child's census ID number, village name, census cluster number, compound number, household number, and global positioning system (GPS) coordinates. Although no names will be recorded, this information could potentially be linked to a child's identity. Therefore, the following precautions will be taken to maintain confidentiality: the log will be sent to the data coordinating center via email separate from the clinical and epidemiologic information that is collected and will be stored at the central data coordinating center in a secure location, separate from the case report forms.

6.1.4. Case Enrollment

Note regarding the 12-month follow-on of the case-control study: The original submission described a 3 year case control study of MSD at 6 sites and a 2-year study in Pakistan. Subsequent funding was approved to continue the case-control study in Pakistan for the full 3 years. We now have additional funds to continue the case control study for another year (2011-12), during which time the sites will participate in the following ways (**Table 3a**):

Table 3a. Overview of Study Activities during 2011-2012, by Site

Site	HUAS-lite	Case-Control Study		HIV Substudy
		MSD	LSD	
India, Bangladesh, Gambia, Mali, Pakistan	Yes	Yes	Yes	No
Kenya	Yes	Yes	No	Yes
Mozambique	Yes	Yes	Yes	Yes

MSD Case Sampling Strategy (2007-2011): Children 0- 59 months of age with acute diarrhea who meet the inclusion criteria listed below for MSD were recruited into the study. During the first ~4 months in the first year, up to 200 cases with MSD were enrolled in a pilot phase. Thereafter, each site aimed to enroll approximately 220 MSD patients per year from each of the following three age strata: 0-11 months, 12-23 months, and 24-59 months. Recruitment into the MSD case-controls study lasted for 3 years at all study sites targeting a total of ~660 children in each age stratum (to achieve 600 analyzable cases per stratum).

Follow-on MSD and LSD Case Sampling Strategy: During the 12-month follow-on case-control study, all seven sites will continue the MSD case-control study. Concomitantly, all sites except for Kenya will initiate a LSD case control study. The aim is to enroll up to 220 cases with LSD and 220 cases of MSD into the three age strata. Kenya will participate only in the MSD study during this 12-month period.

To ensure even sampling throughout the year, the sites will each enroll approximately 8-9 cases per age stratum (25-26 cases overall) per fortnight throughout the enrollment period into the LSD and/or MSD study, as applicable. This strategy will prevent the strata from being filled prematurely in sites with high volume and will ensure even sampling of the age groups, while respecting the capacity limitations of the clinical and microbiology personnel. Importantly, this sampling method is logistically robust for all sites as it assures a smooth workflow, while allowing seasonal increases or epidemics of specific diarrheal pathogens to be detected and recorded reliably. When the microbiological analyses are completed, the proportion of cases that yield a specific pathogen will be known for each 2-week and 4-week period. In data analysis, this proportion will be applied to the total number of moderate-to-severe cases of diarrhea seen in that time period to estimate the total number of cases of that pathogen.

The following information that is tracked in the **Case Registration and Eligibility Logs** will allow the denominators to be adjusted to derive population-based estimates of MSD, LSD, and other study endpoints in each age strata (0-11 months, 12-23 months and 24-59 months):

a. Total number of visits to hospital or health center among children 0-59 months belonging to the DSS
b. Total number admitted (if the center has inpatient facilities).

The following will be tabulated for MSD and LSD separately:

a. Total number of admissions/visits with diarrhea eligible to enroll (meets inclusion criteria)
b. Total number of admissions/visits with diarrhea invited to enroll.
c. Total number of admissions/visits with diarrhea enrolled.
d. Total number of admissions/visits with dysentery eligible to enroll.
e. Total number of admissions/visits with dysentery invited to enroll.
f. Total number of admissions/visits with dysentery enrolled.

Children with diarrhea will be tabulated as follows:

i. Ineligible
ii. Eligible and enrolled
iii. Eligible and missed
    a) 14 day quota filled
    b) Child died before invitation
    c) Deemed too ill for research activities
    d) Caretaker not available
    e) Sufficient stool sample not provided ($\geq$5 g or the size of 5 peas, Kenya will remain at >4 g) within the allowable time period
iv. Eligible and refused
    a) Parent/caretaker too busy
    b) Do not like research
    c) Child too sick
v. Other, specify__

6.1.4.1. MSD Case Eligibility Criteria

<u>Exclusion criterion</u>

1. Currently enrolled as a case of MSD ("currently enrolled" means enrolled and pending 60-day follow-up visit)

Inclusion criteria

1. 0-59 months of age.
2. Resides in the local demographic surveillance system (DSS) catchment area
3. Seeking care at a sentinel health center belonging to the DSS
4. Diarrhea, defined as 3 or more abnormally loose stools during the previous 24 hours
5. The onset of the diarrheal episode is within 7 days of enrollment into the study and it represents a new episode, meaning that ≥7 days to have passed since the last occurrence of diarrhea (22;23)
6. The diarrhea must be "moderate-to-severe", meaning that the child must meet at least one of the following criteria:
   a. Sunken eyes, more than normal
   b. Loss of skin turgor
   c. Intravenous rehydration administered or prescribed
   d. Dysentery (diarrhea with visible blood in stool)
   e. Hospitalized with diarrhea or dysentery

A child must provide an adequate stool sample for microbiological analysis (≥5 g or the size of 5 peas, Kenya will remain at 4 g) during the required time period to be included in the analysis.

Note -- The following situations are **acceptable** for enrollment as long as the selection follows the site's sampling methods, and the other inclusion criteria are met:

- Current LSD case presents with MSD during the same episode (i.e., the LSD progresses to MSD without 7 diarrhea-free days in the interim)
- Current LSD case presents with a new episode of MSD (i.e., after 7 or more days diarrhea-free)
- Current MSD or LSD control, presents with MSD at any time
- Previous MSD or LSD case, 60-day follow-up is completed, and child presents with a new episode of MSD

6.1.4.2. LSD Case Eligibility Criteria

Exclusion criteria

1. Currently enrolled as a case of LSD (i.e., "currently enrolled" means enrolled and pending 60-day follow-up visit)
2. Currently enrolled as MSD case for an episode that is ongoing (i.e., without 7 diarrhea-free days since the onset)

Inclusion criteria

1. 0-59 months of age.
2. Resides in the local demographic surveillance system (DSS) catchment area
3. Seeking care at a sentinel health center belonging to the DSS.
4. Diarrhea, defined as 3 or more abnormally loose stools during the previous 24 hours.
5. The onset of the diarrheal episode is within 7 days of enrollment into the study and it represents a new episode, meaning that ≥7 days to have passed since the last occurrence of diarrhea.(22;23)
6. None of the following are present:
   a. Sunken eyes, more than normal
   b. Loss of skin turgor
   c. Intravenous rehydration administered or prescribed
   d. Dysentery (diarrhea with visible blood in stool)
   e. Hospitalized with diarrhea or dysentery

20

Note -- The following situations are **acceptable** for enrollment as long as the selection follows the site's sampling methods, and the other inclusion criteria are met:

- Current MSD case presents with a new episode of LSD (i.e., after 7 days diarrhea-free) before the 60-day follow-up visit for the MSD episode is made
- Current MSD or LSD control, presents with LSD at any time
- Previous MSD or LSD case whose 60-day follow-up is completed, and child presents with a new episode of LSD

A child must provide an adequate stool sample for microbiological analysis ($\geq$5 g or the size of 5 peas, Kenya will remain at 4 g) during the required time period to be included in the analysis.

These criteria are summarized in **Table 3b.**

Table 3b. Enrollment criteria for GEMS 1a for previously enrolled children

	**Eligibility (If 7 days diarrhea free but 60-day follow-up not completed)**			
**Current status**	**LSD Case**	**MSD Case**	**LSD Control**	**MSD Control**
**LSD Case**	No	YES*	YES	YES
**MSD Case**	YES	No	No	No
**LSD Control**	YES	YES	No	No
**MSD Control**	YES	YES	No	No

*If an LSD case progresses to MSD during the first 7 days of illness, s/he may be enrolled as an MSD case as long as the selection follows the site's sampling methods, and the other inclusion criteria are met.

6.1.5. Control Enrollment

Control Sampling strategy. For each child with diarrhea enrolled in either the MSD or the LSD case control study, at least one healthy control child will be randomly selected from the community or village in which the case resides. **Thus, at least 8 control children per age stratum will be enrolled per fortnight at each site throughout the 3-year enrollment period.** The control will be matched to the case by age, gender, and time that the index case presented. A list of a minimum of four potential controls will be identified by computer from the demographic surveillance database. A field worker will first visit potential controls until a control is identified who is eligible, agrees to participate, and is able to provide an adequate (at least 5 grams) whole stool in a timely fashion. The **Memory Aid** described below will be used to determine whether a control goes on to develop diarrhea within 7 days of presentation of the index case. If the memory aid data prove to be robust, then controls who develop diarrhea within 7 days of the index case will be excluded from analysis and will not be replaced. The strategy for age matching controls to cases is shown in **Table 4**.

In order to adjust for bias in control selection, the following information will be tracked for each matched control using a **Control Registration Log**:

1. Total number of controls identified by computer
2. Total number of households approached
3. Total number of eligible controls
4. Total number of controls invited to participate

6.1.5.1. Control Eligibility Criteria

Inclusion criteria:

1. Age-matched to index case as follows: ±2 months for cases 0-11 months, and ±4 months for cases 12-59 months. The matched control may not exceed the stratum boundaries of the case, i.e., a control for an 11 month old case must be between the ages of 9 and 11 months and a control for a 13 month old must be between the ages of 12 and 17 months (**Table 4**)
2. Same gender as case
3. Same or nearby village or community as case
4. Concomitant: within 14 days of presentation of the index case
5. No diarrhea within 7 days of enrollment
6. Participant in the demographic surveillance system in the site involved

Note – A case and control pair can be bilateral. If a child selected as a control subsequently develops diarrhea at a later date, the original diarrhea case can serve as a control for the child who was previously a control and who is now a case.

A child must provide an adequate stool sample for microbiological analysis (≥5 g or the size of 5 peas, Kenya will remain at 4 g) during the required time period to be included in the analysis. The outcome of a control will be tabulated as follows:

a. Eligible and contacted but not enrolled because
   i. Child died
   ii. Adequate stool sample was not provided
b. Enrolled
c. Refused (tabulated as):
   i. No parent/caretaker available
   ii. Parent/caretaker too busy
   iii. Do not like research
   iv. Child too sick
   v. Other (specify)
d. Not contacted
   i. Another control was identified first (e.g., another control consented and contributed an appropriate stool sample first)
   ii. Could not locate child
e. Other reason (specify….)

Table 4. Allowable age range (in months) of matched controls by age of index case

	Matched control			Matched control			Matched control			Matched control			Matched control	
**Case**	**LL**	**UL**	**Case**	**LL**	**UL**	**Case**	**LL**	**UL**	**Case**	**LL**	**UL**	**Case**	**LL**	**UL**
**0**	0	2	**12**	12	16	**24**	24	28	**36**	32	40	**48**	44	52
**1**	0	3	**13**	12	17	**25**	24	29	**37**	33	41	**49**	45	53
**2**	0	4	**14**	12	18	**26**	24	30	**38**	34	42	**50**	46	54
**3**	1	5	**15**	12	19	**27**	24	31	**39**	35	43	**51**	47	55
**4**	2	6	**16**	12	20	**28**	24	32	**40**	36	44	**52**	48	56
**5**	3	7	**17**	13	21	**29**	25	33	**41**	37	45	**53**	49	57
**6**	4	8	**18**	14	22	**30**	26	34	**42**	38	46	**54**	50	58
**7**	5	9	**19**	15	23	**31**	27	35	**43**	39	47	**55**	51	59
**8**	6	10	**20**	16	23	**32**	28	36	**44**	40	48	**56**	52	59
**9**	7	11	**21**	17	23	**33**	29	37	**45**	41	49	**57**	53	59
**10**	8	11	**22**	18	23	**34**	30	38	**46**	42	50	**58**	54	59
**11**	9	11	**23**	19	23	**35**	31	39	**47**	43	51	**59**	55	59

LL=lower age limit; UL=upper age limit (in months)

22

In September, 2008, the protocol was revised to describe strategies to optimize the ability of Sentinel Health Centers (SHCs) to capture cases of MSD and to increase control enrollment improve statistical power in comparisons of cases vs. controls to compensate for observations that some sites were unable to meet the sample size requirements. The following modifications were undertaken:

1. Increase the control:case ratio

Each site continues to aim to enroll 8-9 cases and 8-9 matched controls in each age strata during each fortnight. However, during periods of slow recruitment when the required number of cases cannot be enrolled, a strategy will be implemented to enroll 2-3 controls per case in order to increase the power to detect differences between cases and controls for uncommon pathogens or those that are frequently excreted asymptomatically. **It is likely that all sites will need to recruit 2 or 3 controls for each case in the oldest age strata routinely because diarrhea is less common in this age group.** Since a matched pair analysis will be performed, confounding will not occur if the sites alter their case:control ratio depending on whether they are in high or low diarrheal incidence season.

Table 4a shows the impact that increasing the number of controls per case will have on the number of cases needed to achieve the desired power for comparing less common pathogens. For a pathogen-specific isolation rate of 2.5% in controls and 5.8% in cases (target isolation rates as stated in the protocol), this table shows the number of cases, controls and total sample size that would be needed to achieve 80% power (α=0.05, 2-sided).

Table 4a. Case-sparing effects of increasing control:case ratios

	**Case : Control Ratio**			
	**1: 1**	**1:2**	**1:3**	**1:4**
**Cases**	632	494	447	423
**Controls**	632	988	1341	1692
**Total**	1264	1482	1788	2115

It will be necessary for the sites to anticipate which seasons and in which age strata enrollment shortfalls are expected so that they can plan for enrollment of the additional controls. To this end, the sites have been offered the option of deciding to enroll the additional controls routinely only for the first week of each fortnight. If case recruitment is successful and it appears that a given stratum will be filled during the second week of that fortnight, they may revert to a lower case:control ratio. **However, each case must be matched with at least one control to be analyzable.**

2. Increase the number of eligible cases who are able to be enrolled

The initial experience with this study is that eligible cases often cannot be enrolled because a stool sample of sufficient volume was not available within 4 hours of the child's presentation to the sentinel health center. To increase the number of eligible cases, the following steps will be taken:

- The interval during which time a stool sample can be collected has been increased to **within 12 hours after registration** (rather than 4 hours); however, the sample must be collected before the child receives any antibiotics prescribed at the sentinel health center.

- A rectal swab may be obtained from any child who has not passed a stool before the acceptable collection time expires **IF** antibiotics will be administered imminently. **Nonetheless, a child who provides a rectal swab also must provide a whole stool within 12 hours of registration to be analyzable in the study.** If a child is not analyzable, matched controls will not be needed. The decision to evaluate the specimen in the microbiology laboratory and to perform a 60-day follow-up visit in such a situation where the child cannot produce a whole stool can be made on a site-by-site basis. The fact that the stool will be collected after administration of antibiotics should not affect the multiplex PCR and RT PCR assays for viruses or immunoassays for viral and protozoal antigens. However, the antibiotics will adversely affect the laboratory's ability to isolate various bacterial pathogens. This strategy will permit collection of an adequate sample for bacteriology prior to antibiotic administration as well as a whole stool for identification of pathogens that are best detected in whole stool but are not expected to be affected by antibiotic administration.
- In Mozambique, where many caretakers live a day's travel by foot from the SHCs, a stool will be induced if a caretaker wishes to leave the SHC before the 12 hour stool collection window has elapsed and if a caretaker lives in a remote or distant location such that field staff will not be able to reach her home to collect a sample within the 12 hour window. Stool will be induced by inserting a cotton-tipped rectal swab 1-2 cm into the rectum to attempt to induce passage of a whole stool.

It should be recognized that the case/control study will be analyzed by matched cases and controls. This gives us the flexibility to have additional controls per case during certain times of the year and for the ratio of cases to controls to differ somewhat among the sites. On the other hand, with a matched analysis, every case must have at least one control or the case is not analyzable.

Note: These strategies will be applied to the study of LSD, if necessary.

3. Increase the number of children with MSD who seek care at the SHCs

Analysis of the initial HUAS survey data indicated that utilization of the SHC by residents at our sites when their children had MSD was suboptimal and likely to contribute to under-enrollment. Whereas utilization of SHCs in Mozambique was 100%, much lower rates were seen at the other sites, ranging from 14% in India to 47% in The Gambia. Strategies have been developed at several sites to refer children with diarrhea who seek care elsewhere in the community to the SHCs. These strategies will **not** attempt to influence a caretaker's decision about whether to seek care outside the home, but rather to refer children who seek care elsewhere to the SHCs. In Mali, this strategy will target children seeking care from Traditional Healers, where the HUAS indicated that 67% of children with MSD are brought for care (vs. 36% to the SHC). In India, this strategy will involve setting up a medical clinic in two local administrative offices staffed by licensed study physicians who provide care for diarrhea and Oral Rehydration Solution for rehydration and/or prevention of dehydration free of charge. These referral strategies will adhere to the following guidelines:

- The management of diarrheal disease delivered by study physicians will follow the standard medical practice delivered at the local governmental clinics and hospitals. All children referred, **regardless of whether they are eligible for the study,** will receive an evaluation of their diarrheal illness by the study physician and will be given ORS packets free of charge. They will be given prescriptions for medications and referrals for additional care as appropriate.
- The referral system will represent a benefit to children with diarrhea because: 1) the child's evaluation and ORS packets will be free of charge, 2) the children will be managed by licensed physicians who have expertise in diarrheal diseases, and 3) if enrolled, the results

of the child's fecal cultures for bacterial pathogens and the immunoassays for rotavirus, *Giardia*, and *Cryptosporidium* will be provided to his/her health care provider and could potentially be useful in guiding their management. In the case of Mali, for example, the referred children would otherwise receive herbs, roots, and other remedies of uncertain safety and efficacy from the Traditional Healers.

- To encourage referrals, it is permissible for the study team to compensate the referring health care providers financially in order to offset income lost by referring the child. In Mali, the Traditional Healers are paid the equivalent of $1.00 for each child with diarrhea referred and $2.00 for each child included in the study.

6.1.6. Informed consent

The clinical protocol and subsequent amendments must be approved by the Institutional Review Board (IRB) of the University of Maryland, Baltimore (UMB) in addition to the relevant local IRBs overseeing each field site. The UMB IRB requires that all written or taped documents that are presented in a language other than English must be accompanied by a certificate from an independent observer verifying that the translation is true to the English consent form. The consent process will follow local customs, standards, and regulations. Groups that must provide approval are likely to include: 1) government and health authorities at the national, region, local, and hospital or health center levels, as appropriate; and 2) local community representatives, elders, religious leaders, etc.

Written, informed consent will be obtained from the parent or primary caretaker of each case and control who meets all eligibility criteria before any research activities are performed. First the study will be explained in local language or dialect. The parent or primary caretaker will be given a copy of the consent form to read or share with confidents who are able to read. At centers where illiterate parents are encountered, the consent form may be translated into the relevant local languages and recorded on audiotapes for the parents to hear. After these informational sessions are completed, the parent or primary caretaker will be given ample opportunity to ask questions. Thereafter, consent will be documented by asking the parent or primary caretaker to sign his/her name (or place an "x" or a fingerprint if unable to sign his/her name) on the consent form. If the parent/primary caretaker cannot read or cannot write, an impartial third party will witness the entire consent process and sign the consent document. The original signed/imprinted form will be retained at the site and the parent or primary caretaker will be given a copy to keep.

6.1.7. Clinical and epidemiologic data collection at enrollment

After informed consent has been obtained, the investigator will administer a standardized questionnaire to the parent/primary caretaker of the case or control. The questionnaire will include the following information:

1. Identifying information - The child's name, address, and other identifiers that will permit study personnel to perform a home visit 60 days after enrollment, as well as the child's study number and initials. This form will remain at the site under secure conditions and will not be transmitted off-site to the central database. Only authorized personnel will have permission to access the data.

2. Clinical/Epidemiology information - Approximately 60 questions are asked concerning epidemiologic and clinical details about the child and his/her diarrheal illness. Accuracy of age estimates is critical to assessments such as height and weight z score. In some

field sites (particularly in sub-Saharan Africa), birth date may not be known and it may be necessary to use an events calendar to estimate ages. The interviewer will assess the child's hydration status, and record observations about any stools that are passed. He/she will measure the child's axillary temperature, height and weight according to the methods described below in section 6.11.

6.1.8. Memory aid to track duration of diarrhea

A primary caretaker will be given a card and appropriate supplies to record whether the child experiences diarrhea for the 14 days after enrollment. To allow illiterate parents/caretakers to complete the form, the form will be pictorial and a different symbol will be shown to record whether there is diarrhea or not on a given day. A diarrheal day is defined as a day with 3 or more loose stools. The episode ends when 7 consecutive days pass without diarrhea. This instrument will be critical for detecting cases of persistent diarrhea. The memory aid was created in collaboration with the Malian Office of Literacy and the University of Bamako Faculty of Medicine IRB. It will be piloted and modified, as appropriate, to be compatible with each site.

6.1.9. Clinical and epidemiologic data collection at follow-up

Approximately 60 days after enrollment (range, 50 to 90 days), a field worker will visit the home of each case and control. A standardized questionnaire will be administered to cases and controls to ascertain the health status of the child. The **Memory Aid** will be reviewed with the parent or primary caretaker. Missing or unclear markings will be resolved, and the interviewer will sign and date the form. The child's axillary temperature will be recorded, as will the child's height, weight, and mid-upper arm circumference (section 6.11). Observations about water and sanitation facilities will be performed, and the drinking water will be tested for chlorine in households that say they treat their water.

6.1.10. Water, Sanitation, and Hygiene Sub-studies (Mali, Pakistan and Gambia only, already approved by UMB IRB under protocol modification #7 and by relevant local IRBs)

In Gambia and Pakistan a home visit will be made to participants who indicated on the enrollment questionnaire that they use a cloth to filter their drinking water. This visit will take place after the 60 day follow-up visit occurs. Thereafter, samples of drinking water will be collected from a subset of cases and controls to assay for microbial contamination. Households which have water collected will also be administered an additional Water, Sanitation, and Hygiene (WSH) questionnaire. In Mali, a random sample of sixty (60) households in the DSS will be visited at intervals to collect municipally supplied drinking water for microbiological analysis. The results of the lab evaluation will not be provided to individual households. However, each participating household will be provided with educational materials describing the use of locally recommended methods for treating drinking water to remove microbial contaminants.

6.1.11. HIV Substudies

6.1.11.1. Mozambique Counseling and Testing Substudy (already approved by UMB IRB under protocol modification #6 and by relevant local IRBs)

Within the case control study, we aim to offer voluntary counseling and HIV testing to cases and controls at the Mozambique site. Written, informed consent for HIV testing will be sought from the parent or primary caretaker, and voluntary counseling and testing will follow national guidelines. If the parent or caretaker refuses consent, the child will continue with other study procedures for which consent has been obtained. The child who meets the definition for HIV infection according to national guidelines will be referred to the Manhiça District Hospital to be

followed at the HIV regular outpatient clinic visits where counselling, management and treatment will be offered when required according to the national policy guidelines.

6.1.11.2. Kenya HIV Data Abstraction from the Demographic Surveillance System (approved by UMB under protocol modification #8 and by relevant local IRBs)

Kenya performs Provider-Initiated Counseling and Testing (PICT) and Home-Based Counseling and Testing (HBCT) for HIV through its national program. The site has received local IRB approval to link the test results with the individual's DSS number. We have an approved amendment to link the HIV status information to the GEMS data on diarrheal illness to determine the impact of HIV on the child's illness.

6.1.12. Verbal autopsy in the event that a child dies within 60 days of enrollment

If a child dies while in the hospital or health center, if the parent/primary caretaker reports that the child has died when the 60-day visit is made, or if a death is detected during ongoing demographic surveillance, then information on the cause of death will be collected in a standardized fashion from the medical chart, the health care provider, and, if available, the death certificate. In collaboration with the demographic surveillance system at the site, the caretaker will be interviewed by trained staff to elucidate signs, symptoms, and health seeking behavior during the terminal illness of the child.

6.1.13. Measuring height and weight

The height, weight and mid-upper arm circumference (MUAC) will be measured for each case and each control at enrollment and again 60 days later. Height and MUAC will be measured thrice. Methods are adapted from *How to Weigh and Measure Children: Assessing the Nutritional Status of Young Children in Household Surveys*, United Nations Department of Technical Cooperation for Development and Statistical Office, 1986. The height of children 0-23 months of age or those too ill to stand will be measured (to the nearest 0.1 cm) in the recumbent position using a board with a fixed head and sliding foot piece. For children 24 months and older who can stand alone, a standing height will be measured (to the nearest 0.1 cm). Weight will be measured to the nearest 0.1 kg using a scale that is calibrated daily. Each of the indices will be expressed in standard deviation units (SD) from the median of the NCHS/CDC/WHO International Reference Population.

6.1.14. Specimen collection

<u>Whole stool samples</u>

A single, fresh, whole stool specimen will be collected from both cases and controls at enrollment. In most instances the stool will be passed naturally per rectum. However, in some sites it is common practice to pass a small catheter into the child's rectum and aspirate stool using a syringe attached to the other end. The intention is to obtain at least 10g of stool; however, to be considered acceptable, the sample can weigh as few as 5 grams or 5 ml, or as an approximation resembles the size of 5 peas (Kenya will remain at 4 g). Study staff will leave a Styrofoam container containing a cold pack at the site (if at home or in a health care facility lacking a specimen refrigerator). The collector, i.e., individual collecting the stool (usually parent or family member) will either place a diaper (nappie) in a sealed plastic bag or scoop whole stool into a ~100 ml specimen cup (the cup should be no more than ½ full). The collector will immediately place the specimen into either a designated specimen refrigerator or a Styrofoam container containing a cold pack and transported to the laboratory in the time frame specified by the laboratory Manual of Procedures.

When study staff in the field retrieve the stool specimen, they will immediately process it as follows:

1. Insert a cotton swab into the specimen (if dysentery is present, the swab should be inserted into an area of blood or mucus), and place the soiled swab in a screw top plastic vial containing modified Cary Blair transport medium.
2. Insert a second stool swab into the specimen (if dysentery is present, the swab should be inserted into an area of blood or mucus), and place the soiled swab in a screw top vial containing Buffered Glycerol Saline (chilled, if possible).
3. Place at least 10 g (equivalent to ~10 ml) of whole stool into an empty vial (the stool that is already in the specimen container can be used for this purpose if available); a minimum of 5 g (5 ml, Kenya will remain at 4 g or 4 ml) is acceptable.
4. Put the vials in a sealed bag labeled with the child's study number, date and time of collection.
5. Place the bag immediately into either a specimen refrigerator or a Styrofoam container containing a fresh cold pack.
6. Deliver the specimen to the laboratory and plate within 18 hours of processing.

Rectal swabs (pertains only to selected cases, and not controls)

Each rectal swab will be moistened by dipping it in the medium that will be used for transport. The cottontip will be gently inserted into the child's rectum and rotated 360°. A properly collected rectal swab is stained or covered with fecal material. Two swabs may be inserted into the rectum simultaneously. The swabs will be processed and maintained at the temperatures described in the Manual of Procedures. The specimen will be delivered to the laboratory and plated within 18 hours of processing. Upon receipt in the laboratory, the specimens will be examined for acceptability: discoloration from fecal material, proper labeling, sealed containers (no leaks or cracks), and satisfactory low temperature of the transport container. The two swabs will then be sent to the microbiology bench for detection of bacterial pathogens.

Every attempt will be made to provide the results of cultures for bacterial pathogens and the immunoassays for rotavirus, *Giardia*, and *Cryptosporidium* to health care providers in a timely fashion.

Water collection for quantitative microbial analysis (Mali, Pakistan and Gambia only, already approved by UMB IRB under protocol modification #7)

Unfiltered water will be collected from the home, if available in unfiltered state, or from the reported primary water source where water is usually obtained. Two liters of the water will be collected into a sterile labeled two liter container. The caretaker will be provided two liters of sterile autoclaved water and will be asked to filter the water by their typical methods into a separate sterile labeled container. Next, the caretaker will be asked to filter the remaining two liters of unfiltered household water through the same cloth into a third sterile labeled container. Lastly, up to two liters of stored water, if available, will be collected into a fourth labeled sterile container. Details regarding storage will be collected from the WSH questionnaire. The pH, temperature, conductance, chlorine concentration, and turbidity of newly obtained water will be recorded immediately and water samples will be placed on ice in a cooler for transport to the laboratory facility within 2 hours of collection. Water will be processed by vacuum filtration for fecal and total coliform assays, quantitative bacterial colony-forming-unit (cfu) assays on selective differentiating media, and for RNA or DNA extraction for molecular quantitative PCR assays.

6.1.15. Sample size considerations

MSD case-control study

A sample size at each site of approximately 600 analyzable cases and 600 analyzable controls in each stratum was chosen for the initial six study sites, to provide 80% power for a normal-approximation test to detect a significant difference ($p<0.05$, two-sided) in site-stratum-specific isolation of a specific enteropathogen when cases are compared with controls, if the rate of isolation of that pathogen is as low as 5.8% in cases and 2.5% in controls (**Table 5**). To counteract dropout, migration and other losses to follow-up of up to 10%, we plan to enroll a total 660 cases and 660 controls per stratum per site to achieve the desired analyzable number of cases and controls. If the isolation rate of a pathogen in cases exceeds 5.8%, the absolute difference between cases and controls needed to achieve statistical significance will increase (but the relative difference will diminish). For example, this sample size will give 80% power to find a significant difference if the rate of isolation is 9.8% in cases vs. 5.5% or less in controls. Power will be somewhat less in the additional two to-be-named sites that will enroll subjects for only two years. This will be most apparent for uncommon pathogens or those with high asymptomatic colonization rates.

LSD case-control study

A sample size at each site of approximately 200 analyzable cases and 200 analyzable controls in each stratum was chosen to provide 80% power for a normal-approximation test to detect a significant difference ($p<0.05$, two-sided) in site-stratum-specific isolation of a specific enteropathogen, comparing cases and controls, if the rate of isolation of that pathogen is as low as 9.7% in cases and 2.5% in controls (Table 7). To counteract dropout, migration and other losses to follow-up of up to 10%, we plan to enroll a total of 220 cases and 220 controls per stratum per site to achieve the desired analyzable number of cases and controls. Implications of statistical power on analysis will follow as for the MSD study above. Multiple controls may be considered if case availability does not meet expectations, though this is considered unlikely.

Table 5. Isolation rates that will allow 80% power to find significant differences, assuming N cases & N matched controls per stratum per site over 3 years (α=0.05, 2-sided)

Pathogen-specific isolation rate in controls	Pathogen-specific isolation rate in cases				
	N = 500	N = 600	N=700	N=800	N = 900
1.5%	4.6%	4.2%	4.0%	3.8%	3.6%
2.5%	6.1%	5.8%	5.5%	5.2%	5.1%
3.5%	7.6%	7.2%	6.9%	6.6%	6.4%
4.5%	9.0%	8.5%	8.2%	7.9%	7.7%
5.5%	10.3%	9.8%	9.5%	9.2%	9.0%
7.5%	12.9%	12.4%	12.0%	11.7%	11.4%
9.5%	15.4%	14.8%	14.4%	14.1%	13.8%
11.5%	17.8%	17.2%	16.8%	16.4%	16.1%
13.5%	20.2%	19.5%	19.1%	18.7%	18.4%

If sites are unable to fulfill the requirements of a stratum, there may still be sufficient power to perform the necessary comparisons. Using the example cited above in which the isolation rate of a pathogen is 2.5% in controls, a sample size of only 500 would enable demonstration of statistically significant differences between cases and controls with 80% power if the isolation rate of a pathogen in cases were 6.1% (**Table 5**). The power to find significant differences will be substantially greater when the data are combined to assess trends occurring at all ages combined at a single site, at the regional level and at all sites combined.

Table 6. Isolation Rates for 80% Power to Find Significant Differences (α=0.025, 1-sided)

Isolation Rate, Controls	Isolation Rate in Cases					
	150 Cases		225 Cases		300 Cases	
	2 Controls/ Case	3 Controls/ Case	2 Controls/ Case	3 Controls/ Case	2 Controls/ Case	3 Controls/ Case
1.5%	7.5%	6.8%	6.0%	5.5%	5.2%	4.8%
2.5%	8.7%	8.0%	7.2%	6.8%	6.5%	6.1%
3.5%	10.4%	9.7%	8.8%	8.4%	8.0%	7.6%
4.5%	11.9%	11.3%	10.3%	9.8%	9.4%	9.0%
5.5%	13.5%	12.8%	11.8%	11.3%	10.8%	10.4%
7.5%	16.3%	15.6%	14.5%	14.0%	13.4%	13.0%
9.5%	19.1%	18.3%	17.1%	16.6%	16.0%	15.5%
11.5%	21.7%	21.0%	19.7%	19.1%	18.5%	18.0%
13.5%	24.3%	23.5%	22.1%	21.5%	20.9%	20.3%

Note: For control rate = 1.5%, power was calculated for a one-sided Fisher exact test; otherwise, for the normal-approximation z-test.

Table 7: **Isolation rates that will allow 80% power to find significant differences, assuming N cases & N matched controls per stratum per site over 1 year (α=0.05, 2-sided)**

**Pathogen-specific isolation rate in controls**	**Pathogen-specific isolation rate in cases**				
	**N = 100**	**N = 200**	**N=300**	**N=400**	**N = 500**
1.5%	10.6%	8.8%	6.2%	5.4%	4.8%
2.5%	14.5%	9.7%	7.9%	7.0%	6.3%
3.5%	16.3%	11.4%	9.4%	8.4%	7.8%
4.5%	18.0%	12.9%	10.9%	9.9%	9.2%
5.5%	19.6%	14.4%	12.3%	11.2%	10.5%
7.5%	12.3%	17.2%	15.1%	13.9%	13.1%
9.5%	12.5%	19.9%	17.7%	16.4%	15.6%
11.5%	28.4%	22.5%	20.2%	18.9%	18.0%
13.5%	31.0%	25.0%	22.6%	21.2%	20.3%

30

7.0 Case-Control Burden and Etiology Study: Laboratory Methods

7.1.1. Specimen accession

Each fecal specimen will comprise (a) a whole stool specimen (in screw top fecal specimen cups carried in Styrofoam boxes with cold packs), (b) a fecal swab in Modified Cary Blair medium in a plastic screw top test tube, and (c) a fecal swab in buffered glycerol saline in a screw top test tube. Each specimen will be bundled and each labeled with the subject's identification number, date and time of collection. Upon receipt in the laboratory, the specimen number will be entered into the computer database and a laboratory fecal specimen report form will be labeled with the subject's study number. The specimens will be examined for acceptability: sufficient volume (ideally 10 ml or grams with a minimum of 5 ml or grams, Kenya will remain at 4 ml or g), proper labeling, sealed containers (no leaks or cracks), and satisfactory low temperature of the transport container. The specimen will be examined for volume, consistency (standard 1-5 grade), presence of gross blood, pus, or mucus.

Upon accession, the specimens will be aliquoted into the following containers, with the quantities prioritized in an SOP:

1. Stool remaining after aliquoting will be sent to the microbiology bench for detection of bacterial pathogens.
2. ~2 grams of stool will be aliquoted to a single freezer vial, labeled with patient ID, for later processing for protozoal pathogens by immunoassay. The specimens will be placed at –20 ^{o}C or lower immediately after accession.
3. ~2 grams will be aliquoted to a single freezer vial, labeled with patient ID, for later processing for viral pathogens by immunoassay and RT-PCR. The specimens will be placed at –80°C immediately after accession.
4. ~1 g will be aliquoted equally to two freezer vials (i.e., 0.5 grams per vial), labeled with patient ID, for later processing for assays to detect additional pathogens, such as intestinal helminthes, *Helicobacter pylori*, toxigenic *Clostridium difficile* and *Bacteroides fragilis*. The specimens will be placed at –80°C immediately after accession.
5. ~5 grams will be aliquoted equally to two freezer vials (i.e., 2.5 grams per vial), labeled with patient ID, for later processing with tests not available at time of the study. The specimens will be placed at –80°C immediately after accession.

7.1.2. Specimen processing for bacterial pathogen detection

Fecal specimens will be plated on media for detection of bacterial pathogens according to standard methods, as described in the Laboratory Manual of Procedures. The following algorithm for microbiologic evaluation is a likely scenario **that will be subject to modification** in response to pilot testing and capability at the sites **without the need to modify the protocol**. **Amendments in these methods will be made in the Manual of Procedures and not the clinical protocol.**

Bacterial pathogens identified will include *Salmonella*, *Shigella*, *Campylobacter*, and possibly others such as *Aeromonas, Vibrio spp., Yersinia*. Other agents, such as toxigenic *Clostridium difficile or Bacteroides fragilis,* may be identified as well. Putative *Shigella* isolates will be serogrouped at the sites, and serotyped using monoclonal antibodies to identify *S. dysenteriae* 1 (if Group A) or the *S. flexneri* types and subtypes (if Group B).(24) Putative *S. flexneri* strains that do not react with the monoclonals will be agglutinated with specially prepared absorbed antisera provided by the WHO International *Shigella* Reference Laboratory at the Centers for Disease Control (CDC) to identify three new provisional *S. flexneri* serotypes. *Shigella* isolates from all sites will be sent to the CVD in Baltimore where they will be confirmed as *Shigella* and

preliminarily deposited in the Central Bacterial Strain Repository. Strains initially classified as *Shigella* Group A (other than *S. dysenteriae* 1), Group C, or as non-typeable strains will be trans-shipped to Dr. Nancy Strockbine at the International *Shigella* Reference Laboratory at the CDC where the strains will be serotyped. In addition, approximately 10% of strains from each site that were identified locally as *S. dysenteriae* 1, *S. flexneri* or *S. sonnei* will be sent to the CDC for verification (QC). A proportion of the non-typeable *Shigella* strains will be sent to Drs. Patrick Grimont at the Institut Pasteur in Paris. They will utilize molecular techniques to classify the non-typeable strains.(25)

Although no arrangements have been made for determining antibiotic resistances of the *Shigella* strains, the sites are encouraged to undertake such testing locally if they can accomplish this within their allotted budgets and according to Standard Operating Procedures of the NCCLS (National Committee for Clinical Laboratory Standards).

Isolates testing positive for *Vibrio* will be serogrouped (O1 or O139) and the O1 isolates serotyped (Inaba, Ogawa, Hikojima) from nutrient agar plates.(26) *Campylobacter* and *Vibrio* isolates will be frozen in duplicate.

Up to three colonies per specimen with the appearance of *E. coli* on MacConkey plates will be sub-cultured on nutrient agar, the growth harvested and frozen in duplicate. The vials will be labeled with date, identification number, and *E. coli* #1 – #3.

7.1.3. Specimen processing for *E. coli*

At a later time, the three colonies of *E. coli* will be will be analyzed using a multiplex PCR or another method will be used to identify diarrheagenic *E. coli* such as enterotoxigenic *E. coli* (ETEC), enteropathogenic *E. coli* (EPEC), enteroinvasive *E. coli* (EIEC), enterohemorrhagic *E. coli* (EHEC), enteroaggregative *E. coli* (EAggEC) and diffusely adherent *E. coli* (DAEC).(27-29) Diarrheagenic *E. coli* isolates will be shipped to the CVD in Baltimore, MD. From the CVD, ETEC strains will be trans-shipped to the laboratories of Dr. Roberto Vidal and Professor Valeria Prado in Santiago, Chile for PCR analysis to detect colonization factor antigens and for phenotypic expression of fimbrial antigens using monoclonal antibodies provided by Professor Ann-Mari Svennerholm of the University of Göteborg.

7.1.4. Detection of protozoal agents

Frozen stools dedicated for protozoal detection will be thawed and processed by immunoassay and/or genotype analysis for detection of *Entamoeba histolytica, Giardia intestinalis*, and *Crytosporidium* spp. *Giardia* and *Cryptosporidium* will be further speciated by PCR. The rationale for determining *Giardia* group A vs. B, and *C. hominis* vs. *C. parvum* vs. *C. meleagridis* is the emergence of data that indicate that these species/subspecies manifest distinct levels of pathogenicity resulting in different clinical features.(30;31) Nested substudies may be conducted comparing other methodologies for detection of these pathogens.

7.1.5. Detection of viral agents

The aliquot of the frozen stool dedicated for viral detection will be analyzed for group A rotavirus and enteric adenovirus using commercial enzyme immunoassays or genotype analysis. A subset of rotavirus positives will be G- and P-typed, and a subset of non-type 40 or 41 adenovirus positives may be typed by nucleotide sequencing after extraction of viral nucleic acid (see below). Noroviruses, sapoviruses, astroviruses will be tested by RT-PCR. A subset of astrovirus and norovirus samples may be genotyped by nucleotide sequencing and phylogenetic analysis.(32;33). Reference laboratories at the CDC, a WHO Regional Reference Laboratory or the WHO International Reference Laboratory at the CDC will be utilized for genotype analyses.

32

7.1.6. Environmental Microbiology Isolation and Quantification of pathogens (Mali, Pakistan and Gambia only, already approved by UMB IRB under protocol modification #7)

In water samples collected from the WSH cohort, samples will be aliquoted for multiple tests. A 150 ml water sample will be set aside for measurement of total and fecal coliform assays. Coliform assays will be conducted by vacuum filtration of a 100ml water sample, and two serial dilutions of the water through individual 0.22µm mixed cellulose esters membrane filters. The filters are then placed upon BBL mI agar for overnight culture at 35°C. Total and fecal coliforms will be enumerated around 18 hours. Isolation of bacterial pathogens will be conducted by membrane filtration of undiluted water and three ten-fold serial dilutions. These filters will be incubated overnight on selective media, per GEMS Manual of Procedures, for identification and enumeration of bacterial colony forming units. Several colonies of each identified pathogen will be selected for etiological validation by PCR using pathogen-specific primers. These colonies will also be sub-cultured and frozen for future study. A remaining liter of water will be membrane filtered for isolation of viral and protozoan pathogens. RNA and DNA extracts from the filter will be used for quantitative RT-PCR or PCR using pathogen-specific primers.

7.1.7. Central Repository for stool specimens and bacterial isolates, and specimens containing viral and protozoal pathogens

One of the most valuable assets generated by this large multi-site study will be an archive of stool and bacterial samples that can be used for future application of new developing technologies, for confirmation of ambiguous results, or for further characterization of the pathogens identified. Accordingly, at least 5 g of frozen stool from every subject, both cases and controls, in two equally divided aliquots in separate freezer vials, will be shipped to the CVD in Baltimore, Maryland for central storage at –80° C. These specimens will be stored in monitored, alarmed and locked freezers with identification of vials by patient ID number (without patient identifiers). The two aliquots from a single stool sample will be shipped and stored separately as a failsafe against inadvertent temperature fluctuations during shipment or storage. Use of these specimens will be restricted, and must be approved by application to the study sponsor.

Pathogenic bacterial strains isolated during this study and specimens containing gastroenteritis viruses and pathogenic protozoa will also be computer inventoried and archived in the Central Repository at CVD. It is possible that we will not have the resources to save all pathogens from all sites. Priority will be given to those requiring *post hoc* analysis, including *E. coli* and *Shigella* strains. Some strains, such as *E. coli* and *Shigella,* will be sub-cultured and trans-shipped to investigators at the CVD Microbiology laboratory, then will be trans-shipped to other investigators outside the CVD for further characterization. Parent stock cultures of all pathogens received will be retained at the CVD in dedicated, alarmed, and monitored freezers in perpetuity, in the event that the subcultures are lost, killed, or contaminated, and for future currently unanticipated characterization. A standard operating procedure will be developed to guide use of these specimens in compliance with ethical guidelines and site preferences.

7.1.8. Ethical considerations in testing stored specimens at collaborating centers

The following institutions, and possibly others, may receive clinical or epidemiologic data or stool specimens that are collected at the field sites during the course of this study:

- International Vaccine Institute, Seoul, KOREA
- Institut Pasteur, Paris, FRANCE

- Centers for Disease Control and Prevention, Atlanta, GA, USA
- Program in Microbiology and Mycology, University of Chile, Santiago, Chile
- Department of Medical Microbiology & Immunology, Univ. of Goteborg, Goteborg, SWEDEN
- University of Virginia School of Medicine, Charlottesville, VA, USA
- Center for International Health, University of Bergen, Bergen, NORWAY
- Rollins School of Public Health, Emory University, Atlanta, GA, USA
- Johns Hopkins University Bloomberg School of Public Health, Baltimore, MD, USA
- World Health Organization, Geneva, SWITZERLAND
- University of Melbourne, Victoria, AUSTRALIA

All personal identifiers will be removed from these specimens or data and the recipients at the collaborating sites will not have access to the code linking personal identifiers to sample numbers. Each of these sites will obtain local IRB approval according to local guidelines.

8.0 Data Management

8.1.1. Overview and description of the coordinating center

The Data Coordinating Center (DCC) responsible for centralized data management is the Cooperative Studies Program Coordinating Center (CSPCC), Veterans Administration Maryland Healthcare System, Perry Point, MD, USA, under the supervision of Dr. Joseph Collins. Hereafter, the CSPCC will be referred to as the Data Coordinating Center (DCC). The DataFax software system (Clinical DataFax Systems, Inc., Hamilton, Ontario, Canada) will be utilized for this purpose. The data base programmer at the DCC will develop the coding necessary to establish the data management system. This system will facilitate data capture, entry, and manipulation. The system's quality control functions will ensure that all variables are complete, within a specified acceptable range, and consistent (by performing cross-edits, across form edits and any special edits). User defined look-up tables will also be developed to assist in data verification. Other responsibilities of the DCC include generating queries for the sites to resolve missing or discrepant data and creating reports and reminders. DCC staff will send out weekly reports to notify the sites when the 60-day follow-up visit is due for each subject.

At the end of the study, the DCC will do a final clean-up of the study database working closely with the participating sites to ensure that all collected data have been received into the database and that all received data are as correct as can be made possible. Four to six months after the last patient follow-up, depending upon how responsive the sites are, the DCC will provide the study leadership with the final, locked study database.

8.1.2. Data entry and transmission

Within one week of the visit, all relevant case report forms (CRFs) will be completed, checked for quality, and transmitted to the DCC as follows. First, the forms will be scanned and stored as a pdf or tiff file on a designated secure personal computer at the site. A single file can contain CRFs of multiple types and from different subjects. Each site will systematically name and track the contents of these files. Daily, the files can be transmitted to the DCC via one of the following routes: 1) fax using an Internet connection (preferred); 2) fax using a phone line; 3) e-mail as an attachment; 4) if electronic or telephone transmission is not possible, the data can be stored on a compact disc (CD) and sent by mail.

34

8.1.3. Case report forms (CRFs)

All clinical and laboratory data will be collected on standardized CRFs. It is expected that each form will contain the questions in the English language and in the local language of the site; the sites will translate the forms into the relevant languages. The sites will also make the necessary changes to reflect local practices. The integrity of the fields will be maintained in the translated versions with dual languages to permit generation of a single integrated database containing the data collected at each site. The questions are largely multiple choice; however, if an open question is used that requires a response in text, the response must be translated to English before transmission to the DCC.

To maintain the confidentiality of the subjects, each CRF to be transmitted to DCC will be labeled only with a site number and patient ID number. A form that links personal identifiers to the study number will be maintained at each site but will not be transmitted to the DCC for entry into the common database. As described above, logs which contain a child's census number (which potentially could be linked to the child's identity) will be sent to the data coordinating center via email separate from the clinical and epidemiologic information that is collected and will be stored at the central data coordinating center in a secure location, separate from the case report forms.

The following case report forms will be transmitted to the DCC:

- Health Facilities Utilization and Attitudes Survey form
- Case Registration Log
- Control Registration Log
- Eligibility form – cases
- Eligibility form -- controls
- Enrollment form – cases
- Enrollment form – controls
- Memory Aid Score Sheet
- 60-day follow-up form for cases
- 60-day follow-up form for controls
- Verbal autopsy form for deaths occurring during initial hospitalization
- Verbal autopsy form for deaths detected at 60-day follow-up visit
- Specimen accession form
- Microbiology forms for bacterial, protozoal, and virologic analysis

The following forms will remain at the sites:

1. Personal identity form for HUAS (to remain at sites)
2. Personal identify form for case-control study (to remain at sites)
3. Duration of diarrhea Memory Aid (to remain at sites)

8.1.4. Quality control

Each day a supervisor or his/her designee will perform quality control (QC) by reviewing all forms completed by the staff that he/she is supervising for completeness and consistency. After discrepancies are resolved, he/she will sign the form indicating that it is ready for submission to the DCC. When DCC computer assistants queue the images from the fax server for verification, the system scans the images using intelligent character recognition and populates the appropriate database tables. The computer assistant staff will review the CRFs and data records within DataFax using a split-screen validation tool. Any discrepancies (i.e. missing data, range

validation, cross check) that are discovered during the verification process will be flagged with quality control notes for clinical site confirmation.

The DataFax system will include the following standard quality assurance procedures with each update:

- Queries flagged during validation
- Missing pages
- Audit trail of missing forms
- Tracking center performance
- Listing patient data
- Monitoring work flow
- Follow-up visit schedules
- Audit trail of error corrections

This combination of reports will allow the sites, the DCC and the study leadership to monitor the quality of the data. In addition, they will provide the tools necessary to identify sites that may be falling behind with the submission of their data and corrections.

On a fortnightly basis, Query Reports will be generated (in English) for variables that are flagged with quality control notes as possibly being in error. These reports are site specific and will be faxed directly to the clinical center for clarification. This report will give the identification information for the form in question (i.e. site number, patient identification number, form number, and/or visit number), the variable in question, and the reason the variable is being questioned. Sites will use the original CRF to mark the appropriate changes, and re-send the corrected page to the DataFax system. The DataFax application will check to determine if it has already received this page in the database. If the system determined that the form is currently in the database, a small dialog box will appear telling the verifier that they are reviewing a re-faxed page. When the user clicks the 'OK' button, the current data and QC notes are loaded from the database into the data window. The verifier proceeds to validate this with the new CRF page. The verifier can then execute changes to the data and/or resolve edit QC's as needed.

The DCC computer assistants will work closely with the staff at the sites to help them understand the problems that the query reports detect and to assist them with the corrections to these problems. The computer assistants, with guidance from the study leadership, will serve as a first-line resource to the participating sites on how to complete the study forms accurately.

8.1.5. Site-specific databases

On an annual basis, each site will receive an interim copy of the database containing the available data generated at their site, and a cleaned finalized version at the end of the study.

8.1.6. DCC Security

The DCC LAN has multiple security provisions in place, including password protected log-in, password protected screen savers, tracking of authorized and unauthorized log-in attempts, and exclusion of users who have repeated unsuccessful login attempts.

8.1.7. DCC Backup

Network backups (including the Alpha Server, Unix Server and the Novell Servers) are performed daily. Each backup will make a complete copy of all data residing on the network. Monthly backup tapes will be stored for one year from date of backup. Each weekly backup tape will be saved (off site) until the month end backup is performed, at which time the weekly backup

tape will be reused. Each daily backup tape will be saved until the weekend backup is performed, at which time the daily backup tapes will be reused. This rotation of backup tapes will insure file restoration capabilities for at least one year. These backups are maintained in a fireproof, combination-locked vault, both on and off-site. All tapes are labeled specifically, including system and date accordingly. Study data backups are performed routinely, on a monthly basis by the ITS to tape media or DVD-ROM (depending on the onset of study), or more often as deemed necessary, and are requested by members of the Study Team.

9.0 Analytic Methods

9.1.1. Definitions

1. **Age:** recorded in months, with the first month of life designated as “0”
2. **Diarrhea:** 3 or more abnormally loose or watery stools in a 24-hour period
3. **Dysentery:** Diarrhea with visible blood in one or more stools
4. **Persistent diarrhea**: Diarrhea lasting 14 days or longer
5. **Diarrhea episode**: Contiguous days with diarrhea ending when diarrhea is not present for 7 days.
6. **Fever:** Child feels febrile according to the parent or axillary temperature exceeds 38.0°C.
7. **Stunting:** below –2 standard deviations (SDs, *moderate*) or below –3 SDs (*severe*) from the median height-for-age of the reference population (results of two height measures will be averaged).
8. **Underweight**: below –2 SDs (*moderate*) or below –3 SDs (*severe*) from the median weight-for-age of the reference population
9. **Wasting:** below –2 SDs (*moderate*) or below –3 SDs (*severe*) from the median weight-for-height of the reference population
10. **Moderate-to-severe diarrhea**: diarrhea plus any of the following
    a. Moderate-to-severe dehydration, defined as the presence of one of the following: sunken eyes, more than normal, decreased skin turgor, or intravenous rehydration administered;
    b. Dysentery (diarrhea with visible blood in stool); or
    c. Hospitalized with diarrhea or dysentery.
11. **Less severe diarrhea:** diarrhea plus <u>none</u> of the following
    a. Sunken eyes, more than normal,
    b. Decreased skin turgor,
    c. Intravenous rehydration administered;
    d. Dysentery (diarrhea with visible blood in stool); or
    e. Hospitalized with diarrhea or dysentery.
12. **Profuse watery diarrhea:** is present when either:
    a. Requirement for continuing oral or IV replacement of stool water and electrolyte losses after initial successful replacement of the fluid deficit. (Note - This definition uses input rather than output per se).

b. Continued purging of large volumes of watery, rice water-like stools after successful replacement by rehydration of the fluid deficit, or;

c. 20 ml or more/kg of stool output is evident during a 4-h observation period after successful deficit rehydration (i.e., complete replacement of fluid deficit) (34).

13. **Socioeconomic status:** a model will be constructed for each site containing variables relevant to each community and ranking the households within the community. For example, in Africa the parameters will include: number of people in household per room, predominant floor material, presence of electricity, and a functioning radio, television, refrigerator, bicycle, car or truck.
14. **Access to improved water:** use of any of the following types of water supply for drinking: piped water, public tap, borehole or pump, protected well, protected spring or rainwater. Improved water sources do not include vendor-provided waters, bottled water, tanker trucks or unprotected wells and springs, rivers or ponds.
15. **Improved sanitation facilities** include: connection to a public sewer or septic system, pour-flush latrine, simple pit latrine, or ventilated improved pit latrine. Unimproved sanitation facilities include public or shared latrine, open pit latrine, bucket latrine.
16. **Oral rehydration therapy** (increased fluids [oral rehydration salt solution or government recommended home fluids] plus continued feeding) (35).
17. **Etiology:** if a single pathogen is isolated, the episode will be attributed to that agent; if multiple pathogens are identified, they will be classified as either multiple bacterial pathogens, multiple viral pathogens, multiple protozoal pathogens, or polymicobial.
18. **Household:** a group of people who live together and eat together, sharing a kitchen or cooking fire. A house or compound may contain more than one household.
19. **Pathogenicity index:** of an etiologic agent is defined by its ratio of isolation among cases divided by the isolation rate of that pathogen among controls.

9.1.2. Endpoints

<u>Primary Endpoints:</u>

Note: Each Primary endpoint will be determined for the following strata:

- All ages (0-59 months) over all sites combined
- All ages by region (Africa and Asia)
- All ages (0-59 months) at each site
- By age stratum (0-11, 12-23, and 24-59 months) over all sites combined
- By age stratum (0-11, 12-23, and 24-59 months) in each region (Africa or Asia)
- By age stratum (0-11, 12-23, and 24-59 months) at each site

---

1. Population-based incidence of MSD and LSD
2. Population-based incidence of pathogen-specific MSD and LSD
3. Population-based incidence of MSD and LSD by clinical presentation (non-bloody diarrhea, dysentery, or profuse watery diarrhea)
4. Case-fatality ratio of MSD and LSD
5. Case-fatality ratio of pathogen-specific MSD and LSD

6. Case-fatality ratio by clinical presentation (non-bloody diarrhea, dysentery, or profuse watery diarrhea)
7. Frequency of persistent diarrhea following an episode of MSD and LSD
8. Frequency of persistent diarrhea following an episode of pathogen-specific moderate-to-severe non-bloody diarrhea and LSD
9. Frequency of persistent diarrhea following an episode of MSD and LSD, by clinical presentation (non-bloody diarrhea, dysentery, or profuse watery diarrhea)
10. Frequency of nutritional faltering (underweight, wasting, or stunting) following an episode of MSD and LSD
11. Frequency of nutritional faltering (underweight, wasting, or stunting) following an episode of pathogen-specific MSD and LSD
12. Frequency of nutritional faltering (underweight, wasting, or stunting) following an episode of MSD and LSD, by clinical presentation (non-bloody diarrhea, dysentery, or profuse watery diarrhea)
13. Relative pathogenicity ("Pathogenicity Index") of each putative etiologic agent in association with MSD and LSD
14. Relative pathogenicity ("Pathogenicity Index") of each putative etiologic agent in relation to different clinical presentations (non-bloody diarrhea, dysentery, or profuse watery diarrhea)
15. Relative pathogenicity ("Pathogenicity Index") of each putative etiologic agent in relation to persistent diarrhea
16. Annual risk of MSD and LSD in children with and without each putative etiologic agent
17. Annual risk of each clinical presentation of MSD and LSD in children who do an do not have each putative etiologic agent
18. Annual risk of persistent diarrhea in children who have each putative etiologic agent
19. Relative risk of MSD and LSD in children with the pathogen, compared with children who do not have the pathogen
20. Relative risk of each clinical presentation of MSD and LSD in children with the pathogen, compared with children who do not have the pathogen
21. Relative risk of persistent diarrhea in children with the pathogen, compared with children who do not have the pathogen
22. Population-based pathogen-specific MSD and LSD attributable disease burden for the various enteric pathogens.

Secondary Endpoints:

Note: Secondary endpoints 1-4 will be determined for the following strata:

- All ages (0-59 months) over all sites combined
- All ages by region (Africa and Asia)
- All ages (0-59 months) at each site
- By age stratum (0-11, 12-23, and 24-59 months) over all sites combined
- By age stratum (0-11, 12-23, and 24-59 months) in each region (Africa or Asia)

- By age stratum (0-11, 12-23, and 24-59 months) at each site

---

1. Proportion of *Shigella* episodes attributed to each serotype
2. Proportion of ETEC infections with the following antigenic properties, alone or in combination (LT only, ST only, LT/ST, and specific CFAs, to include CFA/I, CS1-6, CS7, CS8, CS12, and CS17. Additional CS fimbriae may be identified subsequently if the Micriobiology Steering Committee so advises).
3. Proportion of *Giardia* and *Cryptosporidium* episodes attributed to each *Giardia* subspecies and *Cryptosporidium* species
4. Proportion of group A rotavirus episodes attributed to the different G and P types and of norovirus infections attributed to different genotypes

---

Note: Secondary endpoints 5-10 will be determined for the following populations:

- At each site
- Over all sites combined
- By region (Africa and Asia)

---

5. The factors that are independently associated with an episode of MSD and LSD by multivariable analysis, by comparing factors such as age, socioeconomic status, breast feeding, nutritional state, and level of sanitation and hygiene in cases and control children
6. The factors that are independently associated with an episode of pathogen-specific MSD and LSD by multivariable analysis, by comparing factors such as age, socioeconomic status, breast feeding, nutritional state, and level of sanitation and hygiene in cases and control children
7. The factors that are associated with death within ~60 days after an episode of MSD and LSD using a multivariable model that includes independent variables such as age, etiologic agent, case/control status, socioeconomic level, breast feeding, baseline nutritional status, and level of sanitation and hygiene
8. The factors that are associated with persistence of diarrhea for 14 days or longer using a multivariable model that includes independent variables such as age, etiologic agent, socioeconomic level, breast feeding, baseline nutritional status, and level of sanitation and hygiene
9. The factors that are associated with nutritional faltering within ~60 days following an episode of MSD and LSD using a multivariable model that includes independent variables such as age, etiologic agent, socioeconomic level, breast feeding, baseline nutritional state, and level of sanitation and hygiene
10. The public and private financial costs, both direct and indirect, incurred in treating an episode of MSD and LSD in a child 0-59 months of age
11. Creation of a central repository of well-characterized clinical specimens and isolated etiologic agents that can be accessed for further evaluation by approved investigators.

9.1.3. Calculation of primary endpoints

1. Population-based incidence rates and risks

The ability to calculate the population-based incidence of diarrhea overall and by pathogen will enable us to estimate the burden of disease among children living in the communities under surveillance. Population-based estimates of the **annual incidence rate of MSD and LSD** per 100,000 children (primary endpoints 1-3, section 9.3) and the risk (probability) of MSD and LSD for all children, by age group, according to pathogen, and by clinical presentation, will be calculated using overall population census data as well as data from the HUAS and the case-control study. Risk in children with and without a specified pathogen, as well as relative risk and attributable risk (primary endpoints 16-22) for MSD and LSD according to pathogen, will also be estimated. The incidence rates will be calculated by age stratum for each individual site, by global region (sub-Saharan Africa and Asia) and across all sites. The population-based incidence of **pathogen-specific MSD and LSD** will also be calculated at each individual site, by region and across all sites, according to age stratum, clinical presentation, and among all groups combined. The estimation procedure for MSD and LSD is as follows.

Let D and D* indicate MSD/LSD and the absence of MSD/LSD, respectively. (Note – D includes both eligible and ineligible children with MSD/LSD). Let E and E* indicate the presence and absence, respectively, of a specified pathogen, e.g., rotavirus.

Calculations for the LSD study will proceed as above, but with LSD replacing MSD.

The **risk of diarrheal disease** associated with each pathogen and the **relative risk** (compared to children without the pathogen) will be estimated using case-control, HUAS, and population census data. Relative risk will be calculated for the etiologic agents of interest at each site, in each region and across all sites, according to age stratum and among the combined age groups in relation to each of the following categories:

- All MSD
- Clinical presentation (either non-bloody diarrhea, dysentery, or profuse watery diarrhea)
- Persistent diarrhea
- LSD study: as above, but replacing LSD for MSD. Dysentery will not be analyzable because it is an exclusion criterion for the LSD study.

a. *Risk in children with and without a specified pathogen:*

- From the case-control study, we will have estimates N_{DH} of the total number of children per year with D who present at a health care facility; $p_{E|D}$ and $p_{E|D*}$, of the proportions of children with and without D who have the specified pathogen.

- From the HUAS, we will have an estimate $p_{H|D}$ of the proportion of all children (or episodes) of D who are taken to a health care facility.

- From the most recent census, we will have an estimate N of the total population in the relevant age group living in the site's catchment area.

- Then estimates of the total children in the relevant age group who live in the site's catchment area and have D or don't have D during the year are $N_D = N_{DH} / p_{H|D}$ and $N_{D*} = N - N_D$.

- Estimates of the number of children with D who have and don't have the pathogen are $N_{DE} = N_D\, p_{E|D}$ and $N_{DE*} = N_D - N_{DE}$.

- Similarly, estimates of the number of children without D who have and do not have the pathogen are $N_{D^*E} = N_{D^*} p_{E|D^*}$ and $N_{D^*E^*} = N_{D^*} - N_{D^*E}$.

- Estimates of the number of children who have and do not have the pathogen during a year are $N_E = N_{DE} + N_{D^*E}$ and $N_{E^*} = N - N_E$.

- Finally, estimates of the annual risk of D in children who have and do not have the pathogen are $p_{D|E} = N_{DE} / N_E$ and $p_{D|E^*} = N_{DE^*} / N_{E^*}$.

b. Relative risk

- The relative risk of D in children with the pathogen, compared to children without the pathogen, is estimated by $p_{D|E} / p_{D|E^*}$.

c. Attributable risk

- The attributable risk (AR) of D when E is present – the portion of the risk of D in children with E that can be attributed to E (i.e., may be caused by E) is $p_{D|E} - p_{D|E^*}$.
- The attributable risk burden of diarrheal disease – the number of cases attributable to E and therefore potentially preventable by a vaccine against E – is estimated by $N_E (p_{D|E} - p_{D|E^*}) = N_{DE} - N_E p_{D|E^*}$.

d. Incidence rates

- From the case-control study we will also have an estimate T_{DH} of the total number of episodes of D per year brought to a health care facility.
- The total number of episodes of D in a year is then estimated by $T_D = T_{DH} / p_{H|D}$.
- The numbers of episodes with E present and absent, respectively, are estimated by $T_{DE} = T_D p_{E|D}$ and $T_{DE^*} = T_D - T_{DE}$.
- The incidence rates in, the population, of all MSD and of diarrhea with the specified pathogen present, per 100,000 child-years, are given by 100,000 T_D / N and 100,000 T_{DE} / N.
- Incidence among children who have E present is 100,000 T_{DE} / N_E.

2. **Frequency of death and other adverse sequelae**

Case-fatality ratios (primary endpoints 4-6) will be calculated by determining the number of enrolled children in a given category who die within 60 days (range 50-90 days) of enrollment divided by the number of enrolled children belonging to that category. Similarly the proportion of control children who die over the 60-day period will be calculated. The impact of cases that die at home without reaching the site's health care facilities will also be evaluated. Rates of **persistent diarrhea** (primary endpoints 7-9) and of the three types of **nutritional faltering** (underweight, wasting, and stunting, as defined in section 9.1) (primary endpoints 10-12) will be calculated by substituting the number of cases who develop each of these endpoints within 60 days of enrollment (range 50-90 days) for cases who die. For the analyses of nutritional faltering, only those children who did not meet the case definitions at enrollment will be included in this calculation.

The frequency of case fatality, nutritional faltering, persistent diarrhea, and persistent diarrhea associated with death will be calculated at each site, by region (Asia and Africa) and

across all sites, according to age stratum and among all age groups combined, for the following conditions:

- All MSD
- Pathogen-specific MSD
- Clinical presentation (either non-bloody diarrhea, dysentery, or profuse watery diarrhea)
- LSD study: as above, but replacing LSD for MSD.

3. Population-based estimates of disease burden

The **population-based, pathogen-specific, MSD-associated disease burden** and the **population-based, pathogen-specific, MSD-associated attributable disease burden** for the various enteric pathogens will be estimated. It must be recognized that not all cases of MSD in which the pathogen is isolated in fact have diarrhea caused by this agent because the pathogen is also isolated from some control children without diarrhea (i.e., who have asymptomatic infection). This is why the **population-based, pathogen-specific, MSD-associated disease burden** without correction does not offer a valid measure of the true burden caused by a pathogen. Accordingly, the **population-based, pathogen-specific, MSD attributable disease burden** will be estimated for all MSD and in relation to the different clinical presentations (non-bloody diarrhea, dysentery, or profuse watery diarrhea):

- For all ages, 0-59 months, over all sites combined
- Among all ages by region (Africa, Asia)
- Among all ages at each individual site
- By age stratum over all sites combined
- By age stratum and region
- By age stratum at each individual site

Note -- The **population-based, pathogen-specific, MSD attributable disease burden** is important, as it can be used in estimating the number of cases that could be prevented by use of a specific vaccine of a particular efficacy and with different estimates of vaccine coverage. (The indirect protective effect of the vaccine on non-vaccinated subjects can also be added to the model).

The LSD study will be analyzed as above, but with LSD replacing MSD.

4. Analysis of microbiologic data

A **Pathogenicity Index** (primary endpoints 13-15) will be calculated for each putative etiologic agent as the ratio of isolation of the pathogen among cases divided by the isolation rate among controls.(4;23)

The **serotype distribution of strains of *Shigella*** at each site, in the African and Asian regions and across all sites, will be described according to age stratum and among all age groups combined.

The distribution of **enterotoxin type (heat-labile [LT], heat-stable [ST] or LT/ST) and antigenic types of fimbrial colonization factor antigens among enterotoxigenic *Escherichia coli* (ETEC)** strains isolated at each site, in each region and across all sites, will be described according to age stratum and among all age groups combined. Colonization factor antigens looked for will include CFA/I, CS1-6, CS7, CS8, CS12 and CS17. (Additional CS fimbriae and alleles of other adhesins may be looked for subsequently if the Microbiology Committee advises this action).

43

The **sub-species of *Giardia*** infections and the **species of *Cryptosporidium*** infections at each site, in each region and across all sites will be characterized, according to age stratum and among all age groups combined.

The **G and P type distribution among Group A rotavirus** infections and the **genotypes of norovirus** infections will be described at each site, in each region and across all sites, according to age stratum and among all age groups combined.

9.1.4. Calculation of secondary endpoints

9.4.1 Risk factors for MSD

Secondary endpoints 1-4 and 10 will be analyzed descriptively. A series of multivariable analyses (secondary endpoints 5-9) will be used to identify **independent risk factors of MSD** at each site, in each region, and across all sites by constructing models that include age, socioeconomic level, breastfeeding, baseline nutritional condition, and level of sanitation and hygiene as independent variables in cases vs. control children, and the following as independent variables:

- All MSD
- Diarrhea associated with specific pathogens.

A series of multivariable analyses will be performed to determine independent **predictors of adverse clinical consequences (sequelae) of diarrhea** (secondary endpoints 7-9) at each site, in each region and across all sites by constructing models that include putative etiologic agents, age, socioeconomic level, breastfeeding, baseline nutritional state, and level of sanitation and hygiene as independent variables, and the following as dependent variables:

- Persistent diarrhea
- Nutritional faltering
- Death

9.4.2 Risk factors for less severe diarrhea

As with section 9.4.1 above, but with LSD in place of MSD.

9.4.3 Economic analyses and the introduction case for selected vaccines in study countries

Dr. Richard Rheingans at Emory University and Dr. Damian Walker at Johns Hopkins School of Public Health will undertake economic studies to:

a. Estimate the economic burden of diarrheal diseases in selected countries (by etiology)

- Estimate the direct medical, direct non-medical and indirect costs per episode of MSD (or LSD) in each of the selected countries;
- Estimate the national annual economic burden of MSD (or LSD) in each country;
- Describe the distribution of disease and economic burden among socio-economic groups;
- Describe the distribution of disease and economic burden by perspective (societal, government, healthcare system, households).

b. Estimate the cost-effectiveness of vaccination against the major diarrheal pathogens in different settings

- Develop a generalized model of the costs and benefits of vaccination against diarrheal diseases;
- For each country, develop a comparison of the costs, benefits and cost-effectiveness associated with vaccination against each of the major pathogens (using estimated disease and economic burden data, expected or potential efficacy and estimated costs of introduction);
- For each country characterize the types of vaccines that would be most cost-effective (based on price, efficacy and the incidence of the pathogen targeted);
- Compare the cost-effectiveness of investing in vaccine development for different pathogens.

9.1.5. Expanded Risk Factor Studies and Environmental Microbiologic Studies

The clinical, epidemiological and microbiologic activities described in earlier sections of this proposal offer an unusual opportunity to study in a more intensive way the hierarchy of risk factors associated with diarrheal disease due to specific enteric pathogens and to correlate that with environmental microbiologic studies (e.g., pathogen detection and quantification in surface waters weaning foods, etc.). Once the main studies and activities are up and running and functioning well, beginning in Year 2, sites will have the option of participating in expanded studies to identify risk factors (as part of the case/control study). This will involve preparing detailed questionnaires compatible with the environmental and cultural conditions present at each individual field site. At the time of the 60-day visit, direct observational evaluations of the households can be undertaken (water supplies, human waste disposal, presence of soap near defecation sites, methods of storing weaning foods, etc.). Lastly, highly focused environmental microbiologic studies can be undertaken. The design of such studies would be closely coordinated with staff at the Bill and Melinda Gates Foundation who have interest in and responsibility for such studies to assure that they align with the goals and priorities of the Foundation. (Towards that end, should it be decided that such complementary nested studies are appropriate and can indeed be logistically sustained, a budget item to support such studies is inserted within the budget of each field site for years 2-4).

9.1.6. Statistical comparisons

Univariable comparisons will be performed to compare the distributions of risk factors for diarrhea among cases and controls. For dichotomous factors, a normal approximation test (z-test) or Fisher exact test will be used; for continuous variables, either a t-test or Wilcoxon rank-sum test will be used. Multivariable techniques (e.g., logistic regression) will be used to identify independent risk factors.

10.0 In-process Quality Assurance (QA)

QA will be rigorously performed throughout the course of the study. All forms will be reviewed daily by a supervisor for completeness, legibility, and internal consistency. The DataFax system will provide tracking reports to ensure timely and accurate completion of the CRFs. The database will contain ranges and Query Reports will be sent to the sites to resolve missing or inaccurate data. Field supervisors will perform random field checks to ensure validity of the data

collected by the field workers. All aspects of specimen accession, processing and interpretation will be performed according to rigorously standardized SOPs, made available to each site. Procedures will be instituted to optimize standardization across sites, e.g., by requiring uniform equipment, reagents, assays, etc. across sites and utilizing standard controls.

11.0 Monitoring Plan

11.1.1. Clinical and epidemiological activities

Monitoring will also be accomplished on an ongoing basis using the interactive DataFax data management system. This system allows periodic (weekly) assessment of the following:

- Participant recruitment, to assure the feasibility of meeting recruitment projections;
- Data quality and timeliness, missing forms, missing data, etc.;
- Completion of follow-up visits.

On-site monitoring will be performed by the PI and her assistants on a regular basis, at least 2 times per year. The site monitor will review the regulatory files and a random sample of ~20 subject charts (consent and case report forms) to ensure that the study is conducted and documented properly according to a pre-determined Monitoring Plan. The overall purpose of trial monitoring is to ensure that:

- The rights and well-being of human subjects are protected and that the consent process is followed per protocol and the consent forms are appropriately completed;
- The reported trial data are accurate, complete and verifiable from source documents;
- The conduct of the trial is in compliance with the currently approved protocol, with GCP and with applicable regulatory requirements.

Monitoring procedures will include:

- Verification that investigator qualifications, resources, staff, facilities, and equipment remain adequate;
- Verification that investigator follows the approved protocol and all approved amendments;
- Verification that written informed consent was obtained before each subject's participation in the trial;
- Verification that eligibility and enrollment logs are maintained;
- Verification that all case report forms and regulatory and other essential documents are accurate, complete, and up-to-date;
- Verification that all subjects meet eligibility requirements;
- Verification that all withdrawals, dropouts, and protocol deviations are appropriately reported and explained;
- Verification of compliance with QA procedures.

Findings of on-site monitoring visits will be presented to Coordinating and Site Investigators verbally and in a written report. The monitors will follow-up on deficiencies found during on-site visits to be certain that they are corrected or otherwise are addressed by the site in a timely fashion and that retraining or other appropriate corrective actions are taken.

46

11.1.2. Microbiological activities

The DataFax system will provide ongoing monitoring by tracking the results of microbiologic assays. Dr. Nataro and his assistants will review the results of the monthly QA controls for bacteriology, immunoassay and molecular biology assays performed in the laboratory. Twice annually, the microbiology monitors, known as the QA Task Force, will visit each site for a period of one week. The Task Force will bring unknown Proficiency Test (PT) specimens for bacteriology, virology and parasitology. The Task Force will inspect all records kept by the site lab, will answer questions regarding lab processes, and will observe the operations of the site, review the results of the PTs, and debrief the team. They will move to assist the site staff to rectify any deficiencies identified. At least one experienced member of the Task Force will be prepared to remain on that site as long as required to assure full and accurate compliance with all SOPs.

Findings of on-site monitoring visits will be presented to Coordinating and Site Investigators verbally and in a written report. The monitors will follow-up on deficiencies found during on-site visits to be certain that they are corrected or otherwise are addressed by the site in a timely fashion and that retraining or other appropriate corrective actions are taken.

12.0 Risks and Benefits

The risks to participating are minimal. Every effort will be made to maintain the confidentiality of all data collected. CRFs will be stored in locked file cabinets at the sites. The files can be accessed only by study personnel. Electronically transmitted data will be secured as described above. Forms that contain personal identifiers (the child's name, address, and other identifiers that will permit study personnel to perform a home visit 60 days after enrollment) will be maintained at the sites under secure conditions with limited access and not transmitted off-site to the central database; Only authorized personnel will have permission to access the data. The remaining CRFs will be labeled only with the child's study number. The data on the Census ID Log will be sent to the DCC. This log will include information from the census such as the child's census ID number, village name, census cluster number, compound number, household number, and global positioning system (GPS) coordinates. Although no names will be recorded, this information could potentially be linked to a child's identity. Therefore, the following precautions will be taken to maintain confidentiality: the log will be sent to the data coordinating center via email separate from the clinical and epidemiologic information that is collected and will be stored at the central data coordinating center in a secure location, separate from the case report forms.

Some of infants and children may experience adverse outcomes from their illnesses or even death. Study personnel will be trained to collect information about these illnesses in a respectful and sensitive fashion.

The results of the cultures for bacterial pathogens and the immunoassays for rotavirus, *Giardia*, and *Cryptosporidium* will be provided to the health care providers in a timely fashion. At some sites, this information is not normally available. An etiologic diagnosis may lead to more effective treatment of a child's illness. It is hoped that this study will guide the prioritization and development of strategies that will effectively prevent diarrheal illnesses and deaths in young children.

13.0 References

(1) World Health Organization. World Health Report 2005. Making every mother and child count. WHO Press; 2005.

(2) World Health Organization. Removing Obstacles to Healthy Development. Report on Infectious Diseases. 1999. Report No.: WHO/CDS/99.1.
(3) Black RE, Morris SS, Bryce J. Where and why are 10 million children dying every year? Lancet 2003 June 28;361(9376):2226-34.
(4) Hamer, D. H., Simon, J., Thea, D., and Keusch, G. T. Childhood diarrhea in sub-Saharan Africa. 1998. Report No.: Volume 2, Number 1.
(5) Black RE, Morris SS, Bryce J. Where and why are 10 million children dying every year? Lancet 2003 June 28;361(9376):2226-34.
(6) The global burden of disease: a comprehensive assessment of mortality and disability from diseases, injuries and risk factors in 1990 and projected to 2020. Harvard University Press; 1996.
(7) Kosek M, Bern C, Guerrant RL. The global burden of diarrhoeal disease, as estimated from studies published between 1992 and 2000. Bull World Health Organ 2003;81(3):197-204.
(8) Cooper RS, Osotimehin B, Kaufman JS, Forrester T. Disease burden in sub-Saharan Africa: what should we conclude in the absence of data? Lancet 1998 January 17;351(9097):208-10.
(9) Guerrant RL, Kosek M, Moore S, Lorntz B, Brantley R, Lima AA. Magnitude and impact of diarrheal diseases. Arch Med Res 2002 July;33(4):351-5.
(10) UNICEF. State of the World's Children 2005. Childhood under Threat. New York: UNICEF; 2005.
(11) Eisele TP, Lindblade KA, Rosen DH, Odhiambo F, Vulule JM, Slutsker L. Evaluating the completeness of demographic surveillance of children less than five years old in western Kenya: a capture-recapture approach. Am J Trop Med Hyg 2003 July;69(1):92-7.
(12) Fauveau V, Henry FJ, Briend A, Yunus M, Chakraborty J. Persistent diarrhea as a cause of childhood mortality in rural Bangladesh. Acta Paediatr Suppl 1992 September;381:12-4.:12-4.
(13) Shaikh K, Wojtyniak B, Mostafa G, Khan MU. Pattern of diarrhoeal deaths during 1966-1987 in a demographic surveillance area in rural Bangladesh. J Diarrhoeal Dis Res 1990 December;8(4):147-54.
(14) Etard JF, Le Hesran JY, Diallo A, Diallo JP, Ndiaye JL, Delaunay V. Childhood mortality and probable causes of death using verbal autopsy in Niakhar, Senegal, 1989-2000. Int J Epidemiol 2004 December;33(6):1286-92.
(15) Eisele TP, Lindblade KA, Rosen DH, Odhiambo F, Vulule JM, Slutsker L. Evaluating the completeness of demographic surveillance of children less than five years old in western Kenya: a capture-recapture approach. Am J Trop Med Hyg 2003 July;69(1):92-7.
(16) Fauveau V, Henry FJ, Briend A, Yunus M, Chakraborty J. Persistent diarrhea as a cause of childhood mortality in rural Bangladesh. Acta Paediatr Suppl 1992 September;381:12-4.:12-4.
(17) Shaikh K, Wojtyniak B, Mostafa G, Khan MU. Pattern of diarrhoeal deaths during 1966-1987 in a demographic surveillance area in rural Bangladesh. J Diarrhoeal Dis Res 1990 December;8(4):147-54.
(18) Etard JF, Le Hesran JY, Diallo A, Diallo JP, Ndiaye JL, Delaunay V. Childhood mortality and probable causes of death using verbal autopsy in Niakhar, Senegal, 1989-2000. Int J Epidemiol 2004 December;33(6):1286-92.
(19) World Health Organization. Generic protocols for: i) hospital-based surveillance to estimate the burden of rotavirus gastroenteritis in children and ii) a community-based survey on utilization of health care services for gastroenteritis in children. Geneva: WHO; 2002.
(20) World Health Organization VAaMTDoIVaB. Immunization coverage cluster survey -- Reference manual. 2005. Geneva, WHO Document Production Services.
Ref Type: Pamphlet

(21) Milligan P, Njie A, Bennett S. Comparison of two cluster sampling methods for health surveys in developing countries. Int J Epidemiol 2004 June;33(3):469-76.

(22) Ferreccio C, Prado V, Ojeda A, Cayazzo M, Abrego P, Guers L et al. Epidemiologic patterns of acute diarrhea and endemic *Shigella* infections in a poor periurban setting in Santiago, Chile. Am J Epidemiol 1991;134(6):614-27.

(23) Levine MM, Ferreccio C, Prado V, Cayazzo M, Abrego P, Martinez J et al. Epidemiologic studies of *Escherichia coli* infections in a low socioeconomic level periurban community in Santiago, Chile. Am J Epidemiol 1993;138:849-69.

(24) Carlin NI, Rahman M, Sack DA, Zaman A, Kay B, Lindberg AA. Use of monoclonal antibodies to type *Shigella flexneri* in Bangladesh. J Clin Microbiol 1989;27:1163-6.

(25) Coimbra RS, Grimont F, Grimont PA. Identification of Shigella serotypes by restriction of amplified O-antigen gene cluster. Res Microbiol 1999 October;150(8):543-53.

(26) Kaper JB, Morris JG, Jr., Levine MM. Cholera. Clin Microbiol Rev 1995;8:48-86.

(27) Vidal M, Kruger E, Duran C, Lagos R, Levine M, Toro C et al. Multiplex PCR for diagnosis of six diarrheagenic *Escherichia coli* categories. J Clin Mirobiol. In press 2005.

(28) Vidal R, Vidal M, Lagos R, Levine M, Prado V. Multiplex PCR for diagnosis of enteric infections associated with diarrheagenic *Escherichia coli*. J Clin Microbiol 2004 April;42(4):1787-9.

(29) Nguyen TV, Le VP, Le HC, Gia KN, Weintraub A. Detection and characterization of diarrheagenic *Escherichia coli* from young children in Hanoi, Vietnam. J Clin Microbiol 2005 February;43(2):755-60.

(30) Haque R, Roy S, Kabir M, Stroup SE, Mondal D, Houpt ER. *Giardia* assemblage A infection and diarrhea in Bangladesh. J Infect Dis. In press 2005.

(31) Houpt ER, Bushen OY, Sam NE, Kohli A, Asgharpour A, Ng CT et al. Asymptomatic *Cryptosporidium hominis* infection among human immundeficiency virus-infected patients in Tanzania. Am J Trop Med Hyg 2005 September;73(3):520-2.

(32) Noel JS, Lee TW, Kurtz JB, Glass RI, Monroe SS. Typing of human astroviruses from clinical isolates by enzyme immunoassay and nucleotide sequencing. J Clin Microbiol 1995 April;33(4):797-801.

(33) Ando T, Noel JS, Fankhauser RL. Genetic classification of "Norwalk-like viruses. J Infect Dis 2000 May;181 Suppl 2:S336-48.:S336-S348.

(34) Khan WA, Saha D, Rahman A, Salam MA, Bogaerts J, Bennish ML. Comparison of single-dose azithromycin and 12-dose, 3-day erythromycin for childhood cholera: a randomised, double-blind trial. Lancet 2002 November 30;360(9347):1722-7.

(35) Victora CG, Bryce J, Fontaine O, Monasch R. Reducing deaths from diarrhoea through oral rehydration therapy. Bull World Health Organ 2000;78(10):1246-55.

49

TESTING *ESCHERICHIA COLI* TRIPLETS ISOLATED FROM CULTURES OF STOOL SPECIMENS FROM GEMS-1A CASES AND THEIR MATCHED CONTROLS

INTRODUCTION

In the course of the GEMS-1A project, six of the seven field sites (Gambia, Mali, Mozambique, Bangladesh, India and Pakistan) will carry out, in parallel, two case/control studies examining the etiologic agents associated with diarrheal disease in infants and young children 0-59 months of age. One case/control study, as in GEMS-1, will investigate the etiology of moderate and severe diarrhea (MSD), while the second parallel study will investigate the etiology of less severe diarrhea (LSD) that does not meet the criteria for MSD. The seventh GEMS-1A site, Kenya, will continue its study of MSD in a high HIV prevalence area.

Escherichia coli isolates from GEMS-1A patients with diarrheal illness and from their matched controls in each of the case/control studies will be tested as mixes of three colonies ("triplets") picked from the same MacConkey's agar plate. These triplets of *E. coli* colonies from stool cultures of cases and controls will be tested for the presence of diarrheagenic *E. coli* using two Multiplex PCRs virtually identical to what was used in GEMS-1. In this way, the work-up for diarrheagenic *E. coli* in GEMS-1A will have a direct bridge to the methodology used in GEMS-1. However, based on lessons learned from analyses of data from GEMS-1, all *E. coli* triplets in GEMS-1A will also be tested with a new Duplex PCR and some triplets will also be tested with a Monoplex for *bfpA*. These various PCRs are explained below, followed by a summary of the work flow for their use.

SCREENING PCRs

GEMS *E. coli* Multiplex PCR #1.

This multiplex contains primers to amplify genes that identify enterotoxigenic *E. coli* (ETEC), enteropathogenic *E. coli* (EPEC), enteroaggregative *E. coli* (EAEC) and that screen for the presence of enterohemorrhagic *E. coli* (triplets found to be positive for *eae* but negative for *bfp* using Multiplex #1 are tested using Multiplex PCR #2; positivity for *stx* [Shiga toxin] indicates EHEC).

Gene targets for the specific primers in Multiplex #1 include:

For ETEC --

eltB – Encodes the B (binding) subunit of heat-labile enterotoxin of enterotoxigenic *E. coli* (ETEC).

estA – Encodes the heat-stable enterotoxin (designated in the literature as STa or STI) found among enterotoxigenic *E. coli* (ETEC) isolated from humans with ETEC diarrhea. There are two variants of ST, STh ("human" ST, also referred to as STaII or as STIb) and STp ("porcine" ST, also referred to as STaI or STIa). Regrettably, the nomenclature that uses multiple terms for the same ETEC ST toxins can be quite confusing. However, the fundamental point relevant to the GEMS labs is that the primers used in Multiplex

#1 were intended to amplify both the STh and STp variants. Unfortunately, the GEMS ETEC reference laboratories at the University of Göteborg and University of Chile found that under field use the primers for *estA* were not optimally sensitive for detecting *E. coli* encoding the STp variant.

Sensitivity aside, when a triplet is positive with either of the PCRs that detect *eltB* or *estA*, this indicates the presence within the triplet of ETEC. However, if both genes are amplified, without further testing it is not possible to know precisely what genotypes of ETEC reside within the triplet. For example, the triplet may harbor: LT/ST strains alone; or a mix of LT-only and ST-only strains; or LT-only and LT/ST strains; or ST-only and LT/ST strains. Fortunately, the sites need not worry about the precise genotype of each isolate, since that will be verified in the ETEC references laboratories of Dr. Roberto Vidal (University of Chile) and of Professor Ann-Mari Svennerholm (University of Göteborg).

Since the primers for *estA* in Multiplex #1 were sub-optimal in detecting STp (even though they functioned well for detecting STh, the GEMS Steering Committee on Microbiological Issues recommended that an additional set of primers be used to screen for STp. Therefore, as described below, in GEMS-1A we will also screen all triplets with a Duplex PCR with primers for amplifying STp (and primers for *eae*).

For EAEC --

aaiC – A chromosomal gene that encodes a secreted protein.

aatA – Encodes a protein that facilitates translocation of dispersin across the EAEC outer membrane.

These two gene targets are believed to be the best for detecting EAEC.

For EPEC --

bfpA – This gene, which encodes the structural subunit of bundle forming pili of enteropathogenic *E. coli*, resides on the EAF plasmid.

eae – This chromosomal gene encodes the 94 kd protein intimin.

If both *bfpA* and *eae* are amplified with the primers in Multiplex #1, this confirms that at least one typical EPEC isolate is present in the triplet that was tested. However, it is possible that an atypical EPEC (aEPEC, a strain that is *eae*-positive but *bfpA*-negative) is also present in the triplet or that even an EHEC is also present. EHEC are identified by testing the triplet with Multiplex #2 (see below), which contains primers to detect *stx1* and *stx2* (Shiga toxin). As in GEMS-1, sites will be expected to test triplets that are *eae*-positive but *bfpA*-negative with Multiplex #2 to look for EHEC. In contrast, sites will not be asked to test triplets that are both *eae*-positive and *bfpA*-positive with Multiplex #2 to look for EHEC. GEMS-1 showed that EHEC are rare in GEMS patients, so this would be a lot of expense and work for a low yield at the sites. However, this is not meant to discourage any site from performing the Multiplex #2 when Multiplex #1 is positive for both *eae* and *bfpA* if they have interest in doing so.

GEMS-1A *E. coli* Duplex PCR.

In parallel with Multiplex #1 (or sequentially), all *E. coli* triplets from both cases and controls will also be tested in the Duplex PCR that contains two sets of primers, one set to amplify the gene encoding STp and the other to amplify *eae*. The STp primers and standard operating procedure (SOP) were provided by Professor Ann-Mari Svennerholm, Head of the WHO Collaborating Centre for Research on Enterotoxigenic *Escherichia coli*, in Göteborg, Sweden, while the sequences for the *eae* primers and accompanying SOP were provided by Dr. Marija Tauschek and Professor Roy Robins-Browne of the University of Melbourne. Dr. Tauschek worked out the Duplex method that combines the primers. Whereas each primer set works flawlessly as a monoplex and as a Duplex when a triplet contains either STp or an *eae*-containing *E. coli* (e.g., tEPEC, aEPEC or EHEC), when the triplets contain a mix of both STp and *eae*-positive *E. coli* the *eae* primers lose sensitivity and false negative tests for *eae* occur. Moreover, the optimal conditions for the separate PCRs are such that it was not possible to resolve this problem. Fortunately, based on the GEMS-1 experience the presence in *E. coli* triplets of both ST-producing ETEC and *eae*-positive strains was uncommon. Nevertheless, the work flow in the attached table takes a conservative approach and includes an extra step to assure that *eae*-containing *E. coli* will not be missed if STp *E. coli* are present.

FURTHER EXAMINATION OF TRIPLETS THAT TESTED POSITIVE WITH THE SCREENING PCRs BY TESTING WITH MULTIPLEX #2 OR *bfpA* MONOPLEX

GEMS-1A *E. coli bfpA* monoplex PCR.

Part of the work flow will involve the use of a monoplex for *bfpA*. The work flow table that follows describes when the below monoplex for *bfpA* is to be performed on a triplet of *E. coli* isolates.

GEMS *E. coli* Multiplex PCR #2.

This multiplex contains primers to amplify *stx1*, *stx2*, *eae* (using a different set of primers than found in Multiplex #1), *efa-1* (EHEC factor for adherence) and *sen* (these primers amplify *sen* of EPEC, which is homologous to but distinct from plasmid-encoded *sen* of *Shigella*). EPEC *sen* resides within the chromosomal Pathogenicity Island O122. EHEC *efa-1* is highly homologous to *lifA* of EPEC, which encodes lymphostatin, a protein that inhibits expression of proinflammatory cytokines and contributes to adherence to epithelial cells. The primers for *sen* and *efa-1* are included within Multiplex #2 to assess whether these genes correlate with an increased ability of tEPEC and aEPEC to cause diarrhea or to result in more severe forms of diarrheal disease.

For purposes of discussing the *E. coli* work flow, the attached table will address only the role of primers that detect *stx1*, *stx2* and *eae*. Please note that the *eae* primers in Multiplex #2 generate a 377 bp amplicon, whereas the *eae* primers in Multiplex #1 produce an 881 bp amplicon.

Work-up of *E. coli* triplets in GEMS-1A

This table describes the work flow in relation to the use of the *E. coli* Multiplex #1, the Duplex (primers for *eae* and STp) and the *E. coli* Bfp Monoplex. Multiplex #1 and the Duplex will be both used to test triplets of *E. coli* colonies from all enrolled subjects (cases & controls) in GEMS-1A (**Step 1**). The Duplex results are compared with the *E. coli* Multiplex #1 results on the same triplet (**Step 1**). Additional work proceeds depending on the results of these two PCRs, as described in **Steps 2 & 3**.

The work flow described below takes into account the results of the Duplex and Multiplex #1 screenings of the *E. coli* triplets only with respect to *eae*, STp & *bfp*. It does not address the fact that a triplet positive for STp in the Duplex may also be positive for STh and/or *eltB* in Multiplex #1. Nor does this table's work flow pay attention to the other gene targets of Multiplex #1 (e.g., *aaiC, aatA*).

**Step 1** (Multiplex #1 & Duplex done in parallel or consecutively)		**Step 2**		**Step 3**
**Multiplex #1**	**Duplex**	***bfp* Monoplex**		**Multiplex #2**
***eae*+ *bfp*+**	***eae*+ STp+**	**no more testing**		
	***eae*+ STp−**	**no more testing**		
	***eae*− STp+**	**no more testing**		
	***eae*− STp−**	**no more testing**		
***eae*− *bfp*+**	***eae*+ STp+**	**no more testing**		
	***eae*+ STp−**	**no more testing**		
	***eae*− STp+**	**no**		**Yes**
	***eae*− STp−**	**no more testing**		
***eae*+ *bfp*−**	***eae*+ STp+**	**yes**	**+**	**no more testing**
			**−**	**Yes**
	***eae*+ STp−**	**yes**	**+**	**no more testing**
			**−**	**Yes**
	***eae*− STp+**	**yes**	**+**	**no more testing**
			**−**	**Yes**
	***eae*− STp−**	**yes**	**+**	**no more testing**
			**−**	**Yes**
***eae*− *bfp*−**	***eae*+ STp+**	**yes**	**+**	**no more testing**
			**−**	**Yes**
	***eae*+ STp−**	**yes**	**+**	**no more testing**
			**−**	**Yes**
	***eae*− STp+**	**no**		**Yes**
	***eae*− STp−**	**no more testing**		

0-11 months

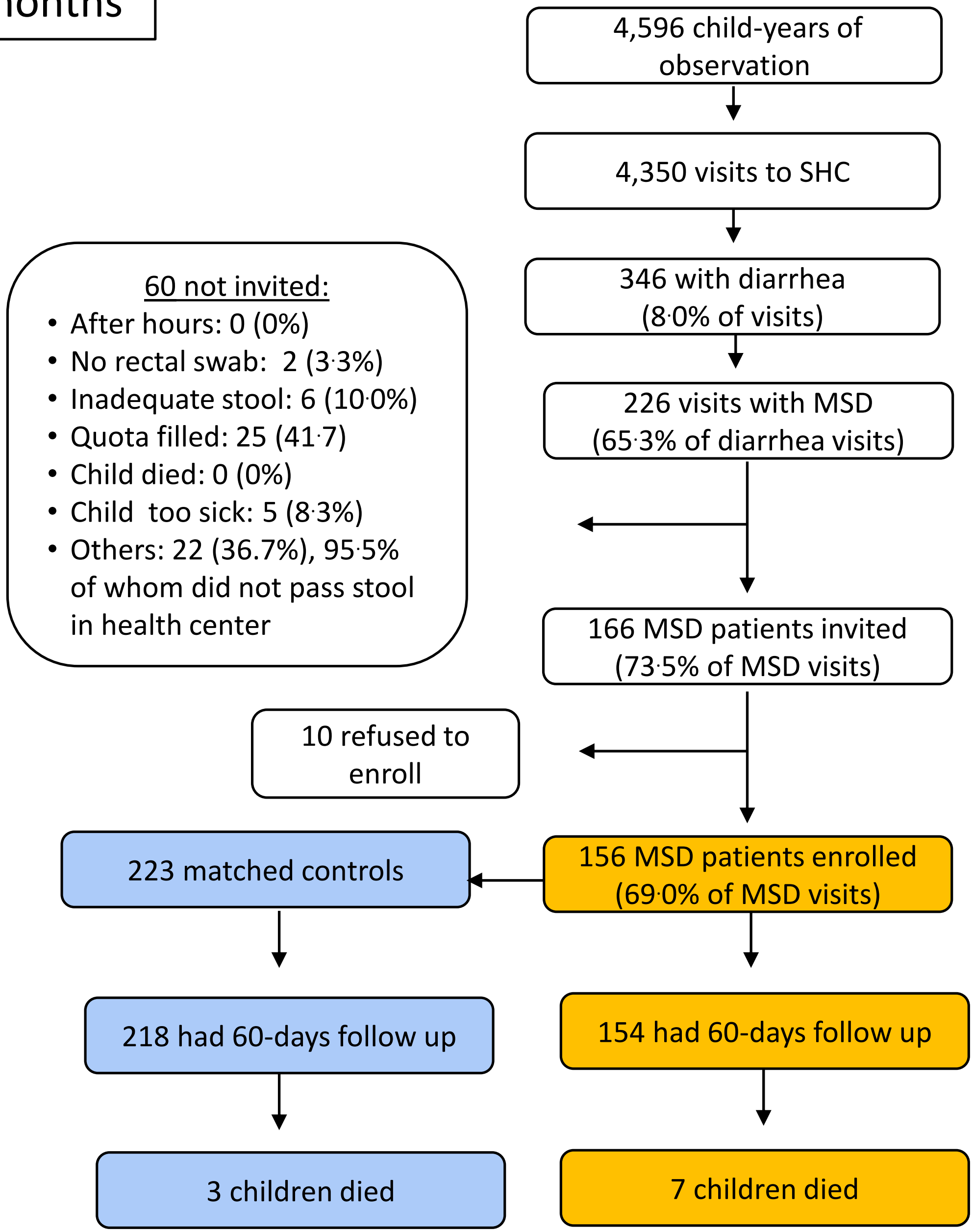
4,596 child-years of observation
4,350 visits to SHC
346 with diarrhea (8·0% of visits)
226 visits with MSD (65·3% of diarrhea visits)
60 not invited:
• After hours: 0 (0%)
• No rectal swab: 2 (3·3%)
• Inadequate stool: 6 (10·0%)
• Quota filled: 25 (41·7)
• Child died: 0 (0%)
• Child too sick: 5 (8·3%)
• Others: 22 (36.7%), 95·5% of whom did not pass stool in health center
166 MSD patients invited (73·5% of MSD visits)
10 refused to enroll
156 MSD patients enrolled (69·0% of MSD visits)
223 matched controls
218 had 60-days follow up
154 had 60-days follow up
3 children died
7 children died

12-23 months

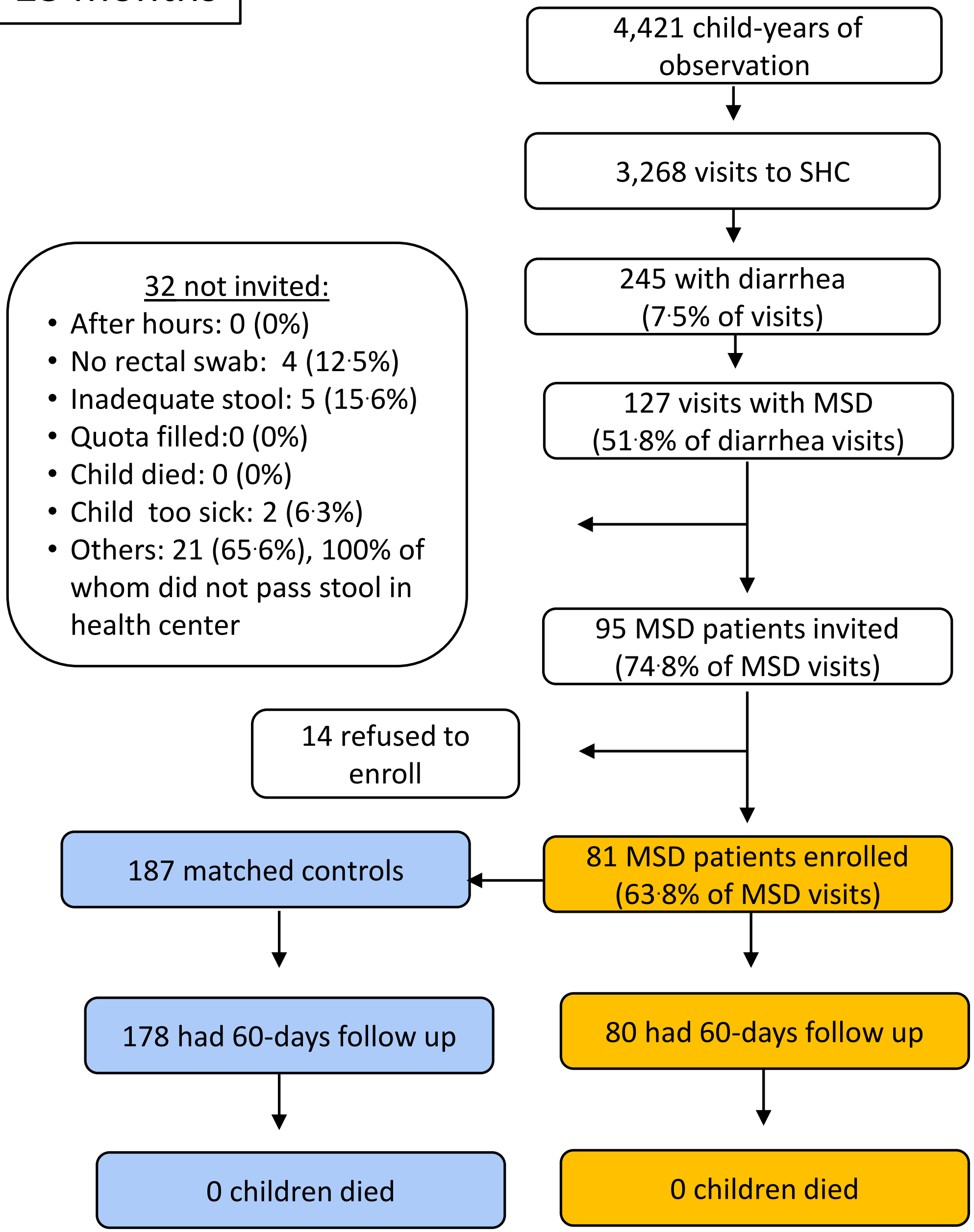
4,421 child-years of observation
3,268 visits to SHC
245 with diarrhea (7·5% of visits)
127 visits with MSD (51·8% of diarrhea visits)
32 not invited:
• After hours: 0 (0%)
• No rectal swab: 4 (12·5%)
• Inadequate stool: 5 (15·6%)
• Quota filled:0 (0%)
• Child died: 0 (0%)
• Child too sick: 2 (6·3%)
• Others: 21 (65·6%), 100% of whom did not pass stool in health center
95 MSD patients invited (74·8% of MSD visits)
14 refused to enroll
81 MSD patients enrolled (63·8% of MSD visits)
187 matched controls
178 had 60-days follow up
80 had 60-days follow up
0 children died
0 children died

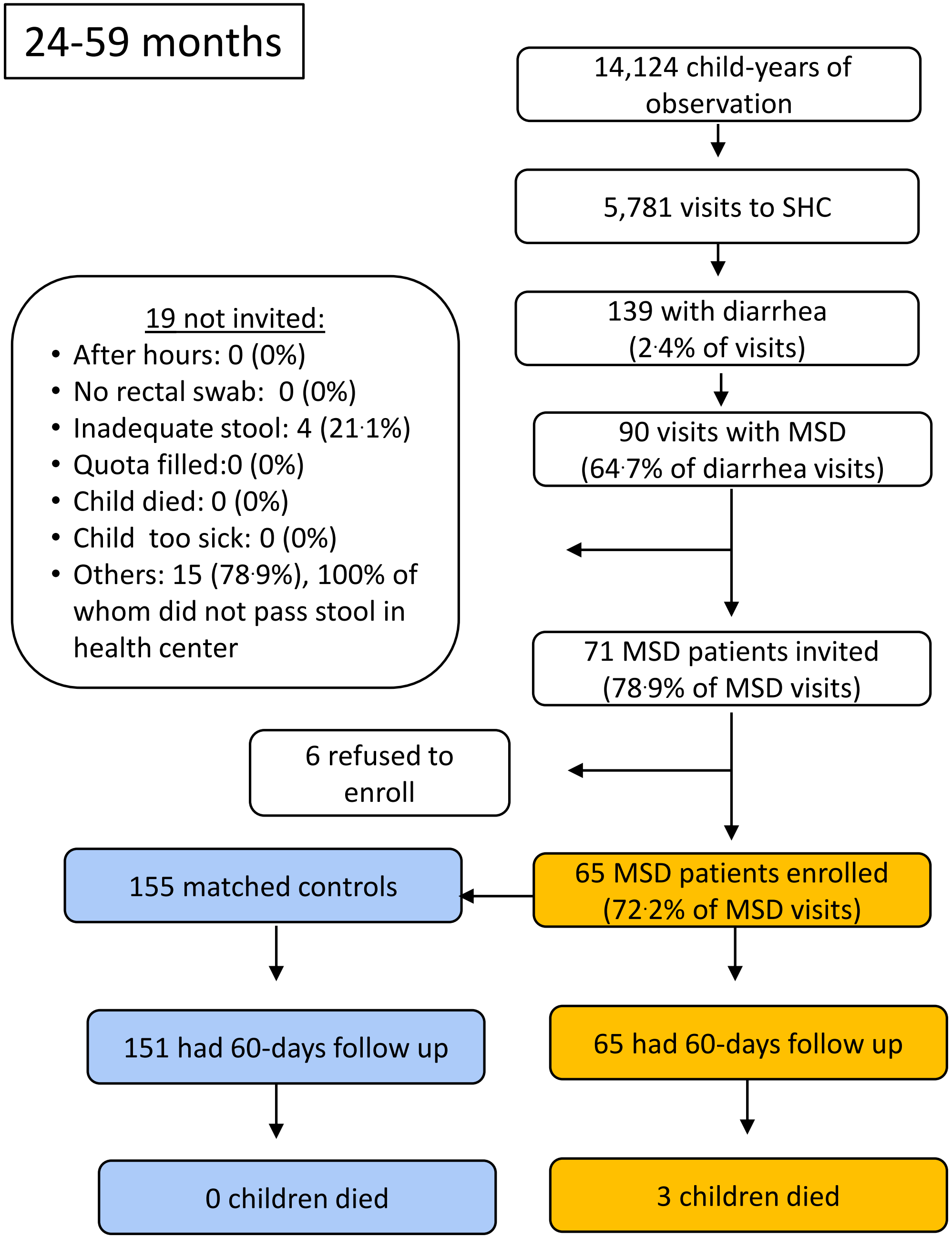
24-59 months
14,124 child-years of observation
5,781 visits to SHC
139 with diarrhea (2·4% of visits)
90 visits with MSD (64·7% of diarrhea visits)
19 not invited:
• After hours: 0 (0%)
• No rectal swab: 0 (0%)
• Inadequate stool: 4 (21·1%)
• Quota filled:0 (0%)
• Child died: 0 (0%)
• Child too sick: 0 (0%)
• Others: 15 (78·9%), 100% of whom did not pass stool in health center
71 MSD patients invited (78·9% of MSD visits)
6 refused to enroll
65 MSD patients enrolled (72·2% of MSD visits)
155 matched controls
151 had 60-days follow up
65 had 60-days follow up
0 children died
3 children died

Various enteric pathogens associated with a higher risk of fatal outcomes in infants and toddlers with moderate-to-severe diarrhoea (MSD) in the GEMS and GEMS-1A studies combined. Hazard ratios (HR) after controlling for exclusive breastfeeding (Compare to Table 3 in the main paper)			
	**Hazard Ratio**	**95% CI**	**p value**
**Age 0-11 months**			
Typical EPEC	2.52	1.65 – 3.86	<0.0001
ST/LT and ST-only ETEC	1.88	1.09 – 3.25	0.024
Aeromonas	3.24	1.25 – 8.40	0.016
**Age 12-23 months**			
Cryptosporidium	1.95	1.13 – 3.39	0.017
Typical EPEC	2.44	1.19 – 5.01	0.015
Enteroaggregative *E. coli*	2.23	1.29 – 3.86	0.0042
Entamoeba histolytica	3.89	1.38 – 10.96	0.0103
